# Characterization of the Small RNA Transcriptome of the Marine Coccolithophorid, *Emiliania huxleyi*

**DOI:** 10.1371/journal.pone.0154279

**Published:** 2016-04-21

**Authors:** Xiaoyu Zhang, Jaime Gamarra, Steven Castro, Estela Carrasco, Aaron Hernandez, Thomas Mock, Ahmad R. Hadaegh, Betsy A. Read

**Affiliations:** 1 Department of Computer Science and Information Systems, California State University, San Marcos, CA, 92096, United States of America; 2 Department of Biological Sciences, California State University, San Marcos, CA, 92096, United States of America; 3 School of Environmental Sciences, University of East Anglia, Norwich Research Park, Norwich, Norfolk, NR4 7TJ, United Kingdom; Mount Allison University, CANADA

## Abstract

Small RNAs (smRNAs) control a variety of cellular processes by silencing target genes at the transcriptional or post-transcription level. While extensively studied in plants, relatively little is known about smRNAs and their targets in marine phytoplankton, such as *Emiliania huxleyi (E*. *huxleyi*). Deep sequencing was performed of smRNAs extracted at different time points as *E*. *huxleyi* cells transition from logarithmic to stationary phase growth in batch culture. Computational analyses predicted 18 *E*. *huxleyi* specific miRNAs. The 18 miRNA candidates and their precursors vary in length (18–24 nt and 71–252 nt, respectively), genome copy number (3–1,459), and the number of genes targeted (2–107). Stem-loop real time reverse transcriptase (RT) PCR was used to validate miRNA expression which varied by nearly three orders of magnitude when growth slows and cells enter stationary phase. Stem-loop RT PCR was also used to examine the expression profiles of miRNA in calcifying and non-calcifying cultures, and a small subset was found to be differentially expressed when nutrients become limiting and calcification is enhanced. In addition to miRNAs, endogenous small RNAs such as ra-siRNAs, ta-siRNAs, nat-siRNAs, and piwiRNAs were predicted along with the machinery for the biogenesis and processing of si-RNAs. This study is the first genome-wide investigation smRNAs pathways in *E*. *huxleyi*. Results provide new insights into the importance of smRNAs in regulating aspects of physiological growth and adaptation in marine phytoplankton and further challenge the notion that smRNAs evolved with multicellularity, expanding our perspective of these ancient regulatory pathways.

## Introduction

Coccolithophores belong to the haptophyte phylum and are one of the major functional groups of marine phytoplankton. As important photosynthetic organisms at the base of the marine food chain, coccolithophores have attracted extensive multidisciplinary interest. They form large mesoscale blooms in the ocean, fixing significant amounts of carbon [1] into organic photosynthetic product and calcified inorganic exoskeletons. By doing so, they facilitate export of dissolved inorganic carbon which decreases alkalinity when the alga die and sink to the seafloor [2]. The ornate calcareous platelets, coccoliths, also may enhance export of aggregated particulate organic matter by ballasting [3,4]. In deep-sea sediments, coccoliths contribute about half the carbonate and, together with the long-chained alkenone lipids produced by select species, they provide a prime record of marine environmental change on timescales of thousands to millions of years [5–7]. While many coccolithophores have restricted distributions, one species, *Emiliania huxleyi* is exceptional in its breadth of distribution. It is the dominant bloom-forming coccolithophore, and one of the most ubiquitous and abundant species of oceanic phytoplankton. For these reasons, it has been the subject of extensive physiological, biochemical, and ecological research [8] and has emerged as a model system for studying biosphere-geosphere interactions.

The recent sequencing of the *E*. *huxleyi* genome represents a milestone in our understanding of the molecular level pathways that drive the biology and diverse distribution of this species [9]. The availability of this information opens new avenues for research, facilitating the development of new tools and techniques to address important questions such as those relating to biomineralization, dimethylsulfide production, conditions that trigger bloom formation, and the complex haploid/diploid life cycle. These and other physiological processes are likely governed by an array of sophisticated regulatory networks involving many players including miRNAs. Here, we take advantage of the *E*. *huxleyi* genome to study small regulatory RNAs.

Small silencing RNAs (smRNAs) play an important role in regulating gene expression at the transcriptional or post-transcriptional level in most eukaryotes, from unicellular to multi-cellular plants and animals [10–13]. They suppress protein expression by triggering the enzymatic cleavage of mRNA and are involved in a variety of cellular processes essential to genome stability, development, and adaptive responses to environmental stresses. These natural smRNA silencing processes have quickly become a powerful tool for the systematic analysis of gene function, and have been targeted as an important resource for therapeutics [14,15].

MicroRNAs (miRNAs) are endogenous small RNAs between 20–24 nucleotides (nt) in length, and are the most extensively studied class of smRNAs. They regulate gene expression post-transcriptionaly by binding to target mRNAs and preventing their translation. Because miRNAs can be expressed at high levels, up to tens of thousands of copies per cell, and effector complexes can be reused, they play an important regulatory role in controlling hundreds of mRNA targets [16]. They have been identified in many plants and animals [11,16–23], and more recently in freshwater and marine algae, including *Chlamydomonas reinhardtii* [24,25], *Thalassiosira pseudonana* [26], *Ectocarpus siliculosus* [27,28] and *Porphyra yezoensis* [29].

Most miRNAs reside in non-coding regions of the genome and are derived from primary transcripts (precursors) that form characteristic hairpin structures [10,17]. In plants, the folded miRNA precursor is processed by a nuclear-localized Dicer protein to generate the miRNA:miRNA* duplex, with 2-nt 3’ overhangs. The hairpin structure of the miRNA precursor and the expressed miRNA/miRNA* duplex at the ends of the arms of the folded hairpin, are two important features that can be used to computationally identify miRNAs in genome wide analyses. The mature miRNA is methylated at the 3’ end, protecting it from degradation and polyuridylation. Upon export to the cytoplasm, the miRNA strand is incorporated into an effector ribonucleoprotein (RNP) complex with the Argonaute nuclease (AGO) protein at its core, and the miRNA* strand is degraded. miRNA RNPs mediate diverse functions dictated by the particular AGO protein member in the complex, and the degree of sequence complementarity between the miRNA and the target mRNAs [30,31]. Most miRNA RNPs with near-perfect complementarity to target mRNAs mediate mRNA cleavage, while RNPs with a greater degree of mismatches inhibit translation and/or trigger the transport of mRNA to cytoplasmic processing bodies (P-bodies) for storage or degradation [11].

High throughput sequencing and bioinformatics analysis have become the standard approach to identify novel miRNAs in organisms for which small RNAs have not been characterized. The Illumina platform employs a sequencing-by-synthesis approach [32] and generates millions of short reads in a single run. It is thus ideal for deep-sequencing small RNAs and is used extensively for detecting miRNAs and other small interfering RNAs (siRNAs) including repeat-associated siRNAs (rasiRNAs) *trans*-acting siRNAs (ta-siRNAs), and natural antisense transcript-derived siRNAs (nat-siRNAs).

In this study, we identified and characterized miRNAs and their targets in *E*. *huxleyi*, by utilizing a pool of Illumina sequenced small RNAs from the *E*. *huxleyi* RNA samples extracted during different stages of growth. The computational analysis of the data predicted 18 novel miRNA candidates and their precursors [33]. Real time PCR was used to experimentally validate miRNA predictions and to quantitate expression in calcifying and non-calcifying cultures. In addition to the miRNAs, several candidate rasiRNAs, ta-siRNAs, and nat-siRNAs were identified.

## Materials and Methods

### E. huxleyi Strains

The *E*. *huxleyi* strains CCMP1516 and M217 used in this study are isogeneic lines (evidenced by the 100% identity of the nucleotide sequences of the cox3, tufA, and the mitochondrial and plastid 16S rRNA gene sequences). Isolated from the South Pacific (02,6667S 82.7167W) in 1991, CCMP1516 was maintained in the Bigelow National Center for Marine Algae and Microbiota (formerly Provasoli-Guillard National Centre for the Culture of Marine Phytoplankton) where over time it lost its ability to calcify. Before becoming a non-calcifier, a subclone of CCMP1516 was sent to the Plymouth Algal Collection and designated M217. Unlike CCMP1516, M217 has retained its ability to calcify in culture. Hence, as isogenic lines, CCMP1516 and M217 represent an ideal model system for studying biomineralization.

### Small RNA library construction

**Fig 1:**
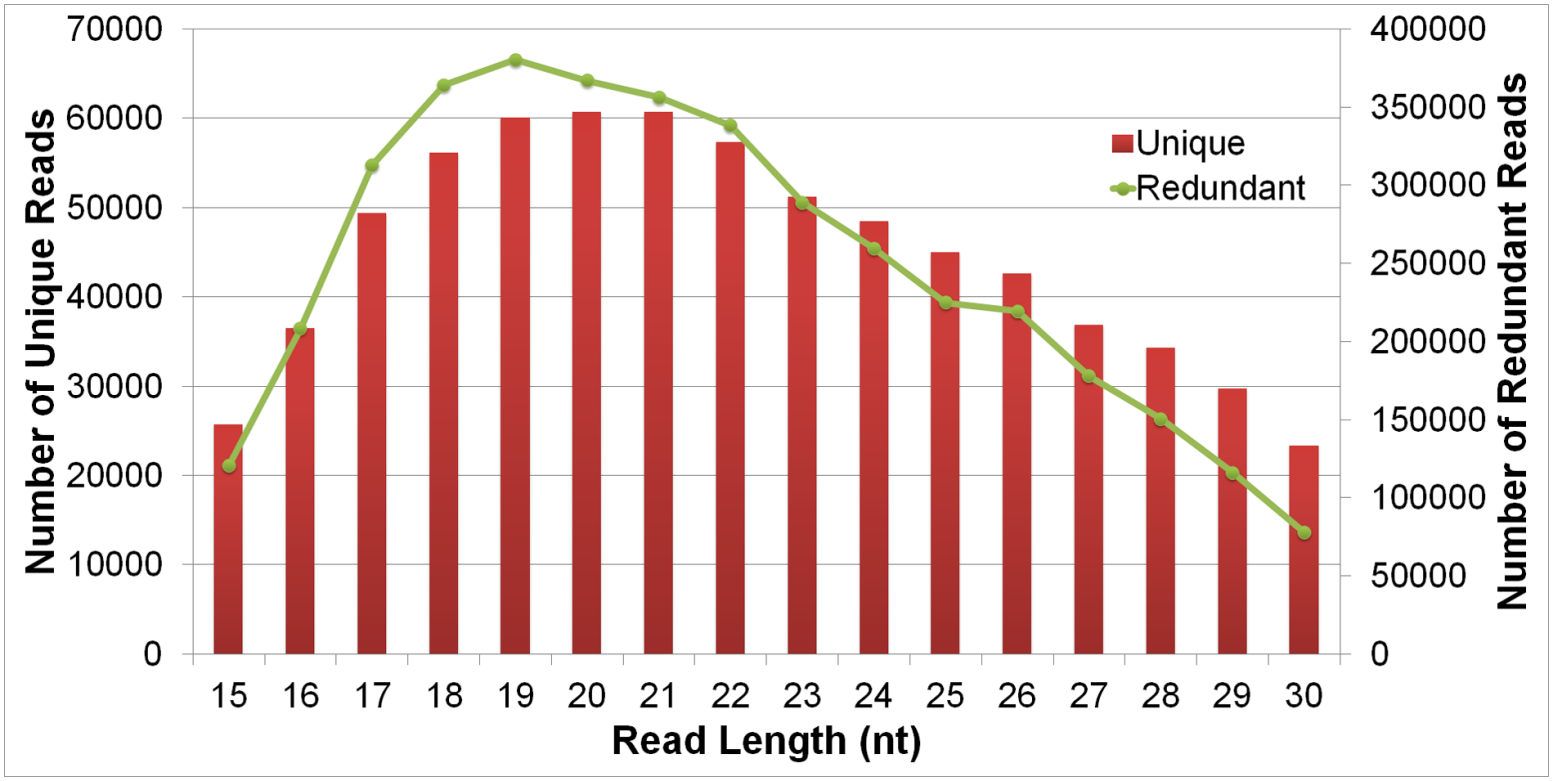
The length distribution of small RNA reads of *E*. *huxleyi*.

*E*. *huxleyi* strain 1516 was grown in artificial seawater with metals and vitamins added to achieve f/2 medium concentrations [34]. Cultures were incubated at 18–20°C under cool white fluorescent light (660 μmol · m^2^· s^2^) on a 12 hr light/dark cycle. Cells were harvested at high density at the end of the growth phase. After inoculation at a density of ~ 5 x 10^4^ cells/ml, RNA was isolated at four, six, eight, ten and twelve days thereafter. At these time points, exhaustion of nutrients and changes in carbonate chemistry would be expected to cause strong changes in gene expression [35–37]. In addition, the physiology of the non-calcified CCMP1516 and calcified M217 would be most divergent, as the liberation of protons by calcification results in a large difference in pH and carbonate chemistry between the two strains at high cell density (S1 and S2 Figs). This strategy was used to enhance the change of detecting a broad range of regulatory elements with roles in physiological differentiation. For RNA extractions, a standard guanidium isothiocyante procedure with a PEG/NaCl precipitation [38] was used. After pooling samples, 23 *μ*g RNA was loaded in two wells of a 15% denaturing PAGE and the 18–30 nt area of small RNAs was excised. After purification, 5’ and 3’-end adaptor sequences were ligated, and small RNAs were amplified by using RT-PCR. PCR products were size selected using denaturing PAGE. PCR products of ~92 nt were purified from the gel, and sequenced using the Illumina platform. After filtering, a library of 3,962,554 clean small RNA (smRNA) reads was obtained with lengths ranging from 15 to 30 nt (Fig 1). Clustering of reads yielded 719,059 unique sequences.

**Fig 2:**
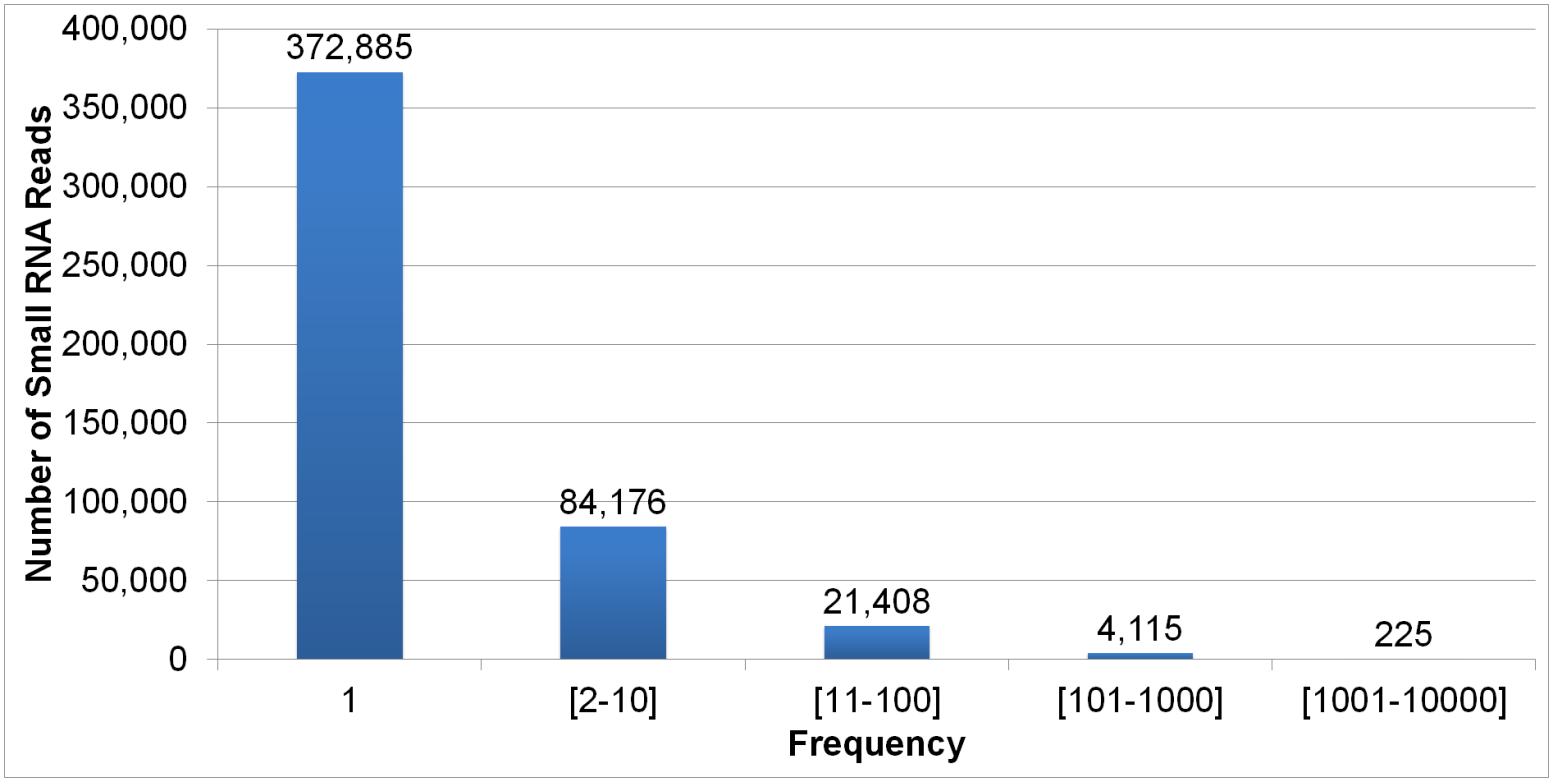
Distribution of unique smRNA reads with lengths between 18 and 26 nt in the smRNA library.

To predict miRNA candidates in the *E*. *huxleyi* genome the 482,809 unique reads between 18–26 nt were used. In the original library the average frequency of unique reads in this size range was ~5.8. As shown in Fig 2, while most reads were expressed at very low levels with just a single copy present in the library, others were expressed at very high levels (max abundance = 9790).

### Filtering smRNA reads

The smRNA reads were filtered to remove those homologous to known transfer RNAs (tRNA) (downloaded from the UCSC tRNA database [39]) and ribosomal RNAs (rRNA) (downloaded from the SILVA rRNA database (version 95) [40]). Perfect matches to tRNA and rRNA sequences were removed, leaving 371,016 (76.8%) unique reads for further analysis.

### Prediction of miRNA candidates

First, homology searches were used to compare known mature miRNAs and precursors resident in MirBase to the *E*. *huxleyi* genome. MiRNA candidates were then predicted based on the expressed smRNA reads. Filtered smRNA reads in the 18–26 nt range were aligned to the reference *E*. *huxleyi* genome, version 1.0 ([http://genome.jgi-psf.org/Emihu1]) using NCBI BLAST and perfect matches were retained. Table 1 shows the number of exact hits to the reference genome for the 18–26 nt smRNA reads. To predict miRNA candidates, windows of 60 to 300 nt surrounding each perfect hit were incremented by 20 nt, extracted and folded with RNAfold [41], and then curated based on MIRcheck [42] criteria. “Good” structures were defined as having: 1) one major loop where at least 50% bases were complementary, 2) no more than four mismatches and two bulges in the putative mature miRNA region, with 3) minimum free energies < -0.35×length kcal/mol, (i.e. -35 kcal/mol for a 100 nt precursor). A Perl script was developed to examine the candidate pre-miRNAs using these criteria along with MIRcheck [42].

**Table 1:** The number of reads mapping to the reference *E*. *huxleyi* genome for the 18–26 nt smRNAs.

Read length	# of Unique Reads	# of Hits to Genome
26	33,557	65,751
25	34,924	72,912
24	37,567	78,730
23	39,478	96,034
22	44,622	104,401
21	47,109	128,772
20	46,589	133,019
19	45,503	126,317
18	41,667	117,580
**Total**	**371,016**	**923,516**

Additional filtering to reduce possible false positives [43] included identifying candidate miRNAs with a frequency of ≥ 3 in the small RNA library that form a duplex with a read on the opposite arm, known as the miRNA*. The miRNAs were defined such that: (1) the miRNA:miRNA* duplex features a two-base overhang at the 3' end of both sequences, (2) the mature miRNA is highly complementary to the corresponding segment on the opposite arm of the hairpin, with four or fewer mismatches within the duplex, and (3) asymmetric bulges in the precursor miRNAs are infrequent and limited to one or two bases. Highly expressed smRNAs (frequency ≥ 100), whose precursors formed “good” hairpin structures but lacked miRNA* on the opposite arm were also included as viable candidates. The candidate mature miRNAs and precursors were screened using a perl script and manually curated.

Further filtering of potential miRNA precursors was done by aligning the precursors to the European Nucleotide Archive (ENA) and NCBI NR nucleotide database. Precursors mapping completely within annotated *E*. *huxleyi* transcripts or with high similarity to annotated ribosomal RNAs were removed.

Additional prediction of miRNA candidates was performed using the miRCat tool included in the UEA Small RNA workbench [44]. Default plant parameters for miRCat were used to analyze the set of 18–26 nt snRNA reads, and only predicted miRNA with high abundance ≥ 7 were retained as candidates.

### Prediction of targets for candidate miRNAs

Target genes for the miRNA candidates were predicted using both plant and animal binding characteristics. miRanda was used to predict targets with animal miRNA binding characteristics [45]. miRanda predictions required perfect complementarity in the seed region (nucleotides 2–8 of the 5’ end of the small RNA) with a binding energy less than -20 kcal/mol. The plant psRNATarget analysis server was used to predict targets with plant-like miRNA binding characteristics [46]. psRNATarget integrates two analyses by (1) searching for near-perfect reverse complementarity between small RNA and target transcript, and (2) integrating target-site accessibility or the unpaired energy (UPE) required to ‘open’ secondary structure around a small RNA target site. The scoring threshold was set to allow no more than 3 mismatches and a maximum unpaired energy (UPE) less than 30 kcal/mol [47].

The refined set of 30,569 *E*. *huxleyi* gene models and their annotations was used for miRNA target prediction. The predicted targets were then annotated using protein blast against the UniProt and NCBI NR databases. Gene ontology (GO) terms for the predicted targets were assigned based on the JGI GO annotation file and identifiers were then clustered.

### Quantitative Stem-Loop RT PCR of miRNAs

To experimentally validate and quantify expression of mature miRNAs in *E*. *huxleyi* stem-loop reverse transcription real-time RT PCR was used. Experiments were performed using small RNAs extracted from calcifying and non-calcifying *E*. *huxleyi* strains M217 and CCMP 1516 respectively, grown in filtered seawater media supplemented with artificial f/2 seawater levels of nitrate, phosphate, vitamins and trace metals (S1 Fig) [34], and cultured as described above. Although the pH was monitored (S2 Fig), no attempt was made to disentangle the combined effects of high cell densities and calcification on the simultaneously changing carbonate chemistry (carbonate, bicarbonate, protons or CO_2_). Small RNAs were first enriched from total RNA using the mirVana miRNA Isolation (Life Technologies, Carlsbad, CA), and polyadenylated using the Poly(A) Tailing Kit (Life Technologies, Carlsbad, CA), at late log/early stationary phase (7 days post inoculation) when nutrients become limiting and calcification is enhanced [48,49]. cDNA was reverse transcribed thereafter using a universal poly(T) stem-loop primer, 5’-TGTCAGGCAACCGTATTCACCGTGAGTGG(T)_18_-3’ where the 5’ stem loop end was designed to provide spatial constraint [50] and the 3’-poly(T) end for binding to the poly(A) tail of the miRNA. Stem loop reverse transcription was carried out as described [51] using the SuperScript III Reverse Transcription PCR Kit (Life Technologies). 10 ng of polyadenylated smRNAs were combined in a 20 μl reaction with 0.5 μl of 10 mM dNTPs, 1 μl of the 1 μM denatured stem-loop RT primer, 4 μl of 5X First-Strand Buffer, 2 μl 0.1 M DTT, 0.1 μl RNAseOUT (40 U/ μl), and 0.25 μl of the SuperScript III RT (200 U/ μl). The reaction was incubated at 16°C for 30 min, followed by 60 pulsed RT cycles of 30°C for 30 s, 42°C for 30 s and 50°C for 1 s. U6 small nuclear RNA was used as an internal control.

Quantitative real-time RT PCR analyses of individual miRNAs were performed with a miRNA-specific primer and a universal primer (S7 Table) using SYBR Green chemistry on the BioRad iCycler iQ real time PCR system. Reactions were assembled in 20 μl and included 1 μl of cDNA template (10 ng), 10 μl 2X Master mix, and 1 μl forward/reverse primers (500 nM each). Cycling parameters began by heating samples to 95°C for 5 min, followed by 35 cycles of 95°C for 5 s and 60°C for 10 s. Product melt curves were generated by heating the reactions to 95°C, cooling to 65°C at 20°C per s, and collecting fluorescence signals as the temperature was increased from 65 to 95°C in 0.2°C increments using a 10 s dwell time. All reactions were carried out in triplicate with a no template control, and experiments were repeated once using separate cell cultures.

For analysis, the cycle threshold, defined as the cycle number where the fluorescence of the amplicon exceeds that of background levels, was determined for individual miRNAs. The relative expression level of each miRNA was compared to that of the internal U6 control using the 2^–ΔCT^ method where the ΔCT = CT _(miRNA)_−CT_(U6 internal standard)_. Variance in miRNA expression across calcifying and non-calcifying strains M217 and CCMP1516, respectively, was determined using the 2^ΔΔCt^ method where ΔΔCt = CT_(M217 miRNA_–_M217 U6 ref)_−CT_(CCMP1516 miRNA—CCMP1516 U6 ref)_. PCR efficiency calculations were performed using *E* = 10^(-1/slope) [52] and melt curve analysis enabled distinct miRNA product melt peaks to be distinguished from those of primer dimers.

### Real Time RT PCR of predicted miRNA target genes

Real Time RT-PCR primers were designed to a subset of miRNA target genes from mir06, mir15 and mir18 using Primer3 software version v.0.4.0 (http://bioinfo.ut.ee/primer3-0.4.0). Primers were selected to have a GC content of 55–65%, a melting temperature between 56–61°C, with a target amplicon of 75–100 base pairs. Primers were synthesized by Integrated DNA Technologies, Inc. (Coralville, Iowa, USA). Total RNA (1 μg) was extracted from *E*. *huxleyi* strains M217 and CCMP 1516 grown as describe above. After treating with DNAse I (Ambion), mRNA was reverse transcribed to prepare cDNA using oligo d-T primers and the Omniscript RT Kit (Qiagen). Reactions were assembled using 2 μg template RNA, 1X RT buffer, 0.5 mM deoxynucleoside triphosphate, 1μM oligo(dT) primer, 10 U of RNase inhibitor and 4 U of Omniscript reverse transcriptase, in a total reaction volume was adjusted to 20 μl. The reaction was heated to 65°C for 5 min to denature the RNA and then, incubated at 37°C for 1 hour.

For real time PCR, the cDNA was diluted 1:40 and reactions were assembled using SYBR green chemistry. Reactions were carried out in a 96-well plate in a 25 μl reaction volume containing 7.1 μl SYBR green Supermix (BioRad, Richmond, CA), 12.3 μl dH_2_O, 0.3 μL of each forward and reverse primer (20 μM) and 5 μL of cDNA. Reactions were run on the iCycler IQ (Bio-Rad, Richmond, CA) with a thermal profile of 95°C for 3 minutes followed by 40 cycles of 95°C for 15 s and 60°C for 30 s. Melt-curve analysis was achieved by reducing the temperature to 55°C for 10 s and ramping 0.5°C every 10 s for 90 cycles. Reactions were run in triplicate alongside a negative, no-template control, with three experimental replicates. PCR specificity was confirmed by melt curve analysis and relative gene expression values were computed using the 2^-ΔΔCT^ method relative to the control gene, PID 447254.

### Prediction of endogenous siRNA candidates

Three types of endogenous siRNAs including phased trans-acting siRNAs, repeat-associated siRNAs and natural antisense transcribed siRNAs, were also investigated using the small RNA dataset.

Trans-acting siRNAs (ta-siRNAs) are typically 21 nt in length and processed from double-stranded RNA (dsRNA) precursors predominantly in a single phase register. Precursors are characterized by phased alignments of 21 nt smRNAs with a two-base overhang on the anti-sense strand. The ta-siRNAs were predicted based on the algorithm of Chen and co-workers [53] where the number of phased and total alignments to a dsRNA window anchored by each unique smRNA sequence is calculated, and the probability of the phasing being significant is determined by comparing to a hyper-geometric distribution[54]. Probabilities (p-values) ≤ 0.001 were considered significant in this study.

Other types of endogenous siRNAs including repeat-associated siRNAs (ra-siRNAs) and natural antisense transcribed siRNAs (nat-siRNAs) were also predicted. Ra-siRNAs were characterized by aligning small RNAs to the repetitive and transposable elements masked by the RepeatMasker in the *E*. *huxleyi* genome. Nat-siRNAs originate from transcribed overlapping sense and antisense genes [11], and generally auto-silence transcripts from which they are derived. Hence, smRNAs mapping to antisense exon regions were used to predict potential nat-siRNAs in *E*. *huxleyi*.

PIWI-interacting RNAs (piRNAs) are 26–30 nt and hence are longer than miRNAs and siRNAs. piRNAs bind to argonaute proteins of the Piwi clade and in animals are known to provide protection from invasive transposable elements [55]. To identify candidate piRNAs in *E*. *huxleyi*, sequence reads between 26–30 nt in the smRNA library were compared using BLASTN to the known human and rat piRNAs housed in piRNABank [56]. Because piRNAs exhibit little sequence homology across species, piRNApredictor, a k-mer scheme which employs a Fisher Disciminant algorithm with greater than 90% precision and 60% sensitivity [57], was also used.

### Identification of candidate genes for RNA interference (RNAi) machinery

A test set of RNAi-related proteins was created by downloading the amino acid sequences for all of the DCL, AGO, RDR, DSRM, HEN1, SID1, HASTY, HLY1, loquacious, SDE-3, VIG, Fmr1, Ambergine, Armitage, MILIm pasha, PSRP1, serrate, and TUDOR proteins from the National Center for Biotechnology Information (NCBI) RefSeq database. To assess sequence homology to these RNAi-related proteins, the *E*. *huxleyi* reduced (haploid) gene models were downloaded from the JGI website and a tBLASPn alignment was performed using the test set of protein sequences. Results were scrutinized based on the bit scores and alignment length. For each Refseq protein, only the top hits with significant e-value (< 1e-6) were then selected for annotation. The top hit genes were reciprocally blasted against the NCBI NR database using blastx (cutoff e-value = 1e-6), with only the top-hit protein recorded in the table. The genes were then translated into protein sequences and conserved domains were searched using the interproscan. Finally potential annotations of the genes were generated using the blast2go pipeline.

## Results and Discussion

### Homology search for known miRNAs

When mature miRNAs from the miRBase (release 16) were mapped to the *E*. *huxleyi* genome, 61 alignments were retained and neighboring sequences were folded and analyzed based on precursor characteristics. Of the 61 alignments, 18 were found to have plausible hairpin structures with 15 homologous to animal and three homologous to plant miRNAs (S1 Table). While several of the miRNA homologs mapped perfectly to the *E*. *huxleyi* genome and corresponding hairpin structures were predicted (defined in the Materials and Methods), mature miRNAs were not detected in the smRNA expression library. Although we cannot eliminate the possibility of false negatives, hairpin sequences were eliminated when both criteria were not met. The only algal miRNA that mapped perfectly to the *E*. *huxleyi* genome was cre-miR1171 from the green alga *Chlamydonomas reinhardtii*. The homolog was not detected in our smRNA library, however, because miRNA expression patterns are dynamic we reasoned that this particular miRNA may simply be expressed at low levels or only under specific ecophysiological conditions, and hence it was retained for further analysis as mir01 in Table 2.

**Table 2:** Predicted miRNA candidates. Mir01 was homologous to *Chlamydomonas* cre-miR1171 and the others were based on small RNA reads.

ID	miRNA Sequence	Length	miRNA Abundance	miRNA* abundance	Precursor Length	Genomic region
**mir01**	TGGAGTGGAGTGGAGTGGAGTGG	23	-	-	82	Intergenic
**mir02**	CGTCCTAATCCTTGGCCTG	19	3	10	170	intron
**mir03**	CGGGAGGGGGAGGGAAGGCT	20	141	NA	193	Intergenic
**mir04**	GGAAGGCTGAGTGCTGCATGT	21	1459	NA	252	intron
**mir05**	CGGCTGGCTGCGCGGGGACTACG	23	317	190	79	Intergenic
**mir06**	CAGAAACTCACGGACCTCGAC	21	27	NA	76	Intergenic
**mir07**	AGTGGATAGCGTGTTGGG	18	10	NA	82	Intergenic
**mir08**	CAACCATGTGGCGTCGGCACA	21	11	NA	114	Intergenic
**mir09**	GCGGCCCTCGAACGGACACCGG	22	10	NA	80	Intron
**mir10**	CGGAGGAGATGTGCCGTTCCG	21	120	NA	144	Intergenic
**mir11**	CATGTAGGTCGCGAACGGGTT	21	8	NA	119	Intergenic
**mir12**	GTCAGTGAGGACGCTGTATCAAGG	24	12	NA	94	Intergenic
**mir13**	GCGTCAGTCGACCGTGGCACATCC	24	7	1	93	Intron
**mir14**	CGAGGTGTCCGAAGATGTATG	21	15	NA	89	Intergenic
**mir15**	GTGGCTAGTATCCGACGCTGTC	22	11	NA	127	Intergenic
**mir16**	AAGAAGGCCGTGGCGCAACTG	21	75	1	92	Intergenic
**mir17**	CGGGGCTGCCGATTCATCGGT	21	54	NA	85	Intergenic
**mir18**	CGTTGATGAGGCCGATCTGGT	21	21	NA	95	Intergenic

### Prediction of miRNA candidates based on small RNAs

MiRNA candidates were predicted from the unique set of 18–26 nt smRNA sequence reads mapped to the genome. Only reads present in three or more copies were considered as putative miRNA candidates. Moreover, because of the complex composition of the *E*. *huxleyi* genome and the likelihood of false positives in predicting secondary structures with such a high GC content, very stringent criteria were used to identify miRNA precursors. 18 miRNA precursors were predicted following the steps outlined in Materials and Methods (S3 Fig), including mir01 from homology search, however only four have miRNA*. This was also observed for miRBase entries cre-MIR907 and cre-MIR910 from *Chlamydomonas* where stem loops were predicted based on highly expressed smRNAs in the absence of an identifiable sequenced miRNA*. The 18 predicted miRNA are listed in Table 2. The copy number of the candidate miRNAs ranged from 3 to 1,459 in the smRNA library, indicating a large variation in potential miRNA expression levels under growth conditions designed to simulate bloom conditions when cell densities are high, nutrients become limiting, and calcification is expected to be elevated. The median length of the candidate miRNAs is 21 nt with lengths of all between 18 to 24 nt. Predicted precursors ranged from 71 to 252 nucleotides, with an average length of 114 nucleotides and a median length of 93 nucleotides (S2 Table). This broad variation is consistent with that of plant miRNA precursors, which range from 50 to more than 350 nt as compared to the shorter 70–80 nt sequences typical of animals [58]. Most of the *E*. *huxleyi* precursors are within the 70–132 nt range observed for pre-miRNAs in *Thalassiosira pseudonana* (*T*. *pseudonanna*) [26], and are shorter than those observed in *Chlamydomonas* where the majority are between 150–729 nt [24].

When sequence homology searches were performed across the candidate miRNAs and corresponding hairpin structures, two of the miRNAs and their precursors show considerable similarity. Mir03 and mir04 are 20 and 22 nt in length, respectively, and share an 8 nt overlap region. Although the precursors show 100% identity over a 109 nt region, the pre-miRNA for mir03 is 193 nt while that of mir04 is 254 nt. The precursors, moreover, reside on separate scaffolds and the miRNAs exhibit different expression profiles and appear to target distinct sets of genes. Therefore, both mir03 and mir04 were retained as potential *E*. *huxleyi* miRNA candidates.

When predicted *E*. *huxleyi* miRNAs and their precursors were mapped relative to the annotated genes, 78% were found in intergenic regions, and 22% were found in predicted introns (Table 2). The large proportion of miRNAs residing in intergenic regions is characteristic of plant miRNAs [18] and less like the miRNAs of *Chlamydomonas* which are typically found in introns [25] and those of *T*. *pseudonana* which are found primarily in exons [26]. Based on their distribution it would appear that approximately one-fifth of the *E*. *huxleyi* miRNAs are transcribed from the promoter of particular host genes, while four-fifths may be transcribed from independent promoters enabling separate control for their transcription by means of transcription factors, enhancers, silencing elements, and chromatin modification. The miRNA precursors were not aligned completely within annotated *E*. *huxleyi* transcripts, or similar to known ribosomal RNAs (S3 Table).

To determine the degree of miRNA conservation, a homology search using the 18 *E*. *huxleyi* miRNA candidates against miRBase was performed. None of the candidates showed significant homology of more than 13 nt to mature miRNAs in the miRBase. When the 18 corresponding *E*. *huxleyi* pre-miRNA hairpins were compared, there was also no significant sequential or structural homology to miRBase precursor sequences. With the majority of *E*. *huxleyi* miRNAs found in intergenic regions, typically not subjected to selective pressure, it is reasonable to assume that sequence preservation may be low, particularly outside mature miRNA regions. Moreover, because *E*. *huxlyei* is a unicellular coccolithophore belonging to the haptophyte phylum that is currently not represented in the miRBase, it is likely that the predicted *E*. *huxleyi* miRNAs provide a novel set of miRNAs to the database.

Because *E*. *huxleyi* possesses significant intra-species variation in genome content [9,59,60], to better understand the function and evolution of *E*. *huxleyi* miRNAs and their cognate precursors, we examined the conservation of these sequences across three deeply sequenced strains (S4 Table). Of the 18 pre-miRNAs predicted in strain 1516 (isolated in 1991 from the South Pacific) 14 were detected in VAN556 (isolated in 1984 off the coast of Vancouver, BC), 13 were detected in EH2 (isolated in 1990 from the Great Barrier Reef, Australia), and 12 were detected in strain 92A (isolated in 1957 from the English Channel), with maximum e-value < 1e-9. However, some miRNAs and their pre-miRNAs were much more conserved across these strains than others. For example, 7 out of the 18 miRNAs perfectly matched to the genomes of all three strains (S5 Table), while several others have no significant alignments of pre-miRNAs or matches of the short miRNAs. The high degree of conservation of these 7 sequences that reside predominately in intergenic regions of the *E*. *huxleyi* genome typically under less selective pressure, suggests the sequences may be of functional significance. Despite their relative abundance in CCMP1516, the absence of some miRNAs across all other strains is consistent with the variable genome and ecophysiology of *E*. *huxleyi*.

### Target Prediction for the candidate miRNAs

Identifying target genes is critical to ascertaining the function of miRNAs. Target genes were predicted for the 18 miRNAs characterized in this study, using plant like binding characteristics. Plant-like binding characteristics revealed 424 possible targets, representing 417 unique genes (S1 File). When predictions are made based on the plant-like binding characteristics, the numbers of targets per miRNA range from 2 to 107, with a median of 16 (S4 Fig). MiRNA binding sites reside predominately in the coding region of the target genes, with only 10% positioned within the intergenic regions.

The target genes were clustered according to second level Gene Ontology (GO) terms with respect to biological processes and molecular function (S5 Fig). Target genes were grouped into the cluster of a second-level GO term if that GO term was an ancestor node of its annotations. It is important to note that target genes can be in multiple clusters of different second-level GO terms. Further attempts to predict the function of the target genes revealed 52.5% have no KOG, 85.7% have no KEGG, and 52.2% have no GO assignments; 37% have neither KOG, KEGG, or GO assignments. The majority of target genes with predicted functions, on the other hand, appear to be involved in metabolic process, cellular process, localization, and biological regulation. These categories, however, also represent the majority of annotated gene functions for *E*. *huxleyi*. When normalized to the number of genes annotated in each functional category, target genes related to cellular localization and biological regulation seemed to be over-represented. Experimentally validating these target gene predictions and their functional significance will be paramount for future studies.

To determine which if any of the predicted target genes may be linked to calcification, comparisons were made to previous studies profiling gene expression patterns in haploid (un-calcified) and diploid (coccolith bearing) life-cycle stages of *E*. *huxleyi* [35–37]. 55, or approximately 13%, of the predicted miRNA target genes (S6 Table) identified herein were amongst the 4289 differentially expressed genes detected in microarray based transcriptomic analyses [37]. While 28 of the predicted miRNA targets were found to be up-regulated in the calcifying diploid life-cycle stage, most have no unigene or nr descriptions, with the exception of one gene predicted to encode a V-type proton ATPase subunit, and another showing homology to transmembrane protein of unknown function. Proteins encoded by two other genes were classified according to KOG as belonging to C-type lectin and collagen clusters, orthologs of which are commonly ascribed to biomineralization. None of the predicted miRNA target genes were among either the coccolith associated protein, GPA, or any of the biomineralization-related ion transport genes characterized by Mackinder and colleagues [36] or Taylor and co-workers [35].

### Stem Loop RT-PCR validation and quantification of miRNAs

Stem-loop RT-PCR was used to confirm the accuracy and reliability of the miRNA predictions, and to profile the expression of the miRNAs in calcifying and noncalcifying *E*. *huxleyi* cells. Expression was detected for all of the predicted miRNAs, including mir01, which shows homology to cre-miR1171 from *Chlamydomonas* but was not detected in our smRNA library. No signal, however, was detected for other MirBase entries exhibiting perfect homology to sequences in *E*. *huxleyi* genome with corresponding hairpin-like precursors (hsa-miR-920 and oan-miR-1331).

Single stem-loop amplification products with melting temperatures approximating the expected values were noted for nearly all of the miRNAs, with the exception of mir12, which exhibited a broad melt curve with two peaks or amplification products (S7 Table). The actual melt temperature of several of the *E*. *huxleyi* stem loop products (10) was >4 but less than 6°C higher than the expected value suggesting length or sequence variants arising potentially from variability in the Dicer and Drosha cleavage positions or the addition of 3’ non-template nucleotides [61,62]. PCR efficiencies of the different miRNAs were comparable (S7 Table), and ranged from 95.8–99.2 with a median value of 98.1.

**Fig 3:**
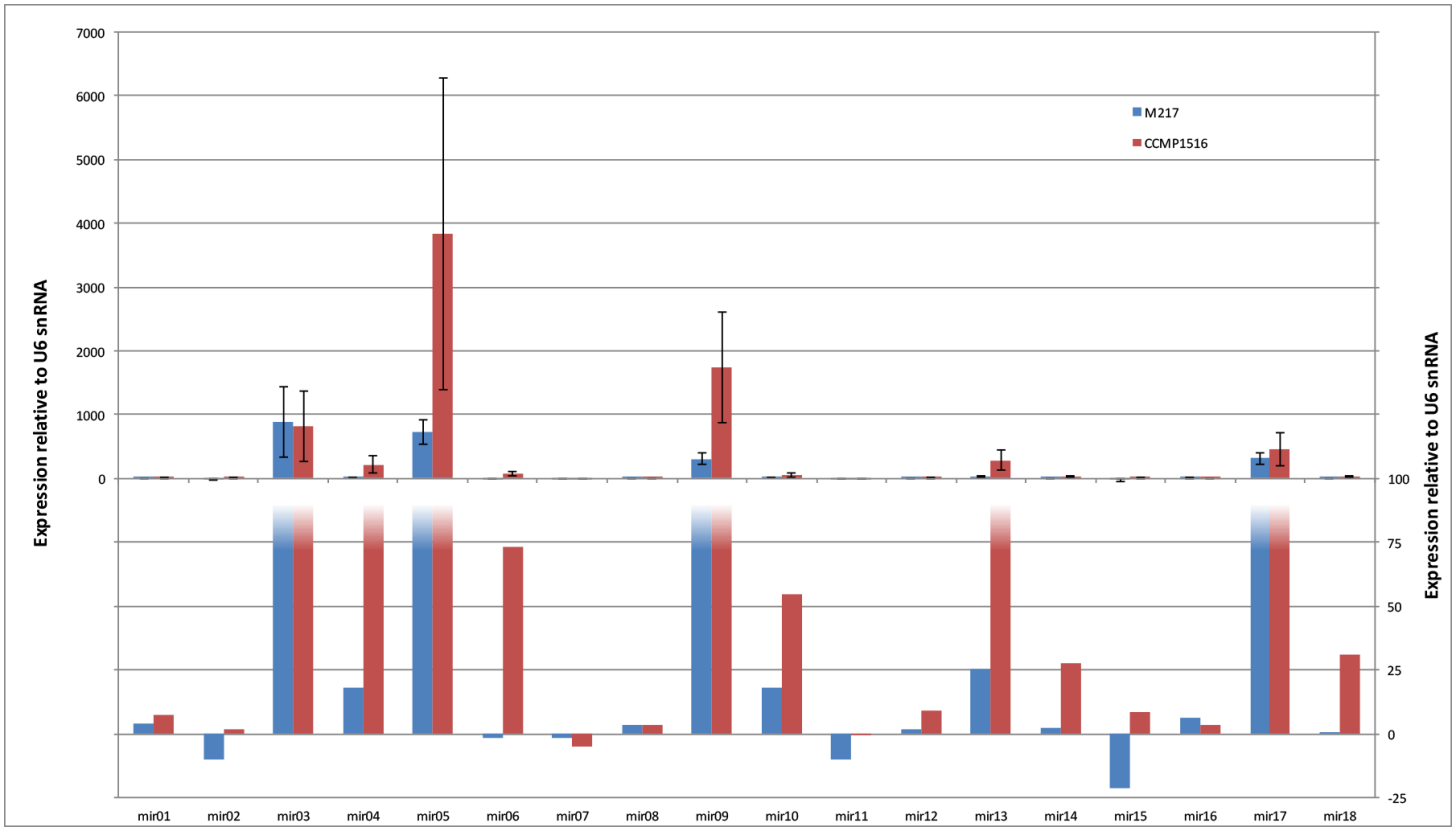
Expression of miRNA Precursors in calcifying and non-calcifying *E*. *huxleyi* cells grown in filtered seawater media. Expression values are relative to that of the, U6 small nuclear RNA reference gene and were determined using the 2^Δct^ method.

The stem-loop RT-PCR assays exhibited a dynamic range of three orders of magnitude, with the expression of most of the miRNAs exceeding that of the U6 small nuclear RNA control. In general, although not as high, the expression pattern of the miRNAs in M217 was similar to that of CCMP 1516 with the exception of mir02, mir06, and mir15 (Fig 3). The most highly expressed miRNAs in both the calcifying and non-calcifying cultures were mir03, mir05, mir9, and mir17. These miRNAs are all expressed at levels greater than 100X that of the U6 reference control suggesting they may be involved in many fundamental functions. For example, with 107 predicted targets, mi03 is associated with the regulation of a broad range of transcripts from those involved in signal transduction to those involved inorganic ion transport, the cell cycle, transcription and posttranslational modification. Despite being more highly expressed mi05 and mi09 have fewer predicted targets (27 and 9, respectively), many of which are of unknown or general predicted function. Mir05 and mir09 also showed considerable variation across experimental replicates, which may be due to different iso-miRNA species. Mir02, mir07, and mir11 were expressed at the lowest levels in both M217 and CCMP1516.

**Fig 4:**
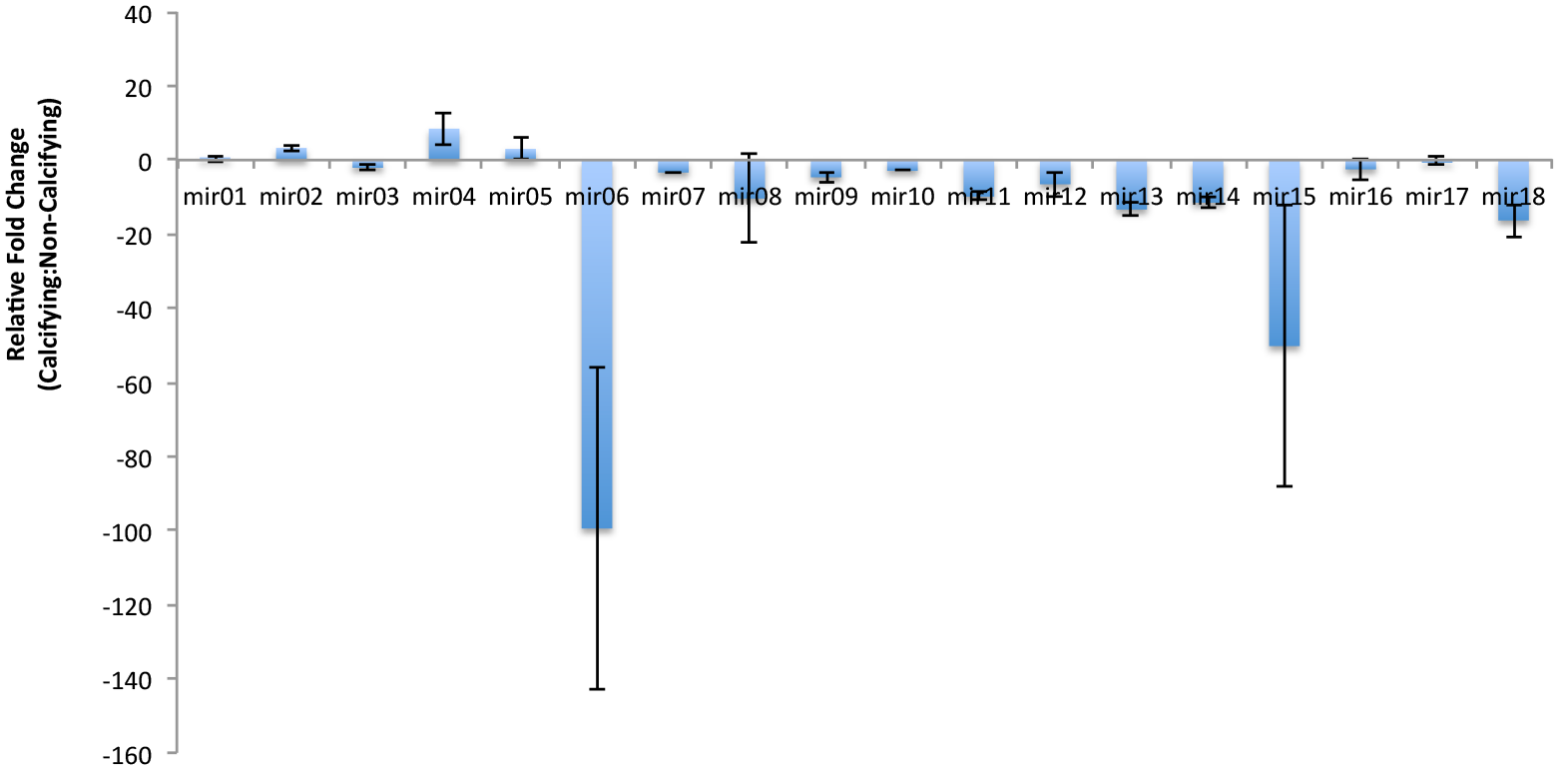
Differential expressions of miRNA in calcifying versus non-calcifying cells. Cultures were grown in filtered seawater media, expression normalized to the U6 small nuclear RNA, and variance determined by the 2^**ΔΔCT**^ method.

Several miRNAs exhibited significant differences in abundance in calcifying M217 as compared to the non-calcifying 1516 cultures (Fig 4). Mir6 and Mir15 exhibited the most variance and were down-regulated 99X +/- 43X and 50X +/- 37X, respectively. Mi6 only has two predicted target genes, both of which are of unknown function. Mir15 targets 11 genes, none of which have sufficiently strong annotation to link them to biomineralization in a meaningful way (S1 File). Evidence is available for the predicted function of two mir18 targets possibly involved in biomineralization. These include genes that code for a protein with significant homology to a calmodulin-like protein with a voltage gated ion channel and a galactose binding domain (PID 462160); and a KOG classified collagen type protein with 6 predicted O-GalNAc (mucin type) glycoslation sites (PID 101602) (S1 File). While calmodulin is ubiquitous among eukaryotes as a primary calcium sensor and second messenger molecule, calmodulin-like proteins have evolved unique functions one of which in the pearl oyster is linked to biomineralization. In the pearl oyster, a calmodulin-like protein is a component of the organic layer where in the presence of a 16-kDa protein it can induce aragonite nucleation and influence calcite growth in the prismatic layer [63]. Mucins, moreover, have long since been associated with controlling crystal growth in the nacre of mollusks [64], and in the ossified skeletons of echinoderms [65] and vertebrate bone, teeth and cartilage [66]. If these sequences are involved in biomineralization it would make sense for Mir18 to be down-regulated as it is in the calcifying M217 where its expression was found to be 16.3X +/- 4.5 lower than in CCMP 1516. Because it is down-regulated by just 2.07 +/- 0.7, it is questionable whether Mir03 is actually differentially expressed in M217 versus CCMP1516. Nonetheless, one of the targets is noteworthy as it shows strong homology to the Coccolith scale associated protein, from *Chrysochomulina tobin*. The function of this particular protein, which is associated with both mineralized and unmineralized scales, however, remains to be defined. Mir04 was up-regulated in M217 8X +/- 4X and although none of the 26 putative targets can be linked to biomineralization at this time, and 50% are without a putative function, they represent important transcripts whose protein products warrant further investigation. Although not intuitively obvious, it would not be unusual for genes related to biomineralization to be down-regulated under calcifying conditions if the protein encoded were to: 1) have a direct role as a negative regulator of calcification, 2) act as a inhibitor of calcification-relate gene expression, or 3) be subjected to translational as opposed to transcription or post-transcriptional control.

It is important to caution that at this stage the observed expression differences between the calcifying and non-calcifying strain cannot be attributed specifically to calcification mechanisms. The high cell densities amplify physiological differences between cells as calcification releases protons from dissolved bicarbonate. This resulted in pH differences between the two strains of 0.4–0.5 units at the time of harvesting, reflecting dramatic differences in carbonate chemistry. Differential expression of miRNAs and the target genes they regulate be linked either to calcification or to cellular and molecular responses to nutrient limitation, high cell densities, and/or carbonate chemistry owing to the complexities of the experimental growth conditions.

Although not evidence of direct interaction, quantitative real time RT-PCR was used to determine whether there is an inverse relationship between the expression of a small subset of miRNAs and their corresponding target genes. The trend in expression is as expected. Both of the targets of Mir6; 7 of the 8 targets tested of Mir 15; and 14 of the 18 targets of Mir18 tested (including PID 101602 and PID 462160 mentioned above), exhibited higher expression (> 2-fold) in the non-calcifying strain CCMP 1516 as compared to 217 (S6 Fig, S8 Table). While some of the target genes showed only modest changes in expression, several were up-regulated by more than two orders of magnitude.

### Prediction of endogenous siRNAs

After characterizing microRNAs, analysis of small interfering RNAs (siRNAs) was performed. Repeat-associated siRNAs (ra-siRNAs) were predicted by aligning the small RNAs with lengths from 18 to 26 nucleotides to the repetitive regions in the *E*. *huxleyi* genome defined by RepeatMasker [67]. 6,731 unique small RNAs mapped perfectly to the repetitive regions in the *E*. *huxleyi* genome, representing 123,391 alignments or 5.7% of aligned unique reads between 18 to 26 nts. These unique small RNAs correspond to 6.41% of the sequences in the redundant set, which is slightly lower than the percentage of annotated repetitive elements in the *E*. *huxleyi* genome. While approximating the proportion of ra-siRNAs detected in the repetitive sequence of *Chlamydomonas* (6.11%) [25], the *E*. *huxleyi* ra-siRNA fraction is significantly lower than that detected in *T*. *pseudonana* (15.9%) [26]. Unlike other siRNAs, ra-siRNAs function at the transcriptional level, silencing or promoting the formation of heterochromatin by directing the methylation of specific DNA sequences.

After masking the repetitive regions of the genome, 16 ta-siRNA-producing loci (TASs) were identified using the srna-toolkit [54] and comparing the probability of phasing to a hyper-geometric distribution [53]. This is slightly larger than the number of ta-siRNAs identified in *Chlamydomonas*, where 11 phased ta-siRNAs were identified. The ta-siRNAs of *E*. *huxleyi* are arranged in 21 bp phased increments in a 231 bp window at TASs, with p-values varying from 7.72E-04 to 8.20E-05 (S9 Table). Most of the predicted ta-siRNAs were detected at a single locus in the *E*. *huxleyi* genome, however, one exceptional group was detected at 119 loci. The distribution of TAS is skewed with the vast majority found in non-coding regions, and only three contained entirely within exons. Assuming the phased smRNA in the 16 TASs are all ta-siRNA candidates, a total of 375 *trans*-acting targets (S2 File) were identified when searching the *E*. *huxleyi* transcriptome for sequences complementary to the 38 unique ta-siRNAs by using the srna-toolkit [54]. These trans-acting targets are involved in a broad spectrum of cellular processes including energy metabolism, transport, single transduction, and transcriptional regulation (S7 Fig). The presence of putative homologs of RNAi components in *E*. *huxleyi* (S10 Table) suggests that these phased siRNAs are produced by a mechanism similar to that used by plant ta-siRNAs, whereby biogenesis requires a RNA-dependent Polymerase, Suppressor of Gene Silencing, and a Dicer-like protein. Because of the low abundance of candidate ta-siRNAs, further study is needed to examine the mechanism and significance of these siRNAs.

**Fig 5:**
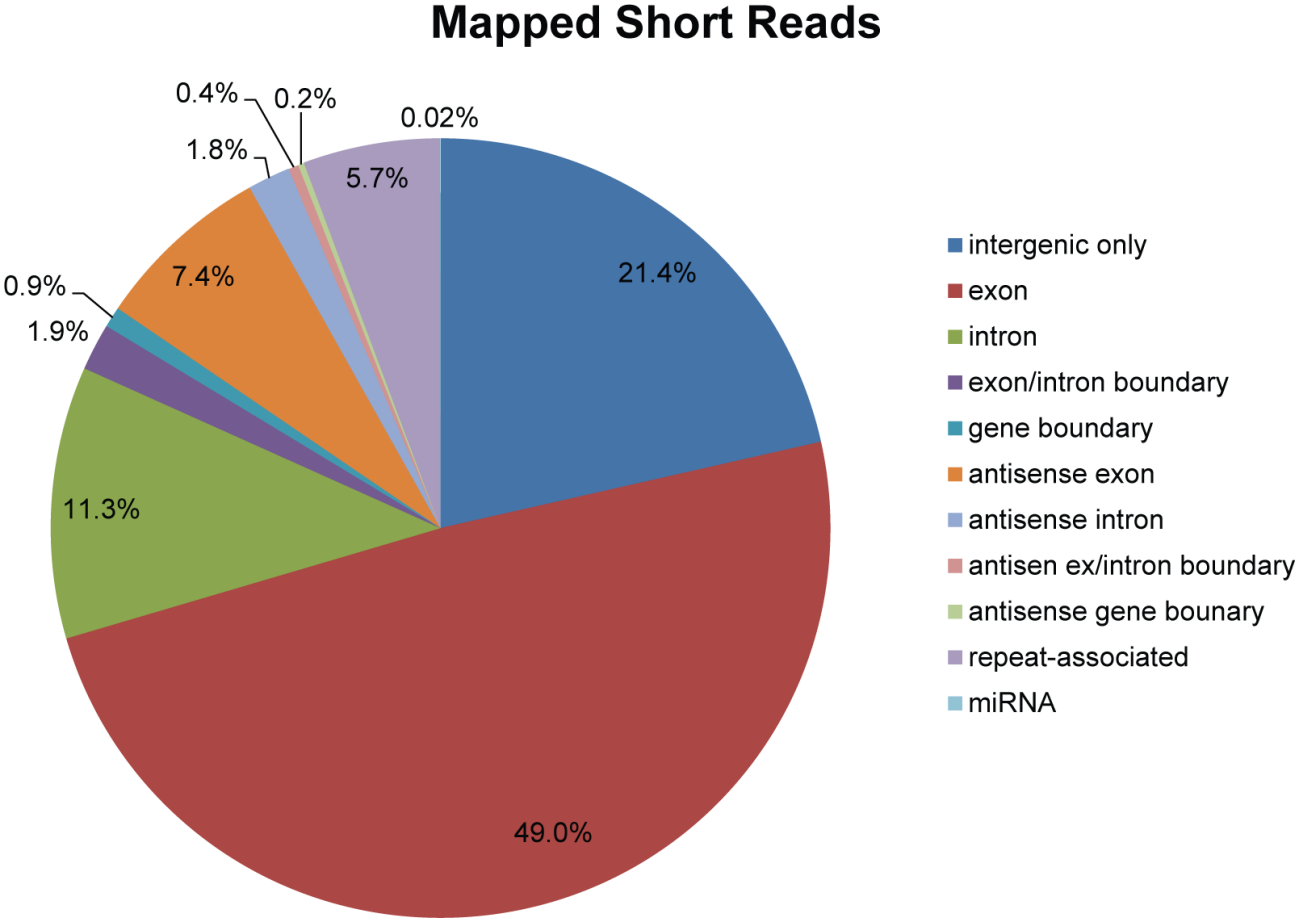
The mapping of small RNAs relative to the annotation of *E*. *huxleyi* genome. If a read is mapped to more than one feature other than intergenic region, the most frequent one is selected.

Natural antisense transcript-derived siRNA (nat-siRNA) are generated from double-strand mRNA transcripts of overlapping gene regions. After removing rasiRNA and miRNA candidates from the smRNA library, the remaining sequences between 18–26 nt were aligned to the genome and those mapping to annotated gene transcripts were examined. Most of reads were transcribed from sense exon, intergenic, and sense intron regions (Fig 5). About 7.4% of the small RNAs were mapped to antisense exons, suggesting a possible natural antisense regulatory mechanism of small RNAs. 89 pairs of genes, moreover, overlap by 25 nt or more. The biogenesis and functional significance of nat-siRNAs in *E*. *huxleyi*, and whether they accumulate in response to biotic and abiotic stress and contribute to resistance to different environmental stress conditions, as they often do in plants [68–71] requires further study.

Piwi-interacting RNAs (piRNAs) are a distinct class of small RNAs that are abundant in most animals, but notably absent in plants and fungi [55]. Amongst the 167,147 unique smRNA reads of 26–30 nt, homology based search methods revealed a single perfect match to a 28 nt human piRNA (hsa_piR_019675), with an e-value = 2e-8. The perfect match sequence is present in the *E*. *huxleyi* genome on multiple scaffolds. This particular piRNA homolog and the presence in the genome of several genes coding for proteins involved in piRNA biogenesis (see below) provides strong evidence for the existence of a piRNA silencing pathway in *E*. *huxleyi*. To predict additional less well conserved piRNAs, a kmer program called piRNApredictor [57] was used and predicted 16,474 novel piRNAs. 5598 out of the 16,474 smRNAs were mapped to the *E*. *huxleyi* genome with at most one match and no gap. The mapped piRNA candidates were predominantly distributed (66.8%) in intergenic regions as compared to the exons (12.2%) or introns (2.7%). Although *E*. *huxleyi* piRNAs have a slight bias for uracil at the 5’ end (35.2%), there is no observable adenosine bias at the 10^th^ position from the 5’ end. This suggests the Ping-Pong or one-way secondary piRNA biogenesis pathway present animals [72] may not exist in *E*. *huxleyi*. In the Ping-Pong cycle, the piRISC complex cleaves transposon transcripts, which silences the transcripts and generates secondary piRNAs.

### Identification of Critical Components of RNA Silencing Pathways

In plants most RNA silencing pathways occur in three distinct phases where there is an initiation, maintenance, and a signal amplification phase. During the initiation phase, different double-stranded RNAs (dsRNAs) including miRNA precursors, viral RNAs, ta-siRNAs, and rasiRNAs, are processed by the ribonuclease DICER (DCL). One strand of the siRNA or miRNA is then incorporated into the multiprotein RNA-induced silencing complex (RISC), which contains argonaute (AGO). AGO with the help of the single-stranded small RNA directs RISC to the mRNA target, where subsequent silencing occurs by either cleavage or blocking target translation. Amplification of silencing signals occurs when a RNA dependent RNA Polymerase (RDR) enzyme synthesizes dsRNA in a template-dependent manner and the dsRNA is then cleaved by DCL into secondary small RNAs to initiate a new round of silencing. A search of the *E*. *huxleyi* genome for key components involved in the biogenesis and processing of siRNAs revealed multiple gene models for DCL, AGO and RDP enzymes, and several possible HEN1 methyltransferase responsible for modifying the 3’-terminal nucleotides of small regulatory RNAs. Other factors potentially involved in piRNA biogenesis were also detected (S10 Table). Most of the RNAi components are found across various *E*. *huxleyi* strains with sequenced genomes (S11 Table).

**Fig 6:**
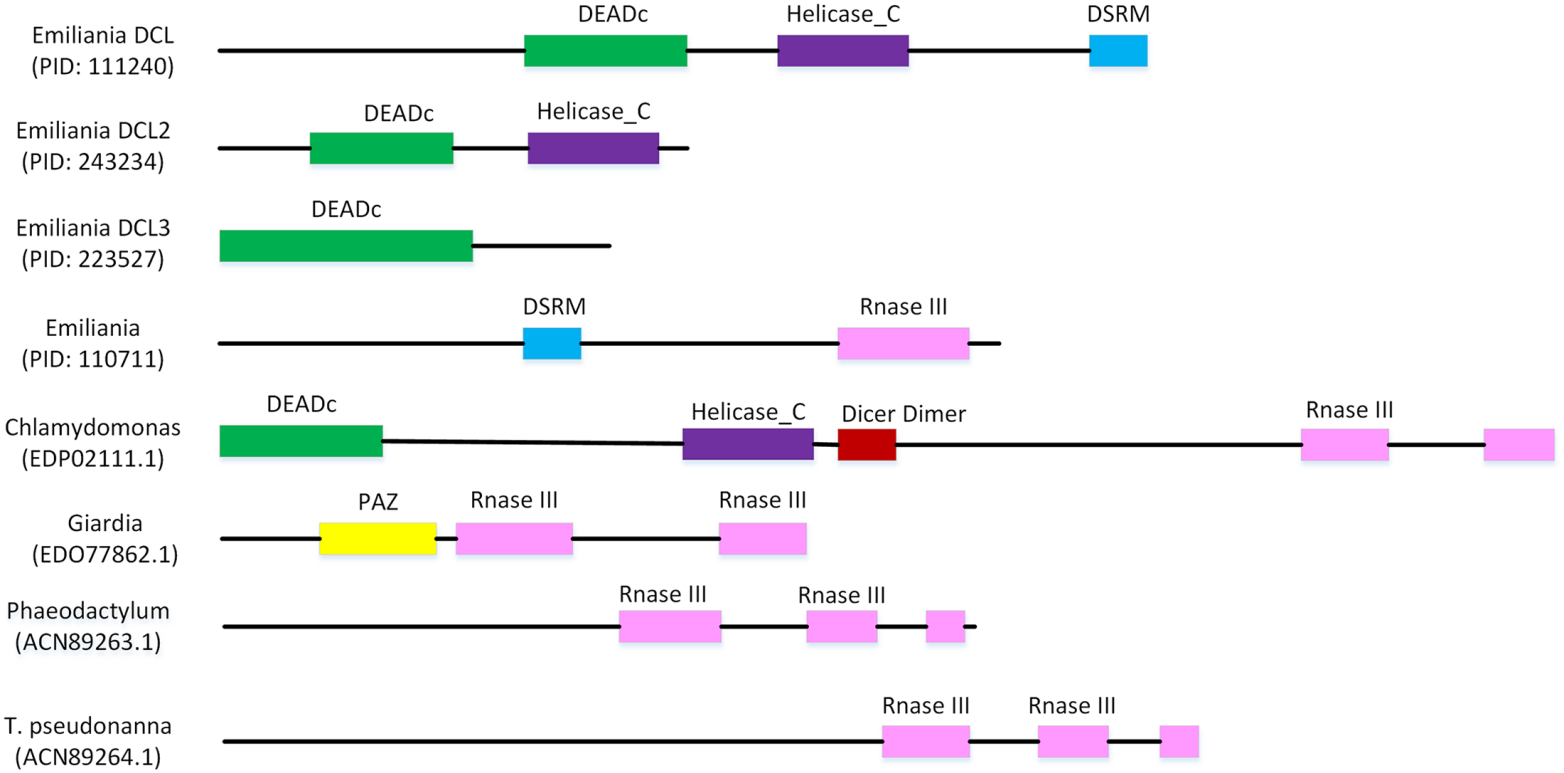
Domain architecture variation in DICER homologs from *E*. *huxleyi* and other simple eukaryotes. The RNase III domain (pink) is responsible for cleaving dsRNA while the helicase domain (purple) is important for unwinding of the dsRNA, the DSRM domain (blue) binds double stranded RNA, and the PAZ domain (yellow) binds to the 3’ end of the target ds RNA. The more complex enzymes in higher eukaryotes typically contain all four domains whereas the ancestral DICERs from unicellular eukaryotes are generally missing one or more domains.

DCL is a double stranded RNA specific enzyme that belongs to the RNase III endonuclease family of proteins and is characterized by DEAD, Helicase-C, dsRBD (double strand RNA binding domain), RNase III, and PAZ (Piwi Argonaute and Zwille) domains. Although four gene models in the genome of *E*. *huxleyi* code for proteins exhibiting significant homology to known DCL enzymes, each lacks one or more of the characteristic domains (Fig 6), making it difficult to recognize any of them as classic DCL enzymes. Non-canonical DCL enzymes, however, are not uncommon amongst algae. The enzyme from *Giardia* possesses only the PAZ domain and two RNAse III domains [73], while the enzyme from *Phaeodactylum tricornutum* contains two RNase III domains and a dsRBD domain, and that of *T*. *pseudonanna* contains only two RNase III domains [74]. Three out of the four *E*. *huxleyi* gene models code for proteins with DEADc, Helicase-C, and/or DSRM domains but without the RNase III domains necessary for RNA binding and cleavage. The fourth model contains DSRM and RNase III domains but not the DEAD or Helicase-C domains. The *E*. *huxleyi* protein is most similar to the DICER-like homolog from *Chlamydomonas* with which it shares 34% amino acid identity and 50% homology across the entire protein sequence (S8 Fig). The level of sequence identity and homology is comparable in the RNase II domain where two out of the four catalytic domains identified by [75] (S9 Fig). Of the four *E*. *huxleyi* gene models, the presence of the RNase III domain together with these conserved catalytic residues makes PID 110711 the most probable DCL. It is difficult without experimental evidence to know which if this is the case and/or whether any of the predicted genes code for DCLs involved in RNAi metabolism. It is important, at the same time, to recognize the growing body of literature describing non-canonical miRNA pathways independent of Dicer [76–78].

**Fig 7:**
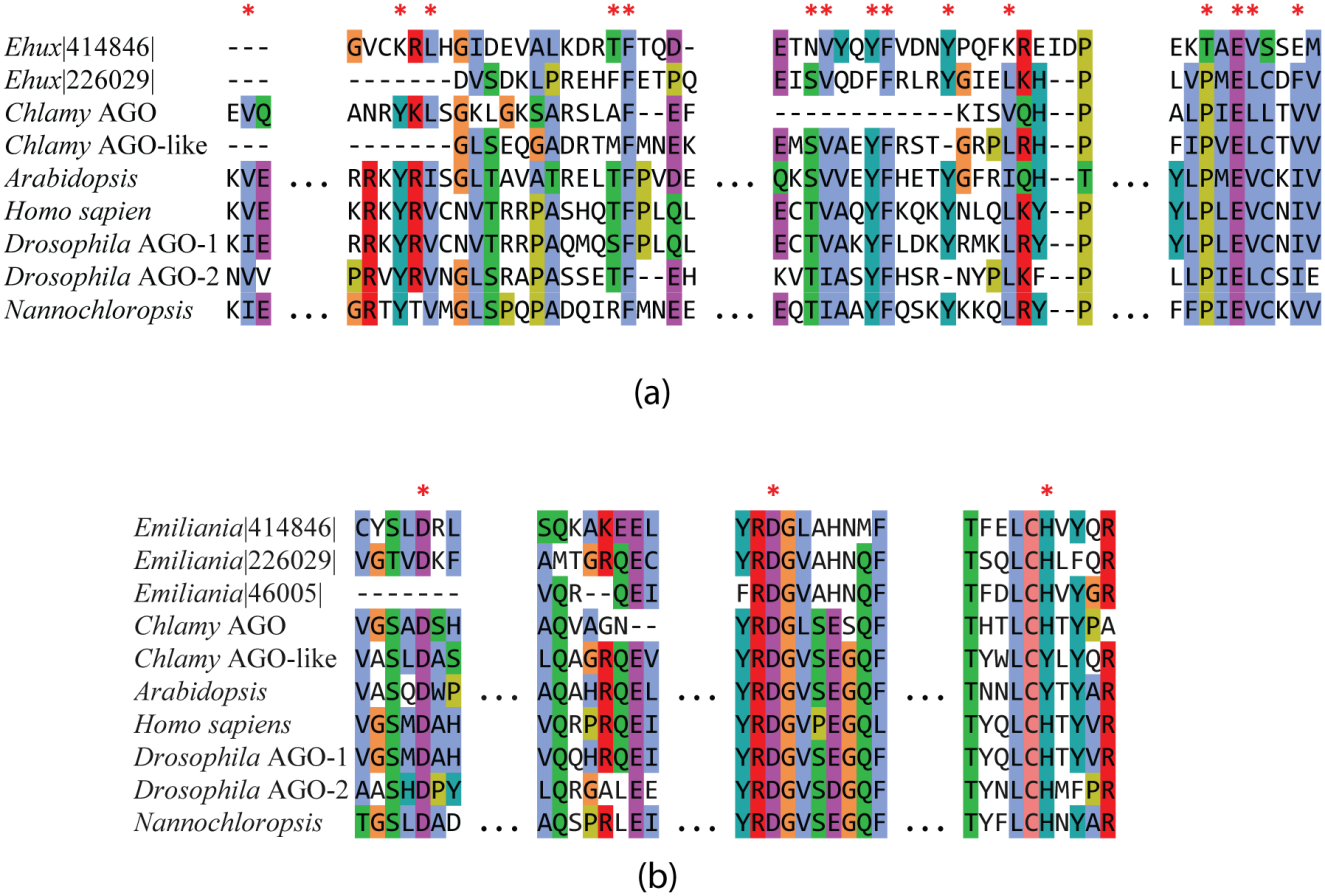
A comparison of argonaute PAZ and PIWI domains from *E*. *huxleyi*, *Arabidopsis*, *Chlamydomonas*, *Homo sapiens*, *Drosophila*, and Nannochloropsis. Several residues in the PAZ domain (A) marked by astericks, are invariant in the *E*. *huxleyi* argonaute (226029) and thought to stabilize the dsRNA-binding region. These include: 1) a subdomain of aromatic residues, 2) a cysteine residue preceded by a proline and a glutamine, and 3) other conserved residues that form a hydrophobic subdomain that interacts with RNA (Firmino et al., 2005). Amino acids in the core motifs of the PIWI domain (B) are also well conserved across the *E*. *huxleyi* homologs, and the catalytic DDH triad marked by asterisks is invariant. Coloring is based on the degree of amino acid homology.

AGO lies at the core of the RNA-induced silencing complex (RISC) and together with single-stranded small RNAs, directs the complex to the mRNA target. The most important functional domains in AGO proteins are a central PAZ and a C-terminal PIWI domain, however, again not all AGO proteins contain both domains. In fact, most prokaryotic and archeal AGOs lack the PAZ domain [79]. As a result of gene duplication and horizontal gene transfer, eukaryotes often possess multiple AGO gene sequences [80]. There are two AGO homologues detected in the *E*. *huxleyi* genome (PID 226029 and 414846) that contain both PAZ and PIWI domains, and share 32% amino acid identity and 50% homology. The catalytic ASP-ASP-His (DDH) triad that plays a critical role during sequence—specific cleavage in the RNAi machinery is invariant [81] in *E*. *huxleyi* AGOs 226029 and 414846; several other residues deemed functionally important in sorting different smRNA species to various AGOs [81,82] are conserved to varying degrees (Fig 7, S10 and S11 Figs). A third protein homologue (PID 46005) contains the PIWI domain but no PAZ domain, and shows significant homology to eukaryotic translation initiation factor 2C2 (EIF2C2). EIF2C2 is a member of the AGO family of proteins that binds to RISC and causes gene silencing by inhibiting translation upon binding to the 7-methylguanosine cap. It is unclear whether the gene in *E*. *huxleyi* codes for a functional EIF2C2/AGO-2 in the absence of the critical PAZ domain required for binding siRNAs. Sequence homology and phylogenetic analyses of the different AGOs in *E*. *huxleyi* suggests they evolved by gene duplication (PIWI-domain containing AGO-like proteins) and convergent evolution (AGO subfamily protein), and most likely not acquired via horizontal gene transfer from prokaryotes or members of the green algal lineage (S12 and S13 Figs). The poor bootstrap values makes the evolutionary history of the AGO-like proteins from *E*. *huxleyi* difficult to discern without additional sequence information. Detailed studies of the structure and biochemistry of both DCL and AGO proteins in *E*. *huxleyi* are required to reveal their precise mechanism of action and function in RNA metabolism.

The *E*. *huxleyi* genome possesses at least one RDR gene (PID 205162) that may be important to siRNA biogenesis by recognizing and using aberrant RNA molecules as templates to amplify RNAi silencing signals. In addition to sharing 35% amino acid sequence identity with the RDR from *Genlisea aurea*, the *E*. *huxelyi* RDR includes the DLDGD motif where the third aspartic acid residue has been shown to be essential for catalytic activity in other organisms (S14 Fig) [83–85]. Although not yet functionally characterized, this putative RDR may play a role in the biogenesis of ra-siRNAs, ta-siRNAs, or nat-siRNAs.

The *in-silico* analysis of key components of the RNAi silencing machinery also revealed the presence of gene models for putative HEN1 methyltransferases that catalyze the transfer of a 2’-O-methyl group to the 3-end of miRNA/miRNA* and siRNA/siRNA* duplexes to increase their stability and target the appropriate argonaute machinery. The 1187 amino acid *E*. *huxleyi* protein shows similarity to the HEN1 methyltransferase from *Arabidopsis* and contains the 8 active site residues essential for making contact with the methyl donor, metal ions, and the RNA methyl acceptor [86] (S15 Fig). Homologs of other factors involved in RNA interference and editing pathways including the putative RNA helicase component of the RISC complex, Armitage (ARMI), and Tudor staphlococccal nuclease (Tudor-SN), another component of the RISC complex associated with piRNA biogenesis, are also detected in *E*. *huxleyi* [87,88] (S16 and S17 Figs, S10 Table) and support the notion that there are multiple functioning RNAi pathways. Although critical active site residues defining the catalytic activity of several of the proteins are largely conserved, the overall domain architecture of some proteins differs considerably from counterparts in other organisms. Unraveling the mechanistic details of RNAi pathways and machinery in *E*. *huxleyi* will now require functionally characterizing these predicted elements; analyzing mutants for each of the homologs and purifying individual of complexes for kinetic studies.

## Conclusion

We constructed a smRNA library from RNA extracted from *E*. *huxleyi* cells at different times during late log and stationary phase growth; during which time nutrients become limiting, cell growth slows, and calcification is enhanced. To identify and characterize miRNAs in *E*. *huxleyi*, smRNAs were sequenced using the Illumina platform. Computational analysis based on the smRNAs identified 18 novel miRNAs in *E*. *huxleyi*. MiRNA* sequences were found for 4 of the novel miRNAs in *E*. *huxleyi*. While providing support for the presence of Dicer processed precursors, these results suggest deeper sequencing under varied conditions is needed to uncover the full complement of miRNAs in *E*. *huxleyi*. Expression profiling performed using stem-loop RT PCR shows transcription of miRNAs varies by more than three orders of magnitude when cells are cultured under laboratory conditions in FSW and are transitioning into stationary phase. Dynamic changes in the expression profiles of several miRNA precursors was also observed when comparing calcifying and non-calcifying cultures of *E*. *huxleyi*. Functional annotation of the predicted target genes of the differentially expressed miRNAs suggests they are involved in regulating a wide variety of cellular processes including metabolism, cell division, autophagy, transcriptional regulation, molecular transport, protein regulation, and cellular localization. This data supports that hypothesis that miRNAs play a significant role in regulating physiological growth and adaptation in *E*. *huxleyi* just as they do in green algae and higher plants. We have identified the core components of the RNAi pathway in *E*. *huxleyi*. There are multiple DCL and AGO family members in *E*. *huxleyi* like there are in other plant and animal species. Determining whether these are functionally distinct proteins that process different types of small RNAs, as they do in *Arabidopsis* will shed further light on the details of RNAi pathways in *E*. *huxleyi*. A closer look at the functionality of the predicted RDR and Hen1 proteins in *E*. *huxleyi* will also provide new insight into the biogenesis and processing of the complex system of siRNAs in this important alga.

## Supporting Information

S1 Fig Growth curves of the non-calcifying CCMP 1516 and the calcifying M217 strains. Cell counts were determined using a hemocytomer and represent average values obtained from three experimental replicates. Error bars represent the standard deviations, some of which are too small to be seen. Although the doubling times of 42.3 and 44.9 hr, and the exponential growth rates of 0.16 and 0.15, respectively for M217 (B, left panel) and CCMP1516 (B, right panel) are similar, the final cell density is higher for CCMP1516. Light micrographs in panel B were taken at 100X.
(PDF)

S2 Fig pH values of the filtered seawater media for growing E. huxleyi strains. (PDF)

S3 Fig miRNA precursors predicted from small RNA reads, in which the predicted miRNAs are colored read and miRNA*’s are colored in purple. (PDF)

S4 Fig Number of targets predicted using (a) plant-like binding characteristics and (b) animal-like binding characteristics. (PDF)

S5 Fig Distributions of level-2 Gene Ontology terms for predicted miRNA target genes with plant-like binding characteristics. (PDF)

S6 Fig Relative expression of a subset of miRNA target genes between calcifying strain 217 and non-calcifying strain 1516. (PDF)

S7 Fig Distribution of 2nd level Gene Ontology terms in Biological Process for ta-siRNA targets. (PDF)

S8 Fig Multiple sequence alignment of the DICER or DICER-like protein from *E*. *huxleyi* (PID: 110711) with the DICER-like protein from *Chlamydomonas reinhardtii* (EDP02111.1) where the sequence homology is 50% and sequence identity is 34%. The RNAse III domain located in different regions in the two proteins is highlighted in blue.
(PDF)

S9 Fig Alignment of the RNAse III domains of the DICER-like proteins from *E*. *huxleyi* and C. reinhardtii share 29% amino acid identity and 45% homology. Two of the four catalytic residues of the RNase III domain identified by [75] are conserved in *E*. *huxleyi* and *C*. *reinhardii* and are highlighted in red.
(PDF)

S10 Fig A comparison of the Paz domain of argonaute from *E*. *huxleyi*, *E*. *huxleyi* argonaute paralogs (226029; 46005; 414846), together with argonaute and argonaute-like homologs from Homo sapiens (NP_036331.1), *Drosophila melanogaster* (NP_730054.1; NP_725341.1), *Arabidopsis thaliana* (NP_849784.1), *Nannochloropsis gaditana* (EWM22342.1) and *Chlamydomonas* (EDO99188.1; EDP01993.1). Highlighted residues, several of which are invariant in the *E*. *huxleyi* argonaute (226029), are thought to stabilize the dsRNA-binding region. These include: 1) a subdomain of aromatic residues, 2) a cysteine residue preceded by a proline and a glutamine, and 3) other conserved residues that form a hydrophobic subdomain that interacts with RNA.
(PDF)

S11 Fig ClustalW multiple sequence alignment of the PIWI domain of the *E*. *huxleyi* argonaute paralogs (226029; 46005; 414846), together with argonaute and argonaute-like homologs from *Homo sapiens* (NP_036331.1), *Drosophila melanogaster* (NP_730054.1; NP_725341.1), *Arabidopsis thaliana* (NP_849784.1), *Nannochloropsis gaditana* (EWM22342.1) and *Chlamydomonas* (EDO99188.1; EDP01993.1). Invariant residues are indicated by asterisks; highly residues by semicolon; and homologous residues by a period. Catalytic residues of the PIWI domain are highlighted in red.
(PDF)

S12 Fig Maximum Likelihood tree based on eukaryotic AGO proteins from the PIWI and AGO subfamilies. Alignments of protein sequences were made with Muscle and the tree built with PhyML3.0. The sequences used in this alignment are *Thalassiosira pseudonana* (Tp) Tp_Ago (JGI ProtID1029); *Phaeodactylum tricornutum* (Pt) Pt_Ago (JGI ProtID47611*); Drosophila melanogaster* (Dm) Aubergine (CAA64320), PIWI (NP_476875), Ago1 (NP_725341), Ago 2 (NP_730054), Ago3 (ABO27430); *Homo sapiens* (Hs) Hili (NP_060538), Hiwi (NP_004755), Hiwi 2 (NP_689644), Hiwi 3 (NP_001008496), Ago1 (NP_036331), Ago2 (NP_036286), Ago3 (NP_079128), Ago4 (NP_060099); *Chlamydomonas reinhardtii* (Cr) (XP_001694841.1); M*icromonas sp*. RCC299 (MRCC299) (ACO60725.1); *Shizosaccharomyces pombe* (Sp) Ago (NP_587782); *Ectocarpus siliculosus* (Es) Ago1 (CBJ30598.1); *Emiliania huxleyi* (Eh) (JGI ProtID226029); *Caenorhabiditis elegans* (Ce) Alg1 (NP_510322), Alg2 (NP_871992); *Arabidopsis thaliana* (At) Ago1 (NP_849784), Ago2 (NP_174413), Ago3 (NP_174414), Ago4 (NP_565633), Ago5 (At2g27880), Ago6 (At2g32940), Ago7 (NP_177103), Ago8 (NP_197602), Ago9 (CAD66636), Ago10 (NP_199194). Accession numbers are for GenBank and genome portals (Tp, Pt, Eh) at the Joint Genome Institute (JGI), US.
(PDF)

S13 Fig Maximum Likelihood tree based on eukaryotic (EUK) and prokaryotic (PRK) PIWI domain-containing AGO-like proteins. Alignments of protein sequences were made with Muscle and the tree built with PhyML3.0. Gene identifiers are given in the tree and are either for GeneBank or the Emiliania huxleyi genome portal at the Joint Genome Institute (JGI), US.
(PDF)

S14 Fig Multiple sequence alignment of the catalytic domain of *E*. *huxleyi* RdP and its homologs from C. elegans RRF-1, S. pombe Rdp1, A thaliana Rdp, N. gruberi RdP, and L. corymbifera RdP. Invariant residues are marked with an asterisk; colon, conserved residues. The aspartic acid residue that is essential for RNA dependent RNA polymerase activity is highlighted in red.
(PDF)

S15 Fig Clustalw (2.1) multiple sequence alignment of HEN1 methyltransferase of *Arabidopsis thaliania* (AT4G20910) and E. *Huxley*i (454426). Invariant residues are indicated by asterisks while highly and moderately homologous amino acid residues are indicated by “:” and “.”, respectively. Eight essential active site residues that contact the methyl donor, metal ions, and the RNA methyl acceptor are highlighted in red.
(PDF)

S16 Fig S16 Multiple sequence analysis of the Armitage SDE3 protein from, E. huxleyi (247007;119956;436918), *Arabidopsis thaliana* (AEE27843.1) and *Drosophila melanogaster* (AAT12000.1). Considerable sequence divergence is noted in the N-terminus. The C-terminus is more highly conserved and contains the ATPase and the MOV-10 helicase domains. While the homologs from *E*. *huxleyi* share between 30–40% amino acid identity,they share 20–30% identity with homologs from Arabidopsis and Drosophila. Conserved helicase motifs are highlighted in red.
(PDF)

S17 Fig CLUSTAL (2.1) Multiple sequence alignment for the Tudor staphylococcal nuclease (TUDOR-SN) from *E*. *huxleyi*, *Populus trichocarpa* (EEF06439.1), *Apis mellifera* (XP_624638.3), *Arabidopsis thaliana* isozymes 1(NP_001154697.2) and 2 (NP_200986.1, and *Drosophila melangastor* isozymes A (NP_612021.1) and B (NP_001261195.1). Key residues in the TUDOR domain include highly conserved Arginine and Aspartate residues in green; aromatic cage residues in red, responsible for complexing di-methylated guanine groups; Asparagine in purple, involved in the binding and interaction with PIWI proteins by means of the symmetric di-methylation of distinct arginine (sDMA) residues; and a strongly conserved Glycine in in blue.
(PDF)

S1 File Predicted target genes of miRNA candidates, annotated with blastp results against the UniProt and NCBI NR databases, KOG and KEGG definitions from JGI genome portal, and domains from interproScan. (XLSX)

S2 File Predicted targets of ta-siRNA candidates, annotated with blastp results against the UniProt and NCBI NR databases, KOG and KEGG definitions from JGI genome portal, and domains from interproScan. (XLSX)

S1 Table Homology search results for known miRNAs. (DOC)

S2 Table Sequences and Loci of miRNA candidate precursors (DOC)

S3 Table Blastn homology search results comparing miRNA precursors to the non-coding sequences in the European Nucleotide Archive (ENA) and NCBI nucleotide collection (NR/NT). (DOC)

S4 Table MiRNA precursors in other strains of *Emiliania*. The table shows the identity of alignment of the precursors in the other strains (all alignments have e-value < 1e-10).
(DOC)

S5 Table Mature miRNA in three other strains of *E*. *huxleyi*. The table shows the percentage of matched bases of the miRNA candidates to the strain genomes.
(DOC)

S6 Table Predicted miRNA target genes amongst the genes differentially expressed between life-cycles, up-regulated in haploid (1N) and up-regulated in dipoild (2N) respectively [37]. (DOC)

S7 Table Characteristics of stem-loop quantitative real time PCR miRNA primers and their corresponding amplification products. (DOC)

S8 Table Real-Time PCR primer sets and features of the amplification products for a select set of mir target genes. (DOC)

S9 Table The predicted groups of Trans-acting siRNAs (ta-siRNAs) candidates. (DOC)

S10 Table Possible candidates for *E*. *huxleyi* RNAi components. (DOC)

S11 Table Analysis of candidate *E*. *huxleyi* RNAi components in other strains. The table shows the e-value of blastn search of candidate RNAi genes against the genomes of three other *E*. *huxleyi* strains.
(DOC)

## References

[1] Holligan PM, Viollier M, Harbour DS, Camus P, Champagne-Philippe M. Satellite and ship studies of coccolithophore production along a continental shelf edge. Nature. 1983;304: 339–342.

[2] Smith S V. Parsing the oceanic calcium carbonate cycle: a net atmospheric carbon dioxide source, or a sink? Anderson MR, editor. Association for the Sciences of Limnology and Oceanography; 2013.

[3] Armstrong RA, Lee C, Hedges JI, Honjo S, Wakeham SG. A new, mechanistic model for organic carbon fluxes in the ocean based on the quantitative association of POC with ballast minerals. Deep Sea Res Part II Top Stud Oceanogr. 2001;49: 219–236.

[4] Klaas C, Archer DE. Association of sinking organic matter with various types of mineral ballast in the deep sea: Implications for the rain ratio. Global Biogeochem Cycles. 2002;16: 63–1–63–14.

[5] Marlowe IT, Green JC, Neal AC, Brassell SC, Eglinton G, Course PA. Long chain (n -C 37 –C 39) alkenones in the Prymnesiophyceae. Distribution of alkenones and other lipids and their taxonomic significance. Br Phycol J. Taylor & Francis; 1984;19: 203–216.

[6] Marlowe I, Brassell S, Eglinton G, Green J. Long-chain alkenones and alkyl alkenoates and the fossil coccolith record of marine sediments. Chem Geol. 1990;88: 349–375.

[7] Beaufort L, Probert I, de Garidel-Thoron T, Bendif EM, Ruiz-Pino D, Metzl N, et al Sensitivity of coccolithophores to carbonate chemistry and ocean acidification. Nature. Nature Publishing Group, a division of Macmillan Publishers Limited. All Rights Reserved.; 2011;476: 80–3. 10.1038/nature10295 21814280

[8] Paasche E. A review of the coccolithophorid Emiliania huxleyi (Prymnesiophyceae), with particular reference to growth, coccolith formation, and calcification-photosynthesis interactions. Phycologia. The International Phycological Society Phycologia Business Office, Allen Press, 810 East 10th Street, P.O. Box 1897, Lawrence, KS 66044–8897; 2001;40: 503–529.

[9] Read BA, Kegel J, Klute MJ, Kuo A, Lefebvre SC, Maumus F, et al Pan genome of the phytoplankton Emiliania underpins its global distribution. Nature. Nature Publishing Group; 2013;499: 209–213. 10.1038/nature12221 23760476

[10] Bartel DP. MicroRNAs: Genomics, Biogenesis, Mechanism, and Function. Cell. 2004;116: 281–297. 14744438 10.1016/s0092-8674(04)00045-5

[11] Farazi TA, Juranek SA, Tuschl T. The growing catalog of small RNAs and their association with distinct Argonaute/Piwi family members. Development. 2008;135: 1201–1214. 10.1242/dev.005629 18287206

[12] Vaucheret H. Post-transcriptional small RNA pathways in plants: mechanisms and regulations. Genes Dev. 2006;20: 759–71. 16600909 10.1101/gad.1410506

[13] Zaratiegui M, Irvine D V, Martienssen RA. Noncoding RNAs and gene silencing. Cell. 2007;128: 763–76. 17320512 10.1016/j.cell.2007.02.016

[14] Bumcrot D, Manoharan M, Koteliansky V, Sah DWY. RNAi therapeutics: a potential new class of pharmaceutical drugs. Nat Chem Biol. 2006;2: 711–9. 17108989 10.1038/nchembio839 PMC7097247

[15] Echeverri CJ, Perrimon N. High-throughput RNAi screening in cultured cells: a user’s guide. Nat Rev Genet. 2006;7: 373–84. 16607398 10.1038/nrg1836

[16] Lim LP, Lau NC, Weinstein EG, Abdelhakim A, Yekta S, Rhoades MW, et al The microRNAs of Caenorhabditis elegans. Genes Dev. 2003;17: 991–1008. 12672692 10.1101/gad.1074403 PMC196042

[17] Bartel DP. MicroRNAs: target recognition and regulatory functions. Cell. 2009;136: 215–33. 10.1016/j.cell.2009.01.002 19167326 PMC3794896

[18] Jones-Rhoades MW, Bartel DP, Bartel B. MicroRNAS and their regulatory roles in plants. Annu Rev Plant Biol. 2006;57: 19–53. 16669754 10.1146/annurev.arplant.57.032905.105218

[19] Kutter C, Svoboda P. miRNA, siRNA, piRNA: Knowns of the unknown. RNA Biol. 2008;5: 181–188. 19182524 10.4161/rna.7227

[20] Rajagopalan R, Vaucheret H, Trejo J, Bartel DP. A diverse and evolutionarily fluid set of microRNAs in Arabidopsis thaliana. Genes Dev. 2006;20: 3407–3425. 17182867 10.1101/gad.1476406 PMC1698448

[21] Thomassen GOS, Røsok Ø, Rognes T. Computational Prediction of MicroRNAs Encoded in Viral and Other Genomes. J Biomed Biotechnol. 2006;2006: 1–11. 10.1155/JBB/2006/95270 PMC1559940 17057374

[22] Weaver DB, Anzola JM, Evans JD, Reid JG, Reese JT, Childs KL, et al Computational and transcriptional evidence for microRNAs in the honey bee genome. Genome Biol. 2007;8: R97 17543122 10.1186/gb-2007-8-6-r97 PMC2394756

[23] Yao Y, Guo G, Ni Z, Sunkar R, Du J, Zhu J-K, et al Cloning and characterization of microRNAs from wheat (Triticum aestivum L.). Genome Biol. 2007;8: R96 17543110 10.1186/gb-2007-8-6-r96 PMC2394755

[24] Molnár A, Schwach F, Studholme DJ, Thuenemann EC, Baulcombe DC. miRNAs control gene expression in the single-cell alga Chlamydomonas reinhardtii. Nature. 2007;447: 1126–1129. 17538623 10.1038/nature05903

[25] Zhao T, Li G, Mi S, Li S, Hannon GJ, Wang X-J, et al A complex system of small RNAs in the unicellular green alga Chlamydomonas reinhardtii. Genes Dev. 2007;21: 1190–1203. 17470535 10.1101/gad.1543507 PMC1865491

[26] Norden-Krichmar TM, Allen AE, Gaasterland T, Hildebrand M. Characterization of the small RNA transcriptome of the diatom, Thalassiosira pseudonana. Friedberg I, editor. PLoS One. Public Library of Science; 2011;6: e22870. 10.1371/journal.pone.0022870 PMC3155517 21857960

[27] Cock JM, Sterck L, Rouzé P, Scornet D, Allen AE, Amoutzias G, et al The Ectocarpus genome and the independent evolution of multicellularity in brown algae. Nature. Nature Publishing Group, a division of Macmillan Publishers Limited. All Rights Reserved.; 2010;465: 617–21. 10.1038/nature09016 20520714

[28] Billoud B, Nehr Z, Le Bail A, Charrier B. Computational prediction and experimental validation of microRNAs in the brown alga Ectocarpus siliculosus. Nucleic Acids Res. 2014;42: 417–29. 10.1093/nar/gkt856 24078085 PMC3874173

[29] Liang C, Zhang X, Zou J, Xu D, Su F, Ye N. Identification of miRNA from Porphyra yezoensis by high-throughput sequencing and bioinformatics analysis. Goldstien SJ, editor. PLoS One. Public Library of Science; 2010;5: e10698. 10.1371/journal.pone.0010698 PMC2873431 20502668

[30] Peters L, Meister G. Argonaute Proteins: Mediators of RNA Silencing. Mol Cell. 2007;26: 611–623. 17560368 10.1016/j.molcel.2007.05.001

[31] Pillai RS, Bhattacharyya SN, Filipowicz W. Repression of protein synthesis by miRNAs: how many mechanisms? Trends Cell Biol. 2007;17: 118–26. 17197185 10.1016/j.tcb.2006.12.007

[32] Mardis ER. Next-generation DNA sequencing methods. Annu Rev Genomics Hum Genet. Annual Reviews; 2008;9: 387–402. 10.1146/annurev.genom.9.081307.164359 18576944

[33] Griffiths-Jones S, Grocock RJ, van Dongen S, Bateman A, Enright AJ. miRBase: microRNA sequences, targets and gene nomenclature. Nucl Acids Res. 2006;34: D140–144. 16381832 10.1093/nar/gkj112 PMC1347474

[34] Guillard RR, Ryther JH. Studies of marine planktonic diatoms. I. Cyclotella nana Hustedt, and Detonula confervacea (cleve) Gran. Can J Microbiol. 1962;8: 229–39. 13902807 10.1139/m62-029

[35] Taylor AR, Chrachri A, Wheeler G, Goddard H, Brownlee C. A voltage-gated H+ channel underlying pH homeostasis in calcifying coccolithophores. PLoS Biol. Public Library of Science; 2011;9: e1001085 10.1371/journal.pbio.1001085 PMC3119654 21713028

[36] Mackinder L, Wheeler G, Schroeder D, von Dassow P, Riebesell U, Brownlee C. Expression of biomineralization-related ion transport genes in Emiliania huxleyi. Environ Microbiol. 2011;13: 3250–65. 10.1111/j.1462-2920.2011.02561.x 21902794

[37] Rokitta SD, de Nooijer LJ, Trimborn S, de Vargas C, Rost B, John U. Transcriptome analyses reveal differential gene expression patterns between the life-cycle stages of Emiliania Huxleyi (Haptophyta) and reflect specialization to different ecological niches. J Phycol. 2011;47: 829–838. 10.1111/j.1529-8817.2011.01014.x 27020019

[38] Strommer J, Gregerson R, Vayda M, Glick BR, Thompson JE. Isolation and characterization of plant mRNA. CRC Press; 1993; 49–65.

[39] Chan PP, Lowe TM. GtRNAdb: a database of transfer RNA genes detected in genomic sequence. Nucleic Acids Res. 2009;37: D93–7. 10.1093/nar/gkn787 18984615 PMC2686519

[40] Pruesse E, Quast C, Knittel K, Fuchs BM, Ludwig W, Peplies J, et al SILVA: a comprehensive online resource for quality checked and aligned ribosomal RNA sequence data compatible with ARB. Nucleic Acids Res. 2007;35: 7188–96. 17947321 10.1093/nar/gkm864 PMC2175337

[41] Hofacker IL, Fontana W, Stadler PF, Bonhoeffer LS, Tacker M, Schuster P. Fast folding and comparison of RNA secondary structures. Chem Mon. Springer Wien; 1994;125: 167–188.

[42] Jones-rhoades MW, Bartel DP. Computational Identification of Plant MicroRNAs and Their Targets, Including a Stress-Induced miRNA. Mol Cell. 2004;14: 787–799. 15200956 10.1016/j.molcel.2004.05.027

[43] Meyers BC, Axtell MJ, Bartel B, Bartel DP, Baulcombe D, Bowman JL, et al Criteria for annotation of plant MicroRNAs. Plant Cell. 2008;20: 3186–90. 10.1105/tpc.108.064311 19074682 PMC2630443

[44] Stocks MB, Moxon S, Mapleson D, Woolfenden HC, Mohorianu I, Folkes L, et al The UEA sRNA workbench: a suite of tools for analysing and visualizing next generation sequencing microRNA and small RNA datasets. Bioinformatics. 2012;28: 2059–61. 10.1093/bioinformatics/bts311 22628521 PMC3400958

[45] Enright AJ, John B, Gaul U, Tuschl T, Sander C, Marks DS. MicroRNA targets in Drosophila. Genome Biol. 2003;5: R1 14709173 10.1186/gb-2003-5-1-r1 PMC395733

[46] Dai X, Zhao PX. psRNATarget: a plant small RNA target analysis server. Nucleic Acids Res. 2011;39: W155–9. 10.1093/nar/gkr319 21622958 PMC3125753

[47] Zhang Y. miRU: an automated plant miRNA target prediction server. Nucleic Acids Res. 2005;33: W701–4. 15980567 10.1093/nar/gki383 PMC1160144

[48] De Bodt C, Harlay J, Chou L. Biocalcification by <I>Emiliania huxleyi</I> in batch culture experiments. Mineral Mag. Mineralogical Society of Great Britain and Ireland; 2008;72: 251–256. 10.1180/minmag.2008.072.1.251

[49] Shiraiwa Y. Physiological regulation of carbon fixation in the photosynthesis and calcification of coccolithophorids. Comp Biochem Physiol B Biochem Mol Biol. 2003;136: 775–83. 14662302 10.1016/s1096-4959(03)00221-5

[50] Pan Y-Z, Morris ME, Yu A-M. MicroRNA-328 negatively regulates the expression of breast cancer resistance protein (BCRP/ABCG2) in human cancer cells. Mol Pharmacol. 2009;75: 1374–9. 10.1124/mol.108.054163 19270061 PMC2684886

[51] Kramer MF. Stem-loop RT-qPCR for miRNAs. Curr Protoc Mol Biol. 2011;Chapter 15: Unit 15.10. 10.1002/0471142727.mb1510s95 PMC3152947 21732315

[52] Rutledge RG, Côté C. Mathematics of quantitative kinetic PCR and the application of standard curves. Nucleic Acids Res. 2003;31: e93 12907745 10.1093/nar/gng093 PMC169985

[53] Chen H-M, Li Y-H, Wu S-H. Bioinformatic prediction and experimental validation of a microRNA-directed tandem trans-acting siRNA cascade in Arabidopsis. Proc Natl Acad Sci U S A. 2007;104: 3318–23. 17360645 10.1073/pnas.0611119104 PMC1805617

[54] Moxon S, Schwach F, Dalmay T, Maclean D, Studholme DJ, Moulton V. A toolkit for analysing large-scale plant small RNA datasets. Bioinformatics. 2008;24: 2252–3. 10.1093/bioinformatics/btn428 18713789

[55] Ishizu H, Siomi H, Siomi MC. Biology of PIWI-interacting RNAs: new insights into biogenesis and function inside and outside of germlines. Genes Dev. 2012;26: 2361–73. 10.1101/gad.203786.112 23124062 PMC3489994

[56] Sai Lakshmi S, Agrawal S. piRNABank: a web resource on classified and clustered Piwi-interacting RNAs. Nucleic Acids Res. 2008;36: D173–7. 17881367 10.1093/nar/gkm696 PMC2238943

[57] Zhang Y, Wang X, Kang L. A k-mer scheme to predict piRNAs and characterize locust piRNAs. Bioinformatics. 2011;27: 771–6. 10.1093/bioinformatics/btr016 21224287 PMC3051322

[58] Bonnet E, Van de Peer Y, Rouzé P. The small RNA world of plants. New Phytol. 2006;171: 451–68. 16866953 10.1111/j.1469-8137.2006.01806.x

[59] von Dassow P, John U, Ogata H, Probert I, Bendif EM, Kegel JU, et al Life-cycle modification in open oceans accounts for genome variability in a cosmopolitan phytoplankton. ISME J. International Society for Microbial Ecology; 2015;9: 1365–77. 10.1038/ismej.2014.221 PMC4438323 25461969

[60] Kegel JU, John U, Valentin K, Frickenhaus S. Genome variations associated with viral susceptibility and calcification in Emiliania huxleyi. PLoS One. Public Library of Science; 2013;8: e80684 10.1371/journal.pone.0080684 PMC3834299 24260453

[61] Fernandez-Valverde SL, Taft RJ, Mattick JS. Dynamic isomiR regulation in Drosophila development. RNA. 2010;16: 1881–8. 10.1261/rna.2379610 20805289 PMC2941097

[62] Yi S, Gao Z-X, Zhao H, Zeng C, Luo W, Chen B, et al Identification and characterization of microRNAs involved in growth of blunt snout bream (Megalobrama amblycephala) by Solexa sequencing. BMC Genomics. 2013;14: 754 10.1186/1471-2164-14-754 24188211 PMC3827868

[63] Yan Z, Fang Z, Ma Z, Deng J, Li S, Xie L, et al Biomineralization: functions of calmodulin-like protein in the shell formation of pearl oyster. Biochim Biophys Acta. 2007;1770: 1338–44. 10.1016/j.bbagen.2007.06.018 17692465

[64] Marin F, Corstjens P, de Gaulejac B, de Vrind-De Jong E, Westbroek P. Mucins and molluscan calcification. Molecular characterization of mucoperlin, a novel mucin-like protein from the nacreous shell layer of the fan mussel Pinna nobilis (Bivalvia, pteriomorphia). J Biol Chem. 2000;275: 20667–75. 10.1074/jbc.M003006200 10770949

[65] Boskey AL. Biomineralization: an overview. Connect Tissue Res. 2003;44 Suppl 1: 5–9. 12952166

[66] Midura RJ, Hascall VC. Bone sialoprotein—a mucin in disguise? Glycobiology. 1996;6: 677–81. 8953277 10.1093/glycob/6.7.677

[67] Smit A, Hubley R, Green P. RepeatMasker Open-3.0 [Internet]. 2010. Available: http://www.repeatmasker.org

[68] Zhang X, Xia J, Lii YE, Barrera-Figueroa BE, Zhou X, Gao S, et al Genome-wide analysis of plant nat-siRNAs reveals insights into their distribution, biogenesis and function. Genome Biol. 2012;13: R20 10.1186/gb-2012-13-3-r20 22439910 PMC3439971

[69] Jin H, Vacic V, Girke T, Lonardi S, Zhu J-K. Small RNAs and the regulation of cis-natural antisense transcripts in Arabidopsis. BMC Mol Biol. 2008;9: 6 10.1186/1471-2199-9-6 18194570 PMC2262095

[70] Katiyar-Agarwal S, Morgan R, Dahlbeck D, Borsani O, Villegas A, Zhu J-K, et al A pathogen-inducible endogenous siRNA in plant immunity. Proc Natl Acad Sci U S A. 2006;103: 18002–7. 17071740 10.1073/pnas.0608258103 PMC1693862

[71] Borsani O, Zhu J, Verslues PE, Sunkar R, Zhu J-K. Endogenous siRNAs derived from a pair of natural cis-antisense transcripts regulate salt tolerance in Arabidopsis. Cell. 2005;123: 1279–91. 16377568 10.1016/j.cell.2005.11.035 PMC3137516

[72] Grimson A, Srivastava M, Fahey B, Woodcroft BJ, Chiang HR, King N, et al Early origins and evolution of microRNAs and Piwi-interacting RNAs in animals. Nature. Macmillan Publishers Limited. All rights reserved; 2008;455: 1193–7. 10.1038/nature07415 PMC3837422 18830242

[73] Macrae IJ, Zhou K, Li F, Repic A, Brooks AN, Cande WZ, et al Structural basis for double-stranded RNA processing by Dicer. Science. 2006;311: 195–8. 16410517 10.1126/science.1121638

[74] De Riso V, Raniello R, Maumus F, Rogato A, Bowler C, Falciatore A. Gene silencing in the marine diatom Phaeodactylum tricornutum. Nucleic Acids Res. 2009;37: e96 10.1093/nar/gkp448 19487243 PMC2724275

[75] Gao Z, Wang M, Blair D, Zheng Y, Dou Y. Phylogenetic analysis of the endoribonuclease Dicer family. PLoS One. 2014;9: 1–7. 10.1371/journal.pone.0095350 PMC3991619 24748168

[76] Maurin T, Cazalla D, Yang S, Bortolamiol-Becet D, Lai EC. RNase III-independent microRNA biogenesis in mammalian cells. RNA. 2012;18: 2166–73. 10.1261/rna.036194.112 23097423 PMC3504669

[77] Luhur A, Chawla G, Wu Y-C, Li J, Sokol NS. Drosha-independent DGCR8/Pasha pathway regulates neuronal morphogenesis. Proc Natl Acad Sci U S A. 2014;111: 1421–6. 10.1073/pnas.1318445111 24474768 PMC3910640

[78] Ha M, Kim VN. Regulation of microRNA biogenesis. Nat Rev Mol Cell Biol. Nature Publishing Group, a division of Macmillan Publishers Limited. All Rights Reserved.; 2014;15: 509–524. 10.1038/nrm3838 25027649

[79] Makarova KS, Wolf YI, van der Oost J, Koonin E V. Prokaryotic homologs of Argonaute proteins are predicted to function as key components of a novel system of defense against mobile genetic elements. Biol Direct. 2009;4: 29 19706170 10.1186/1745-6150-4-29 PMC2743648

[80] Vaucheret H. Plant ARGONAUTES. Trends Plant Sci. 2008;13: 350–8. 10.1016/j.tplants.2008.04.007 18508405

[81] Singh RK, Gase K, Baldwin IT, Pandey SP. Molecular evolution and diversification of the Argonaute family of proteins in plants. BMC Plant Biol. 2015;15: 23 10.1186/s12870-014-0364-6 25626325 PMC4318128

[82] Firmino AAP, de A Fonseca FC, de Macedo LLP, Coelho RR, Antonino de Souza JD, Togawa RC, et al Transcriptome analysis in cotton boll weevil (Anthonomus grandis) and RNA interference in insect pests. PLoS One. Public Library of Science; 2013;8: e85079 10.1371/journal.pone.0085079 PMC3874031 24386449

[83] Curaba J, Chen X. Biochemical activities of Arabidopsis RNA-dependent RNA polymerase 6. J Biol Chem. 2008;283: 3059–66. 18063577 10.1074/jbc.M708983200 PMC2629599

[84] Sugiyama T, Cam H, Verdel A, Moazed D, Grewal SIS. RNA-dependent RNA polymerase is an essential component of a self-enforcing loop coupling heterochromatin assembly to siRNA production. Proc Natl Acad Sci U S A. 2005;102: 152–7. 15615848 10.1073/pnas.0407641102 PMC544066

[85] Marker S, Le Mouël A, Meyer E, Simon M. Distinct RNA-dependent RNA polymerases are required for RNAi triggered by double-stranded RNA versus truncated transgenes in Paramecium tetraurelia. Nucleic Acids Res. 2010;38: 4092–107. 10.1093/nar/gkq131 20200046 PMC2896523

[86] Jain R, Shuman S. Active site mapping and substrate specificity of bacterial Hen1, a manganese-dependent 3’ terminal RNA ribose 2'O-methyltransferase. RNA. 2011;17: 429–38. 10.1261/rna.2500711 21205839 PMC3039143

[87] Goodier JL, Cheung LE, Kazazian HH. MOV10 RNA helicase is a potent inhibitor of retrotransposition in cells. PLoS Genet. Public Library of Science; 2012;8: e1002941 10.1371/journal.pgen.1002941 PMC3475670 23093941

[88] Handler D, Olivieri D, Novatchkova M, Gruber FS, Meixner K, Mechtler K, et al A systematic analysis of Drosophila TUDOR domain-containing proteins identifies Vreteno and the Tdrd12 family as essential primary piRNA pathway factors. EMBO J. 2011;30: 3977–93. 10.1038/emboj.2011.308 21863019 PMC3209783

