# Supplementary material for: Characterization of the Small RNA Transcriptome of the Marine Coccolithophorid, Emiliania huxleyi
Source: PLoS One. 2016 Apr 21;11(4):e0154279. doi: 10.1371/journal.pone.0154279 (PMC4839659; doi:10.1371/journal.pone.0154279)
Supplement: S1 Fig — Cell counts were determined using a hemocytomer and represent average values obtained from three experimental replicates. Error bars represent the standard deviations, some of which are too small to be seen. Although the doubling times of 42.3 and 44.9 hr, and the exponential growth rates of 0.16 and 0.15, respectively for M217 (B, left panel) and CCMP1516 (B, right panel) are similar, the final cell density is higher for CCMP1516. Light micrographs in panel B were taken at 100X. (PDF) [file pone.0154279.s001.pdf]

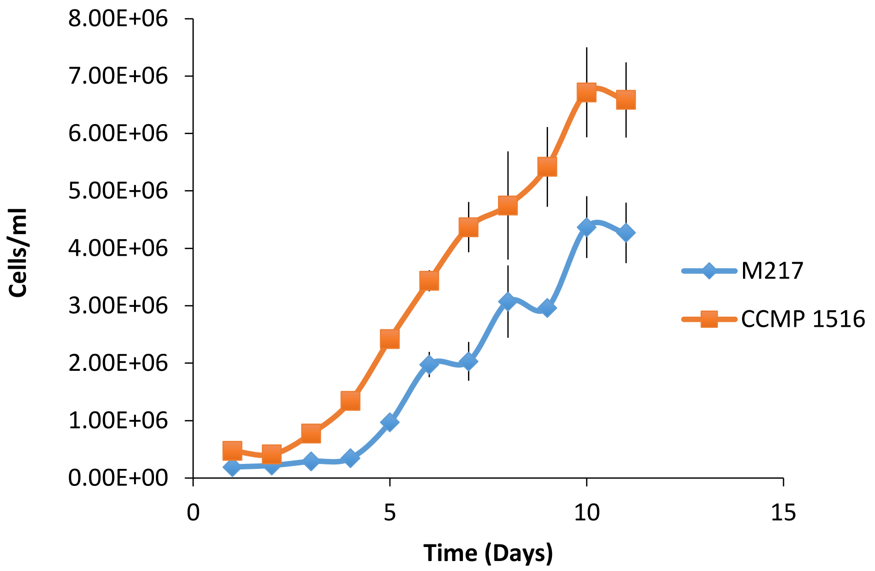

(A)

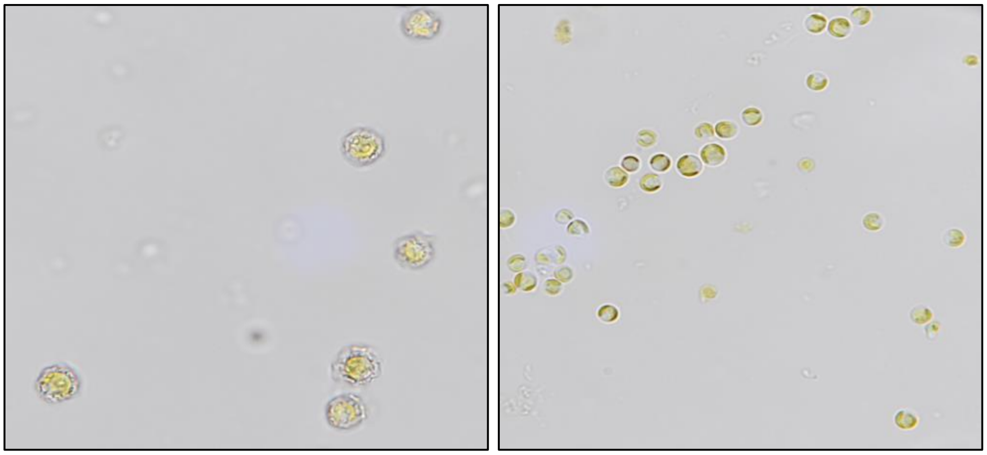

(B)

S1 Fig. Growth curves of the non-calcifying CCMP 1516 and the calcifying PLY M217 strains. Cells were grown at higher cell densities and were not in log phase but were transitioning from exponential to stationary phase growth during which time cell growth slows, nutrients become limiting, and calcification is enhanced (Bodt et al., 2008; Shiraiwa 2003; Paasche., 2001). Cell counts were determined using a hemocytomer and represent average values obtained from three experimental replicates. Error bars represent the standard deviations, some of which are too small to be seen. Although the doubling times of 42.3 and 44.9 hr, and the growth rates of 0.16 and 0.15, respectively for M217 (B, left panel) and CCMP1516 (B, right panel) are similar, the final cell density is higher for CCMP1516. Light micrographs in panel B were taken at 100X.
