# Supplementary figures and images for: Characterization of the Small RNA Transcriptome of the Marine Coccolithophorid, Emiliania huxleyi
Source: PLoS One. 2016 Apr 21;11(4):e0154279. doi: 10.1371/journal.pone.0154279 (PMC4839659; doi:10.1371/journal.pone.0154279)

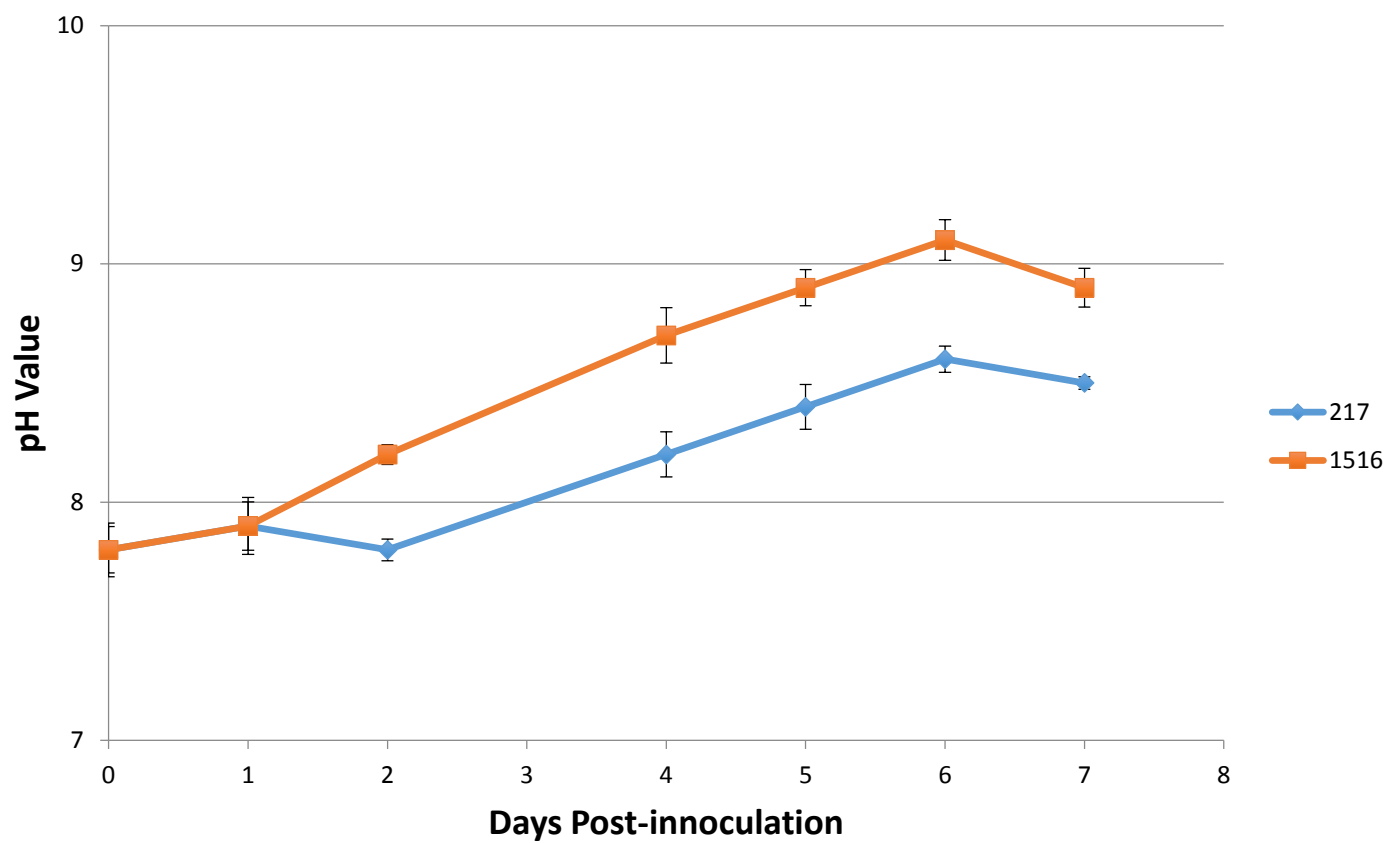

S2 Fig. pH values of the filtered seawater media for growing *E. huxleyi* strains.

Supplement: S2 Fig — (PDF) [file pone.0154279.s002.pdf]

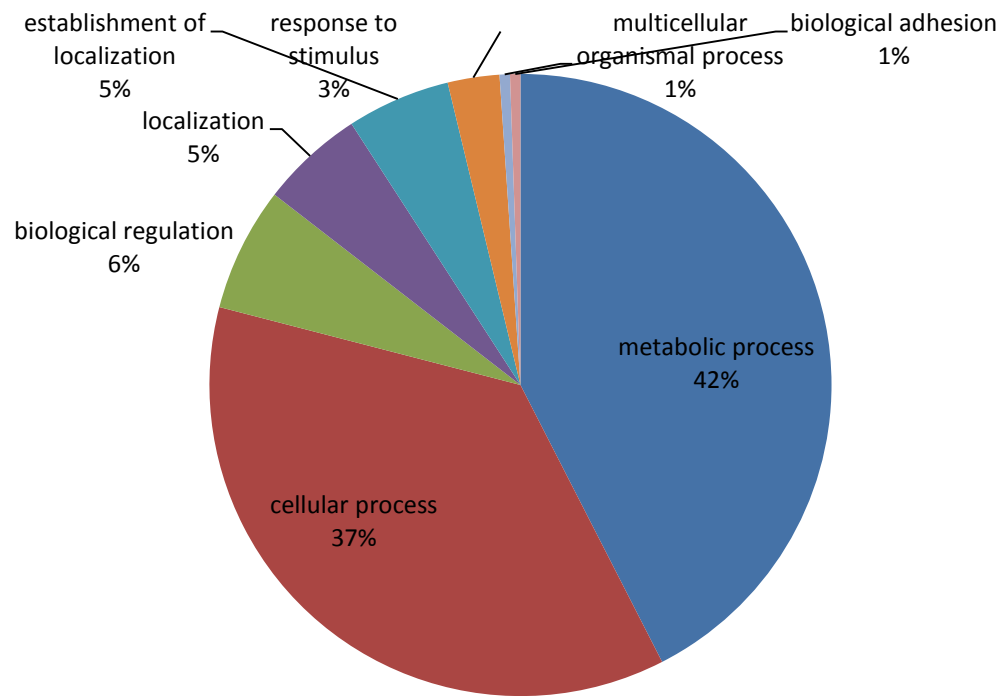

**S7 Fig. Distribution of 2nd level Gene Ontology terms in Biological Process for ta-siRNA targets.**

Supplement: S7 Fig — (PDF) [file pone.0154279.s007.pdf]
