# Supplementary material for: Characterization of the Small RNA Transcriptome of the Marine Coccolithophorid, Emiliania huxleyi
Source: PLoS One. 2016 Apr 21;11(4):e0154279. doi: 10.1371/journal.pone.0154279 (PMC4839659; doi:10.1371/journal.pone.0154279)
Supplement: S3 Fig — (PDF) [file pone.0154279.s003.pdf]

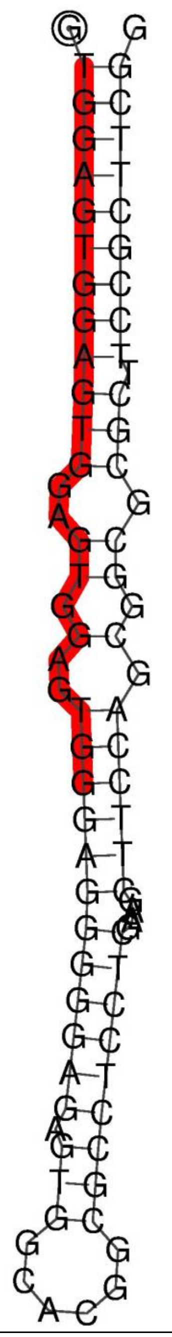

mir01 (len = 82)

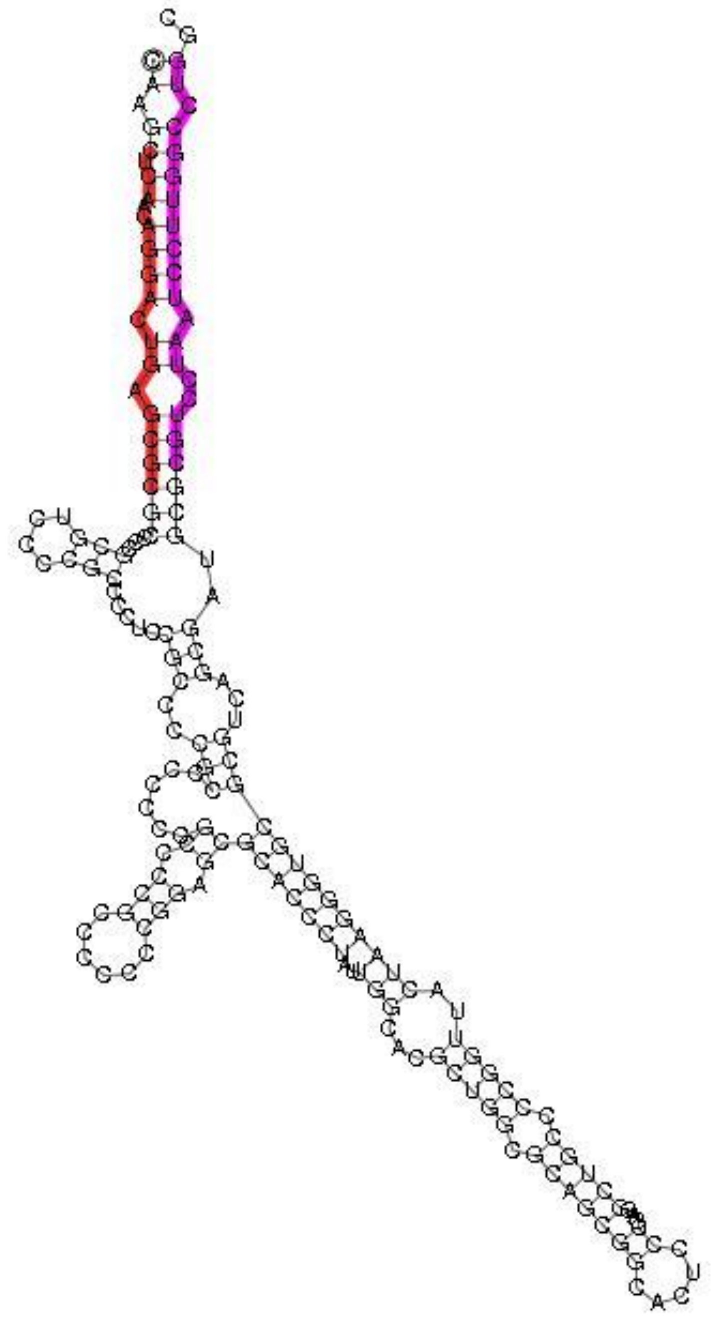

mir02 (len = 170)

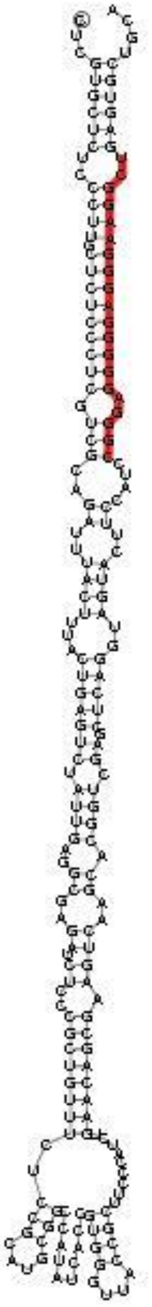

mir03 (len = 193)

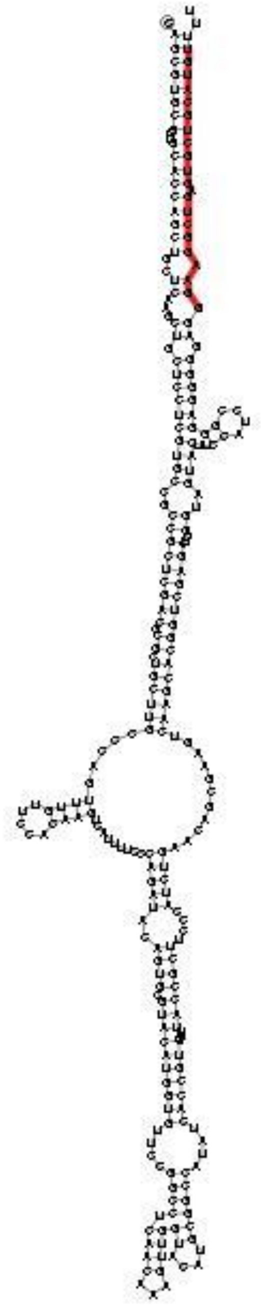

mir04 (len = 252)

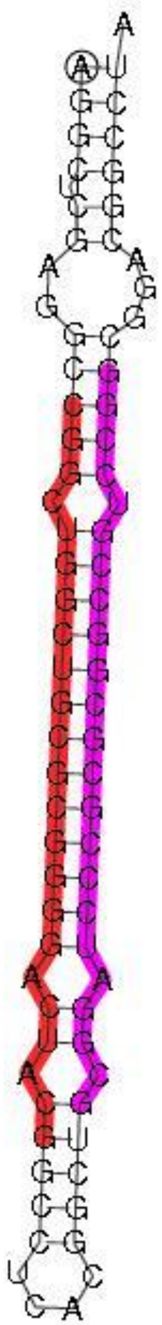

mir05 (len = 79)

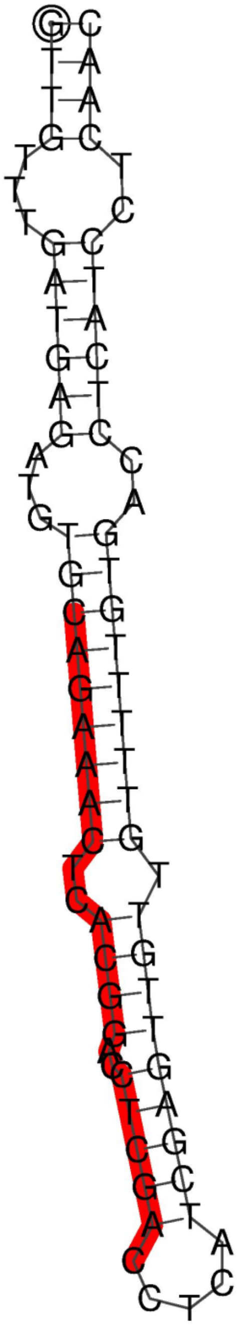

mir06 (len = 76)

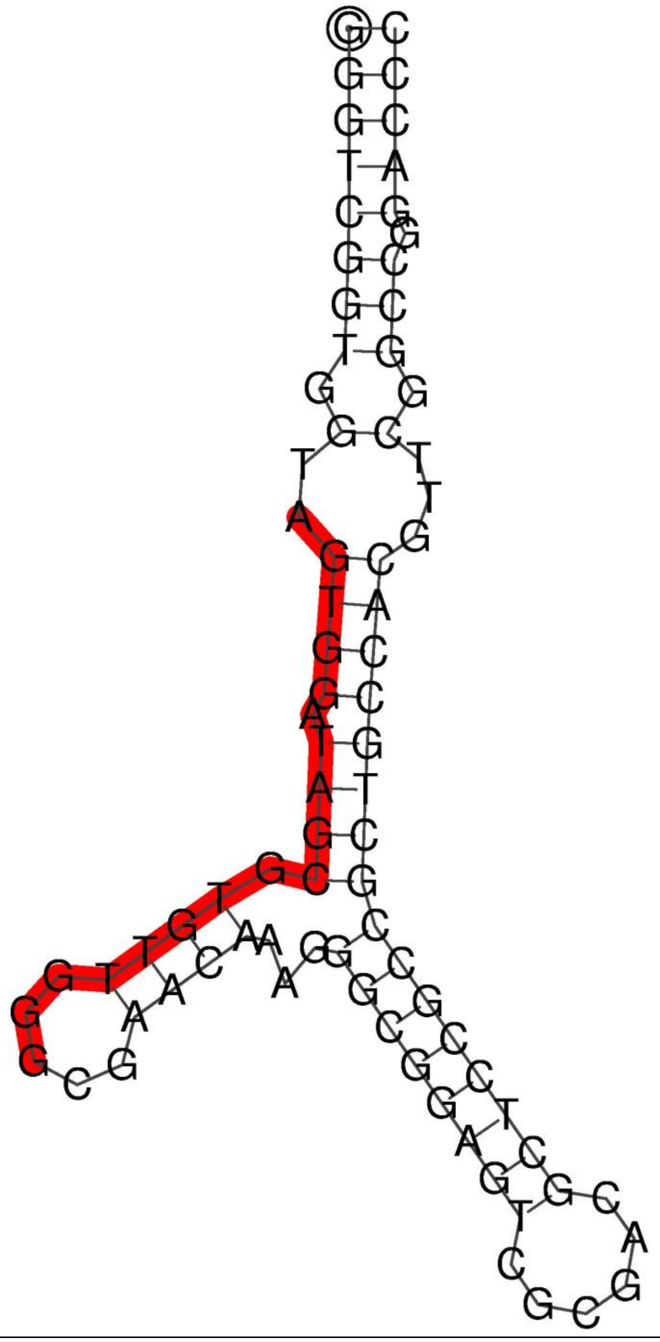

mir07 (len = 82)

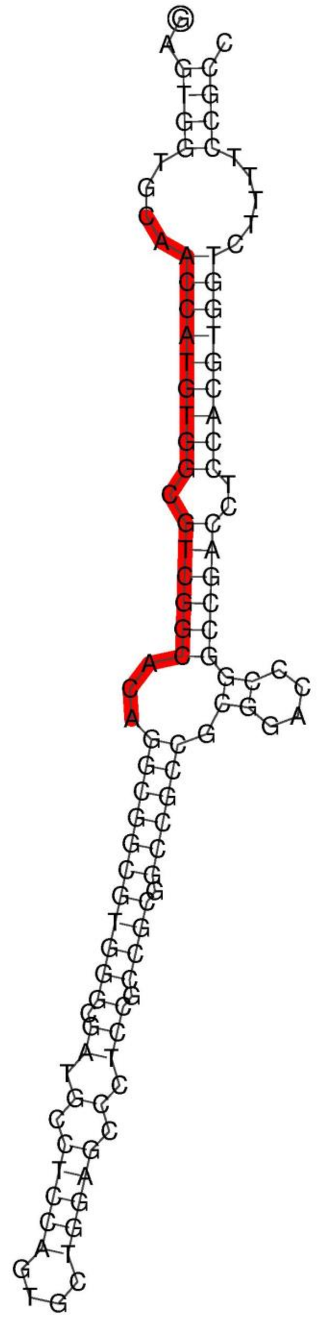

mir08 (len = 114)

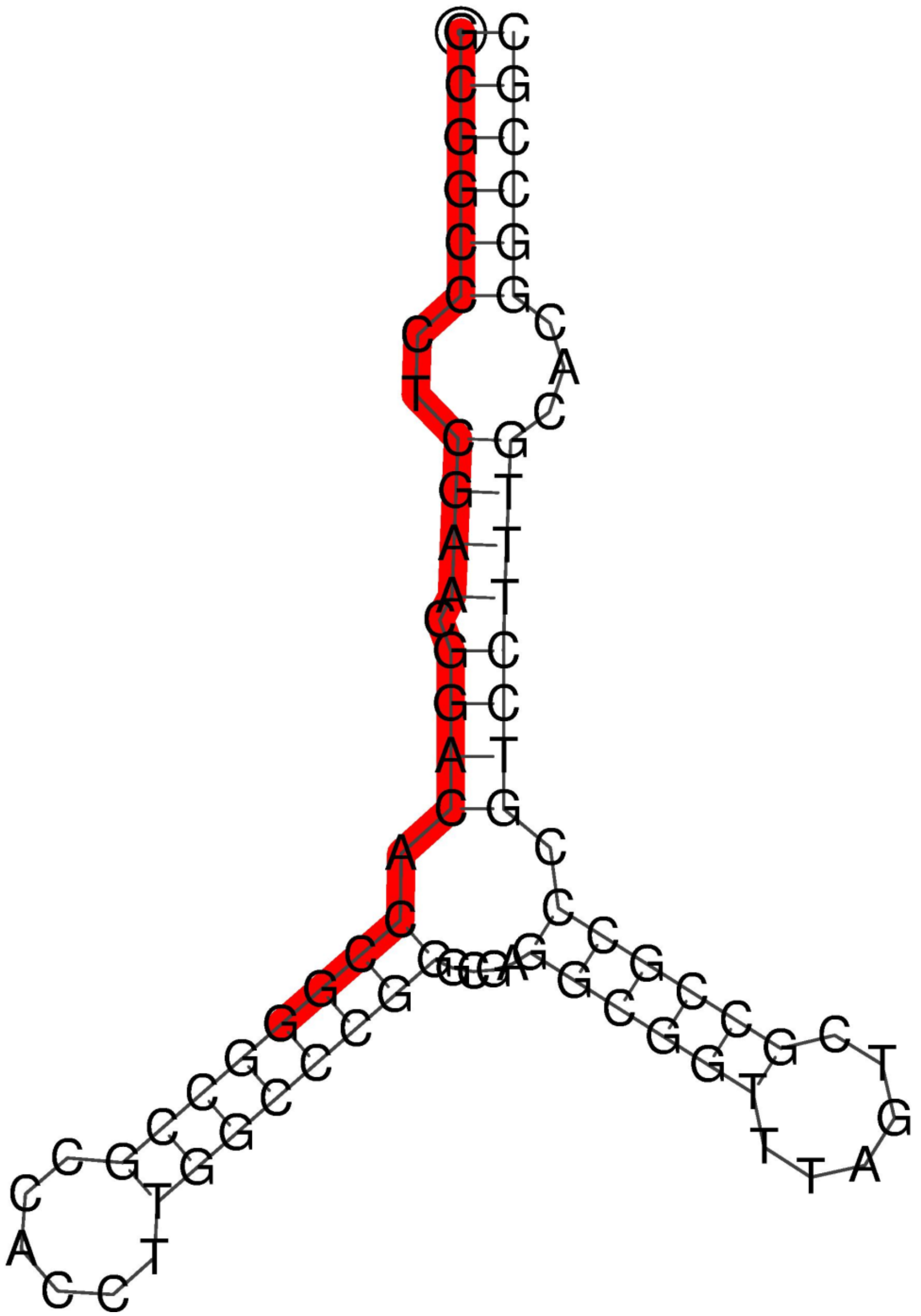

mir09 (len = 80)

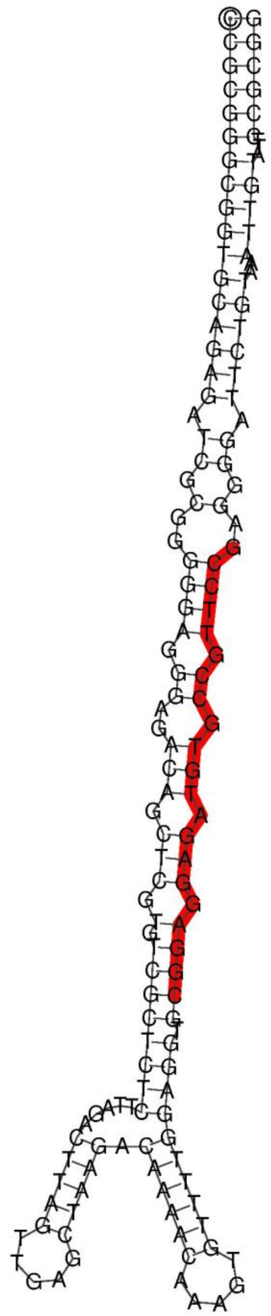

mir10 (len = 144)

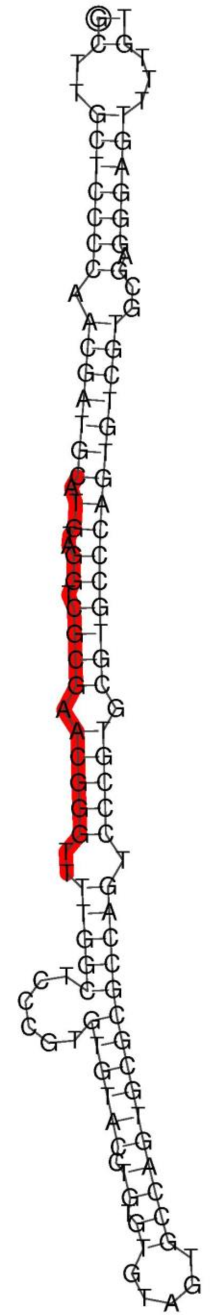

mir11 (len = 119)

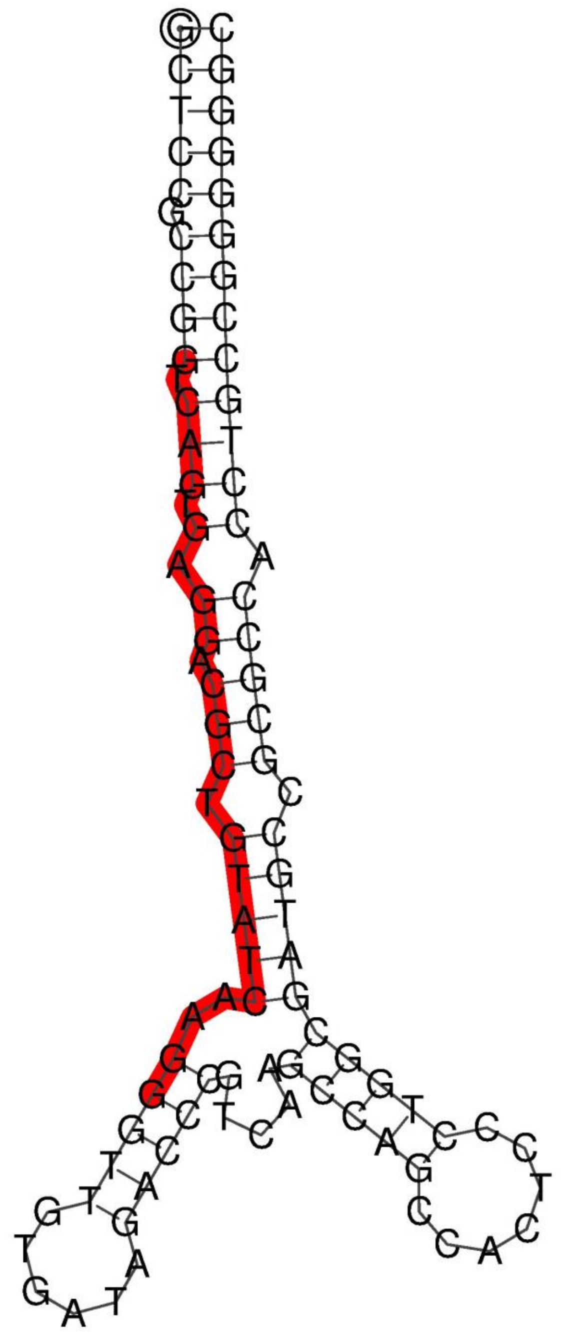

mir12 (len = 94)

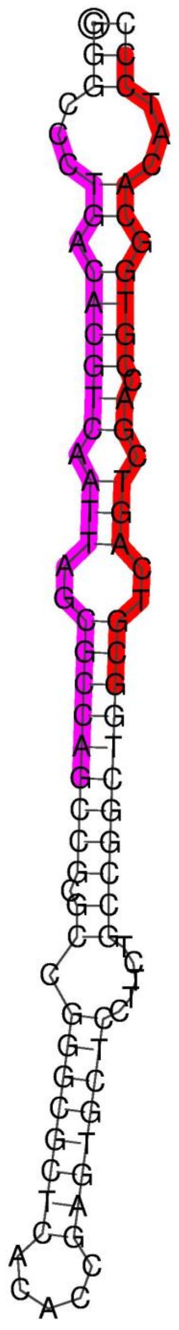

mir13 (len = 93)

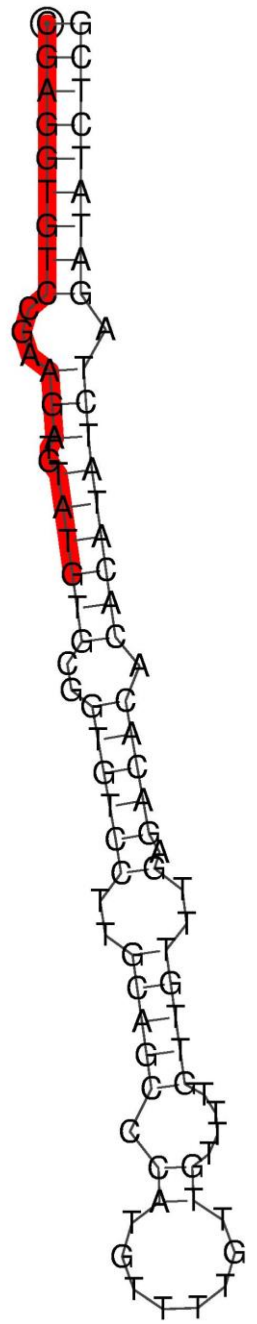

mir14 (len = 89)

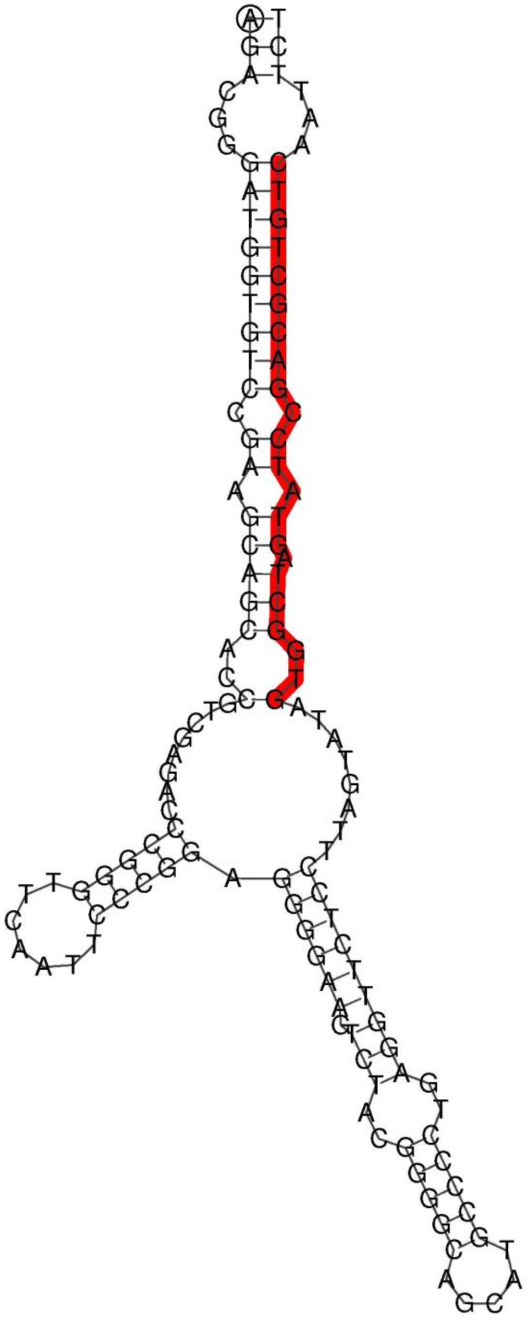

mir15 (len = 127)

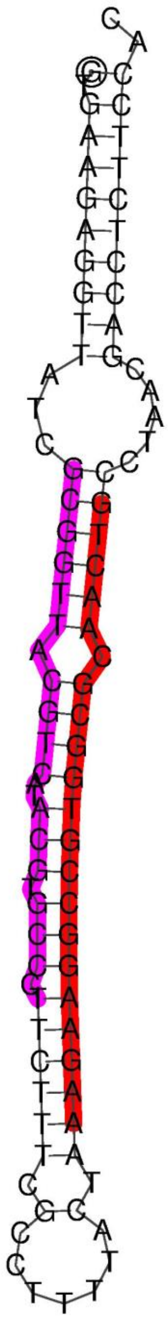

mir16 (len = 92)

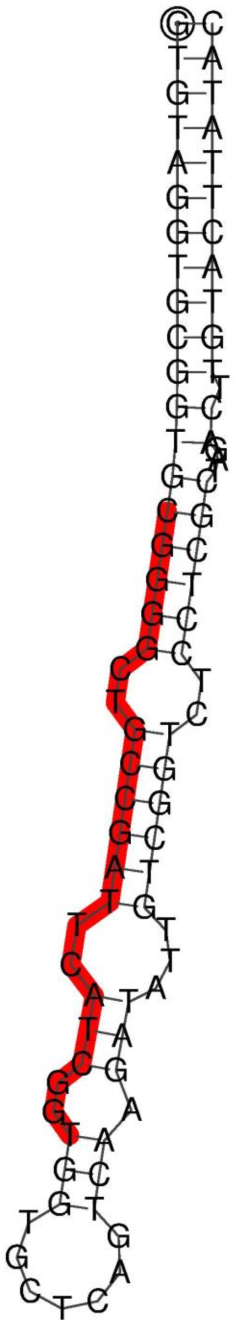

mir17 (len = 85)

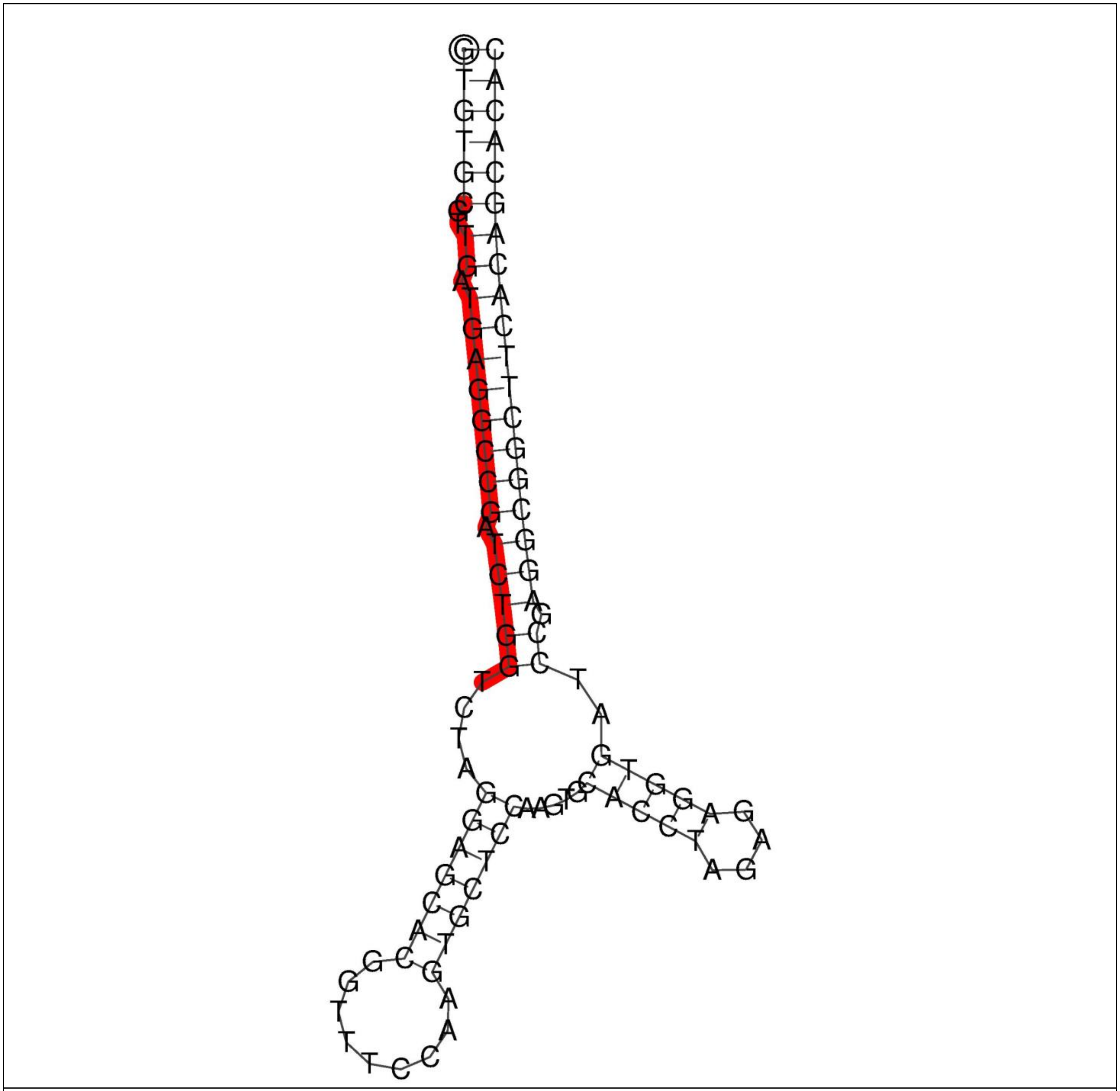

mir18 (len = 95)

S3 Fig. miRNA precursors predicted from small RNA reads, in which the predicted miRNAs are colored red and miRNA*'s are colored in purple.
