# Supplementary material for: Characterization of the Small RNA Transcriptome of the Marine Coccolithophorid, Emiliania huxleyi
Source: PLoS One. 2016 Apr 21;11(4):e0154279. doi: 10.1371/journal.pone.0154279 (PMC4839659; doi:10.1371/journal.pone.0154279)
Supplement: S4 Fig — (PDF) [file pone.0154279.s004.pdf]

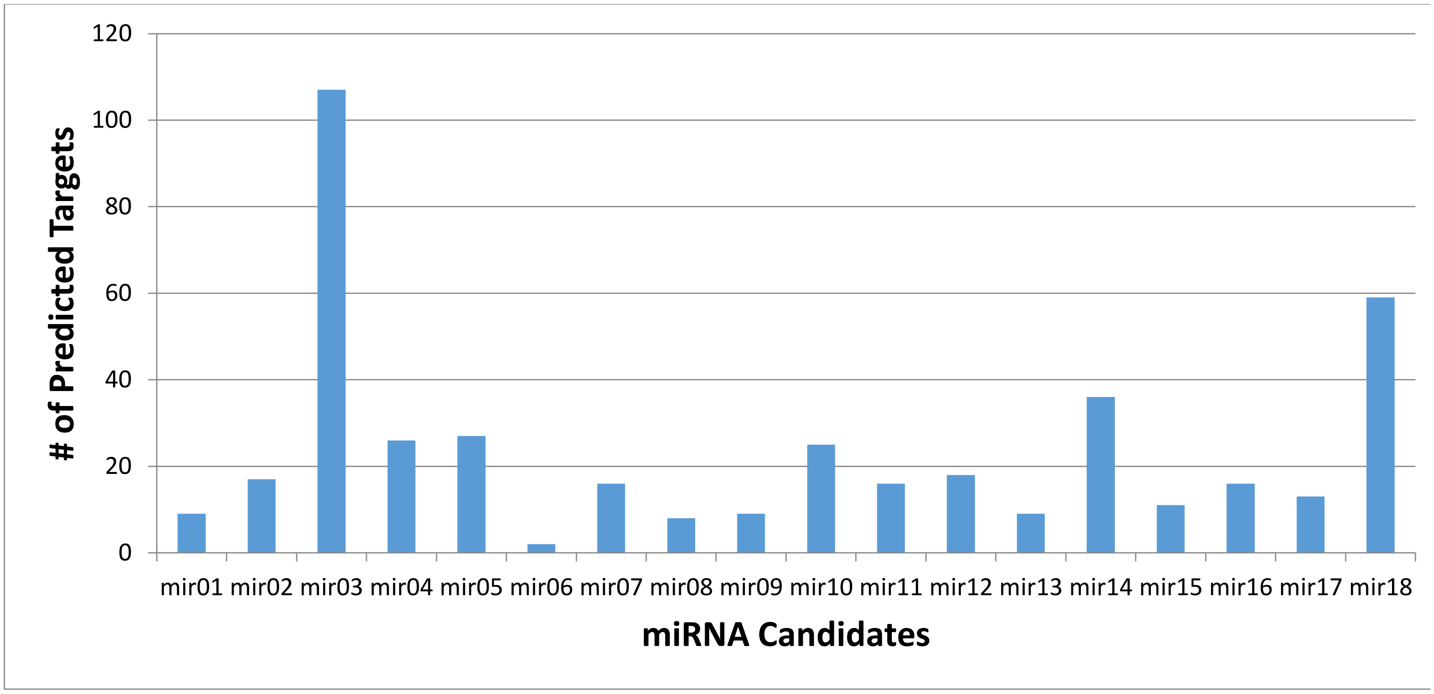

(a) plant-like binding characteristics

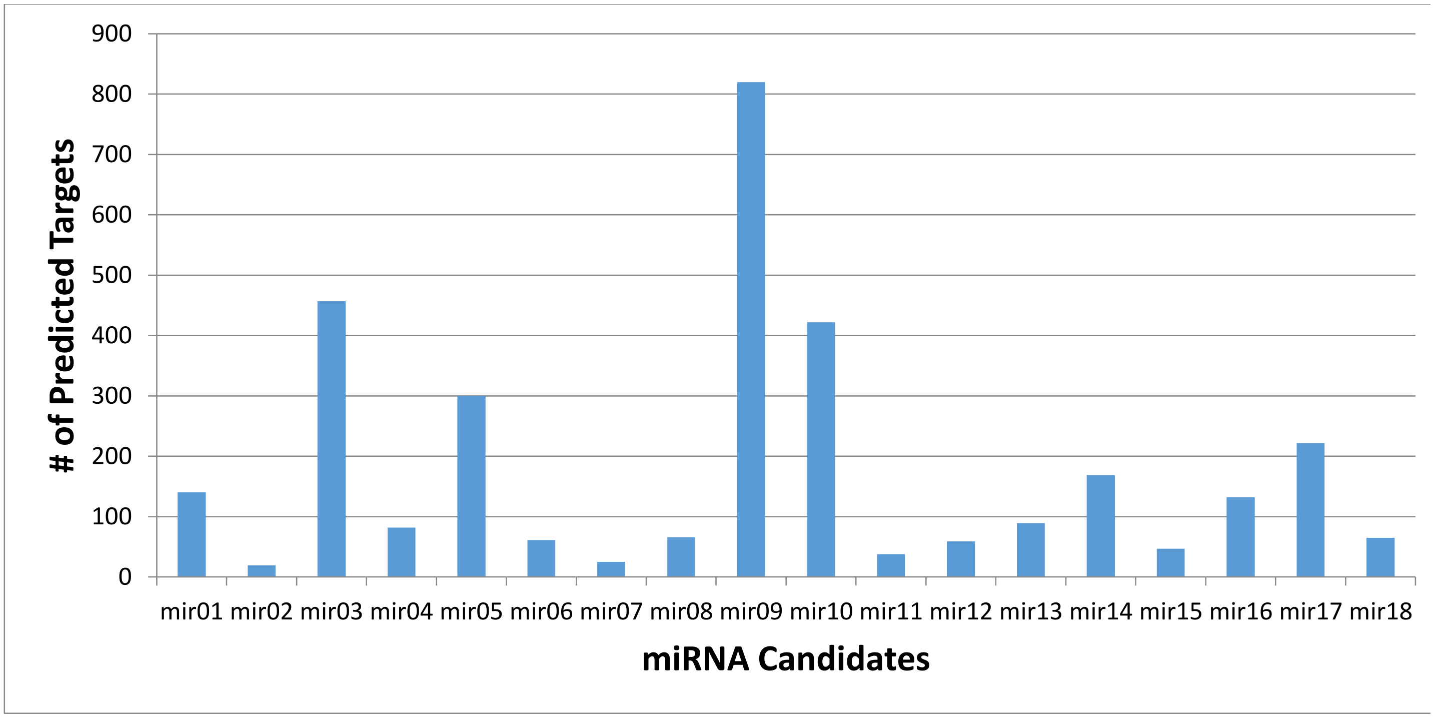

(b) animal-like binding characteristics

S4 Fig. Number of targets predicted using (a) plant-like binding characteristics and (b) animal-like binding characteristics.
