# Supplementary material for: Characterization of the Small RNA Transcriptome of the Marine Coccolithophorid, Emiliania huxleyi
Source: PLoS One. 2016 Apr 21;11(4):e0154279. doi: 10.1371/journal.pone.0154279 (PMC4839659; doi:10.1371/journal.pone.0154279)
Supplement: S5 Fig — (PDF) [file pone.0154279.s005.pdf]

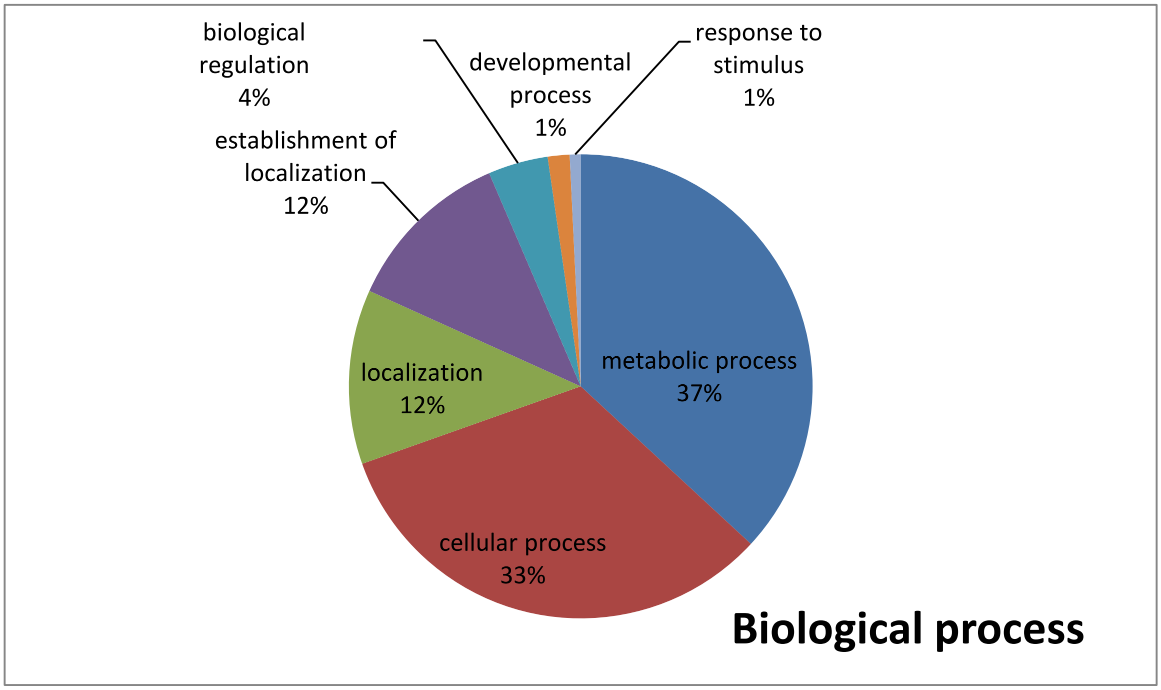


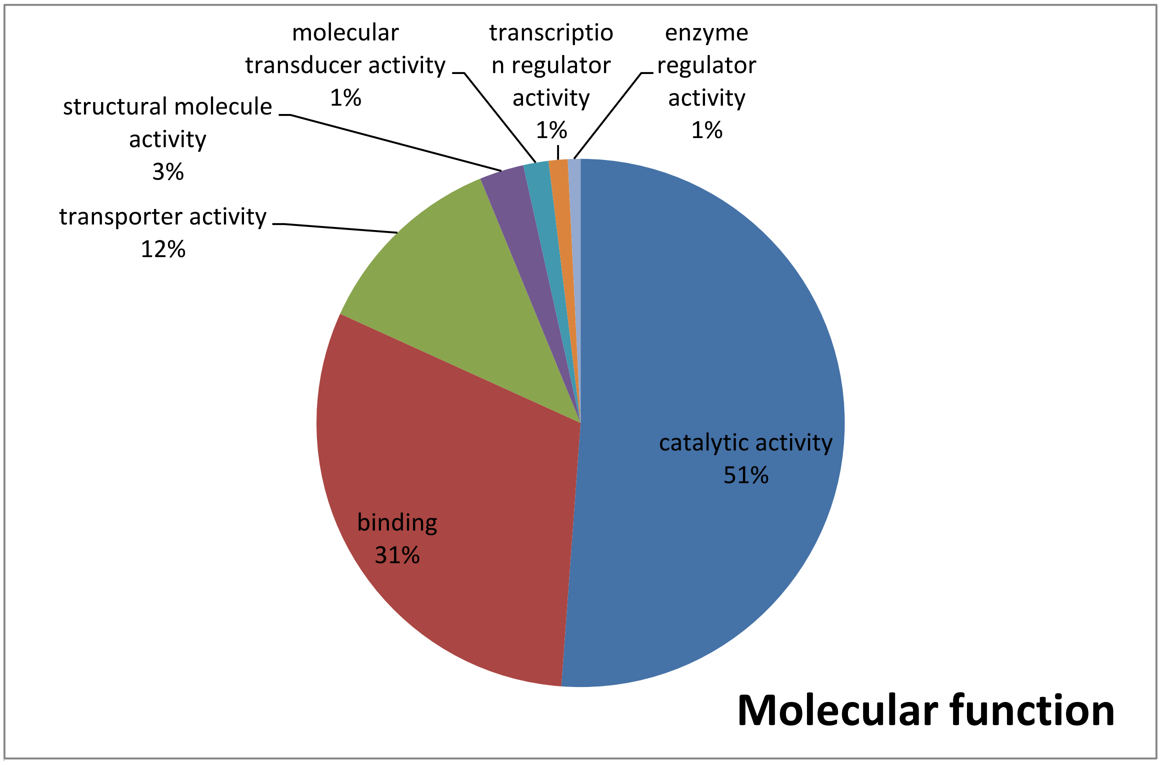


**S5 Fig. Distributions of level-2 Gene Ontology terms for predicted miRNA target genes with plant-like binding characteristics.**
