# Supplementary material for: Characterization of the Small RNA Transcriptome of the Marine Coccolithophorid, Emiliania huxleyi
Source: PLoS One. 2016 Apr 21;11(4):e0154279. doi: 10.1371/journal.pone.0154279 (PMC4839659; doi:10.1371/journal.pone.0154279)
Supplement: S6 Fig — (PDF) [file pone.0154279.s006.pdf]

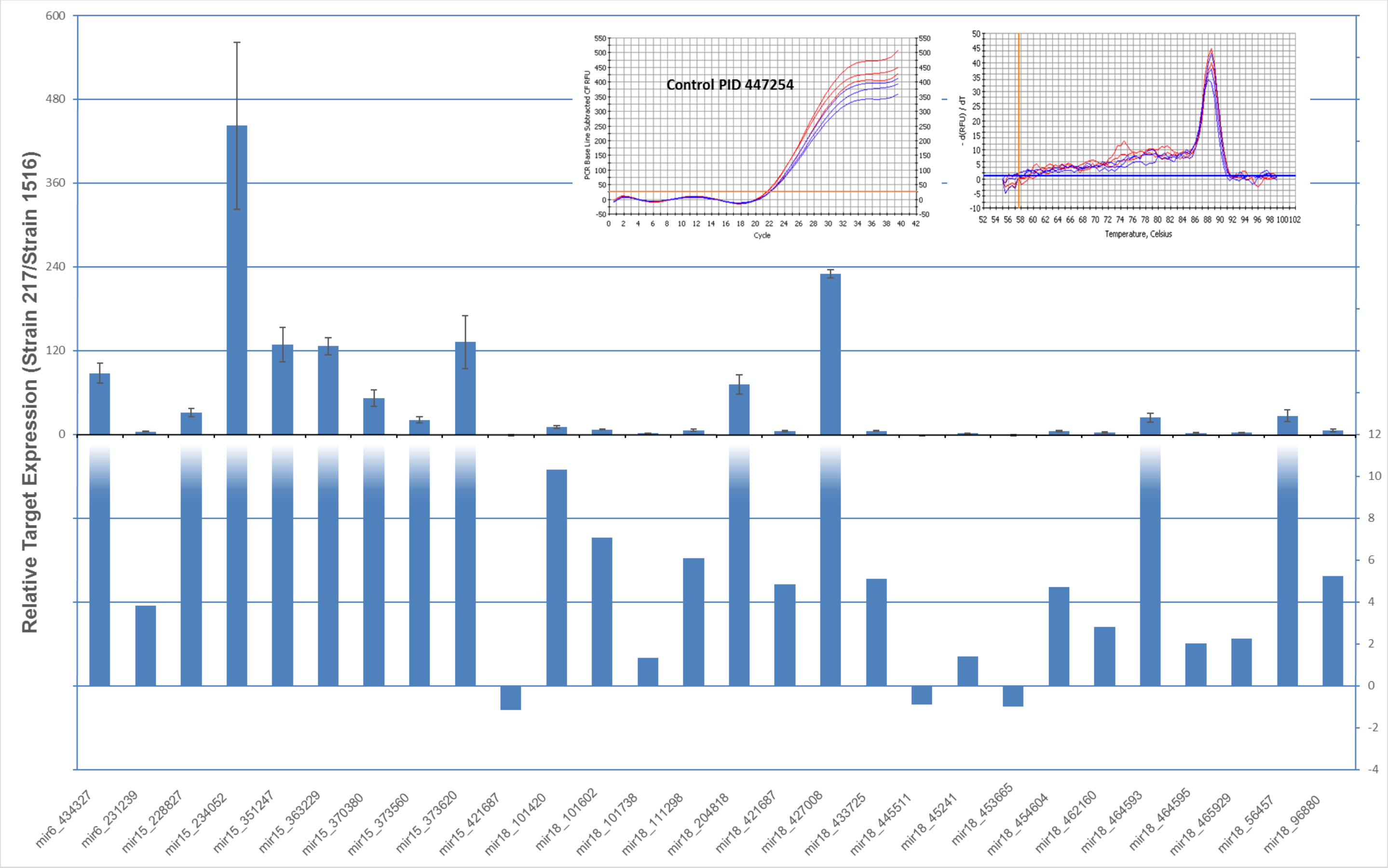

S6 Fig. Relative expression of a subset of miRNA target genes between calcifying strain 217 and non-calcifying strain 1516.
