# Supplementary material for: Characterization of the Small RNA Transcriptome of the Marine Coccolithophorid, Emiliania huxleyi
Source: PLoS One. 2016 Apr 21;11(4):e0154279. doi: 10.1371/journal.pone.0154279 (PMC4839659; doi:10.1371/journal.pone.0154279)
Supplement: S8 Fig — The RNAse III domain located in different regions in the two proteins is highlighted in blue. (PDF) [file pone.0154279.s008.pdf]

```
jgi|Emihu1|110711|fgeneshEH_pg      --------------------------------------------------
gi|158276338|gb|EDP02111.1|         MGTALPPLREYQAELLRMARRHASTVIYAETGTGKTRIAVERILEAHGEL 50


jgi|Emihu1|110711|fgeneshEH_pg      --------------------------------------------------
gi|158276338|gb|EDP02111.1|         VAAGRLAAFLAPTVPLVRQQAEVLVAAGLRVVVYTGEDNRDARNRAGWQR 100


jgi|Emihu1|110711|fgeneshEH_pg      --------------------------------------------------
gi|158276338|gb|EDP02111.1|         LHQEADVLCATPDALNRLIAHAFITIPQLGMIVFDECHHTNANHPYAVLL 150


jgi|Emihu1|110711|fgeneshEH_pg      ----------------------------MLLLAAHLLVTPCRHHATPISM 22
gi|158276338|gb|EDP02111.1|         RVFVRHLTPPDQRPVLLGLTASPHKADELMRLLGAQMVAASNTAQLQTFM 200
                                                                :: * .  :*:...       *

jgi|Emihu1|110711|fgeneshEH_pg      AEALEALPRRELQALAKQS------------------------------- 41
gi|158276338|gb|EDP02111.1|         AKPRVEVHRQEVEQPATVAPPQRRLASGTFSAAASILPPECAAAVASGDA 250
                                    *:.   : *:*::  *. :

jgi|Emihu1|110711|fgeneshEH_pg      --------GLRANQKSSVLIAQLSGRNG---------------------- 61
gi|158276338|gb|EDP02111.1|         ADMRKAGEALHAGGGSCIAAQPMAALDGAIELVSAAVEYSRCRDQTFRRC 300
                                            .*:*.  *.:    ::. :*

jgi|Emihu1|110711|fgeneshEH_pg      --------------------------------------------------
gi|158276338|gb|EDP02111.1|         CALGEPLPEMRAPRYTSPLLRGMETPQRWWEQDPQCGTKHLEHLRSQLAG 350


jgi|Emihu1|110711|fgeneshEH_pg      --------GVWPPAGVAGAPVDLPAAEDED----LRLLSRRE------LQ 93
gi|158276338|gb|EDP02111.1|         VKDALSSMGLWPAVAVAAADLFGPTAAPPQPATLLRLLDSEEGSAWPHLQ 400
                                            *:**...**.* :  *:*   :    ****. .*      **

jgi|Emihu1|110711|fgeneshEH_pg      EVAKRRGVRANAKSSEIISALESLRHADRAAPEPDPAPAPPEAAAPVEAA 143
gi|158276338|gb|EDP02111.1|         QRKRLRRGRDNEQRPVPPAAEDVDSSDSDCEDEGDNLPAEHSSSAAGEGE 450
                                    :  : *  * * : .   :* :     . .  * *  **  .::*. *.

jgi|Emihu1|110711|fgeneshEH_pg      DVG----------------------------------------------L 147
gi|158276338|gb|EDP02111.1|         DEGDEADEATLWGYQIGELLEEGGLGDGSEASSIADHRDRAALLVSGLAV 500
                                    * *                                              :

jgi|Emihu1|110711|fgeneshEH_pg      DLVERARPAVLTPAPCAGG---RWQPIRLRGP---SLELTLLVRDVG--- 188
gi|158276338|gb|EDP02111.1|         MLPPALREALHPLAARDRGDAQPWAELLLRAVPLNKLQQQVLGQDLPSRP 550
                                     *    * *: . *.   *    *  : **.    .*:  :* :*:

jgi|Emihu1|110711|fgeneshEH_pg      TPVGRLDVPPLSIVGERLGGAEVEAEVLPVLELGEE---------EVALA 229
gi|158276338|gb|EDP02111.1|         AAAATAAVPLAAVVGVPLVTPAVQWVVRSLLALSTQRHPADSGGDGVSVS 600
                                    :...   **  ::**  *  . *:  * .:* *. :          *:::

jgi|Emihu1|110711|fgeneshEH_pg      AGWASTLTGLLGIDEPGLFPAPAQMLHADG------VSSGSAGGAGEAAV 273
gi|158276338|gb|EDP02111.1|         SGWSAMVFCQRKVACVALHRLLTELPAARGGAIRPAVFMGNTGAGHSASS 650
                                    :**:: :     :   .*.   :::  * *      *  *.:*.. .*:

jgi|Emihu1|110711|fgeneshEH_pg      LFW----------------------------------------------- 276
gi|158276338|gb|EDP02111.1|         LAMDGRKQERVRRAFSSGALNVLISTSVGAEGLDFRTCNAVIMVDPPDHV 700
                                    *

jgi|Emihu1|110711|fgeneshEH_pg      -PQTEVGGGAGAAGERRALDALRDP-----AQWERLTADPGGAEPRATEL 320
gi|158276338|gb|EDP02111.1|         TPFVQCAGRARAPGSCYLLFARDDRQQQAIAKRSREEADMVAKALKLSTA 750
                                     * .: .* * *.*.   * *  *      *: .*  **  .   : :

jgi|Emihu1|110711|fgeneshEH_pg      GTAELGTAEPGDAAAWPEDGEGEPVVVLEHRRPEWVALTPTLR------- 363
gi|158276338|gb|EDP02111.1|         AAREAQQRQQTDPPVNPFFSQDVDVVTEELQQNLFRNLLESYCRTLPGNS 800
                                    .: *    :  *... *  .:.  **. * ::  :  *  :

jgi|Emihu1|110711|fgeneshEH_pg      -----------------LSRGVAGLARVNPDRNEQSGVGEKTHRNPKN-- 394
gi|158276338|gb|EDP02111.1|         GDVLLQPYYSYQEYESPLTRRKAFVATVHMPANSPLGVVEGPMQSNKGDA 850
                                                     *:*  * :* *:   *.  ** * . :. *.
```

```
jgi|Emihu1|110711|fgeneshEH_pg      ---------------------------------ALQEAAYRRGLPPPSYA 411
gi|158276338|gb|EDP02111.1|         RQHACMATVRRLYELGALDEHLLPRFTRRGRMREAQQQDLRGGGAATAAA 900
                                                                       *:   * * ...: *

jgi|Emihu1|110711|fgeneshEH_pg      SEQLSEQVWAATLS--------------------LEGYGHFRGEPAPRKR 441
gi|158276338|gb|EDP02111.1|         ATGRTRHRFGVVLHRRLPCDLPPFTAYLPPEDDSEAADGPQGQQPAPVAV 950
                                    :   :.: :...*                       . *    :***

jgi|Emihu1|110711|fgeneshEH_pg      ----LAEMAAARLGLRAVEEG-----------------------AEPPAA 464
gi|158276338|gb|EDP02111.1|         RLEYLGPLRLSAAQLAALETAGGVMESLLITRGAQPVATSSSATADKPVA 1000
                                        *. :  :   * *:* .                       *: *.*

jgi|Emihu1|110711|fgeneshEH_pg      AEPPAAAAEEP--------------------AVLPPGGVGSPKARLGRLA 494
gi|158276338|gb|EDP02111.1|         APGPASAGPVPGSPALAAWSERLGPGLAALRRALAAAGIKTSAAYVHEAV 1050
                                    *  **:*.  *                     .*...*: :. * : . .

jgi|Emihu1|110711|fgeneshEH_pg      T-------------------RAGGYVAYSVEHLGHQLFRATVRLAGIEAA 525
gi|158276338|gb|EDP02111.1|         NNVPDRMNSTAGEPATAAQLPASGHWWWLVAPLSEQTQAASSQTAGTEAA 1100
                                    .                    *.*:  : *  *..*   *: : ** ***

jgi|Emihu1|110711|fgeneshEH_pg      SP-------------------------AAWPPAAGLDAAGEPARSKKLAA 550
gi|158276338|gb|EDP02111.1|         AAGAVDWGFLARLAAGPVPLQALCPQLPALRPLAQQPAQGHQQQRQQLLP 1150
                                    :.                         .*  * *   * *.  : ::* .

jgi|Emihu1|110711|fgeneshEH_pg      QSAATVALGEWG-------------------------------------- 562
gi|158276338|gb|EDP02111.1|         AAATGADVGSVGLHGHRQAAAPGGQHVELLTAAVREALRGSGGLLITQTG 1200
                                     :*: . :*. *

jgi|Emihu1|110711|fgeneshEH_pg      ---------------------------------------------DQLAL 567
gi|158276338|gb|EDP02111.1|         ATLHALRGLTAVAEHDEAGTGSRKAAQDAARLVERWGVDPSCLLPDQPQV 1250
                                                                                 **  :

jgi|Emihu1|110711|fgeneshEH_pg      LCEPG---------------------ADAAGEEGEGGGAVLR-------L 589
gi|158276338|gb|EDP02111.1|         LVDAGTATRTASNVLLAPPQPEHHPHSHHGAEDGSREAAAVISMLPQALA 1300
                                    * :.*                     :. ..*:*.  .*.:

jgi|Emihu1|110711|fgeneshEH_pg      ALPLRASQWRGAACAVVMAR-----VLAHAAEVRLLRAD----LLTLLPA 630
gi|158276338|gb|EDP02111.1|         VAPLSVRCWRALHRTVSLVHRLEGLLVAAEAEEELLRPAGFLRPAATAAA 1350
                                    . ** .  **.   :* :.:     ::*  ** .***.       :  .*

jgi|Emihu1|110711|fgeneshEH_pg      ASVPSYATLCGATTPRSIGGGGEGSNEMSAWVGNAVLGLCCKVAAVLALG 680
gi|158276338|gb|EDP02111.1|         SGNPVLAIPVPTTAPSTSTATTAAATPQQAAVLLALTALTARAAADPAFD 1400
                                    :. *  *    :*:* :  .   .:.  .* *  *: .* .:.**  *:.

jgi|Emihu1|110711|fgeneshEH_pg      AP-------------------------PRRAQQVFSPHYYRWLGTSWLAG 705
gi|158276338|gb|EDP02111.1|         CERLETLGDAVLKYLATLYVYGTERDVPVSHEGVMSYKRDQLVANEALYG 1450
                                    .                          *   : *:* :  : :... * *

jgi|Emihu1|110711|fgeneshEH_pg      RVRSRGWEKALFLQPWAGESGITR-----GGASPETQKEVG-------EA 743
gi|158276338|gb|EDP02111.1|         RALEAGLQHHMRALPYDMERVLRGRHWNTGEEAARAQEVRGKRLADCVEA 1500
                                    *. . * :: :   *:  *  :       *  :..:*:  *       **

jgi|Emihu1|110711|fgeneshEH_pg      VIGAVFLHSLAAHG------------------------------------ 757
gi|158276338|gb|EDP02111.1|         LVGCHLAPGVPTAGSSSNSNDAQATSPGSASAPVLVPVSATNPVPGAPCQ 1550
                                    ::*. :  .:.: *

jgi|Emihu1|110711|fgeneshEH_pg      ----------------------------------VDRAMEDAWRLCS--- 770
gi|158276338|gb|EDP02111.1|         DGSALAALVAALRQTCAPASGHTGVTDPSSNSGDVHARLDAALRFCCGLG 1600
                                                                      *.  :: * *:*.

jgi|Emihu1|110711|fgeneshEH_pg      -----------RVVLRGEGSVAEG----------------WGEALAVLRP 793
gi|158276338|gb|EDP02111.1|         VLPGAAPAVLQRLHAEGQGGAGEGAVQGVPDVGGEAGRAAVGALAAATGY 1650
                                               *:  .*:*...**                 *   *.

jgi|Emihu1|110711|fgeneshEH_pg      RLVARRPGPVDLLSLPWPSEP------GLFASAASREWEG---QSLEFVG 834
gi|158276338|gb|EDP02111.1|         QFHDPALALCALTHVSWPVPPVSSSGKGLDAGAGSGGGSGGHYQLLEFLG 1700
```

```
                                    ::     .   *  :.**  *      ** *.*.*   .*   * ***:*

jgi|Emihu1|110711|fgeneshEH_pg      DGVLRVLHSLHLLESLPGSTERGVRAAARIAMERNEFLARRISRVVGTDG 884
gi|158276338|gb|EDP02111.1|         DAVVGLLASLWAYS--LGGSPRDMSMTREMLVRNDTLAACCLGSRLSTAL 1748
                                    *.*: :* **   .   *.: *.:  : .: :..: : *  :.  :.*

jgi|Emihu1|110711|fgeneshEH_pg      SWLTSKLRLARDEVQRSMAEQDQLQLG--------------DEEAFRLDL 920
gi|158276338|gb|EDP02111.1|         RVRHRQLQLAIEEYGVALLMSYDTTSGRADAAATGGGGPLIRAEMWKEAM 1798
                                         :*:** :*   ::  . :   *                * ::  :

jgi|Emihu1|110711|fgeneshEH_pg      AASLPDESTKVLADVLEALVG----------AVAVQDGLERAG------- 953
gi|158276338|gb|EDP02111.1|         AARDLRVATLLQSAWLEVEGGGTGEGVQAATDAAPGGGLHRGRRAYGLSG 1848
                                    **     :* : :  **.  *           .*  .**.*.

jgi|Emihu1|110711|fgeneshEH_pg      --------------AAFAQVVLPPQCVVDELADGRVAGDVTQLPAGSGGA 989
gi|158276338|gb|EDP02111.1|         GDCDYRCTPMAAPPQLWRHGSIPHHTAQLSHVARHVRPGSCMLAAGGSGH 1898
                                                    : :  :* : .  . .  :*  .   *.**..*

jgi|Emihu1|110711|fgeneshEH_pg      SRWSASA---------------------- 996
gi|158276338|gb|EDP02111.1|         GGNGAAVRGSWLRVRSANVPVARVARVLA 1927
                                    .  .*:.
```

S8 Fig. Multiple sequence alignment of the DICER or DICER-like protein from *E. huxleyi* (PID: 110711) with the DICER-like protein from *Chlamydomonas reinhardtii* (EDP02111.1) where the sequence homology is 50% and sequence identity is 34%. The RNAse III domain located in different regions in the two proteins is highlighted in blue.
