# Supplementary material for: Characterization of the Small RNA Transcriptome of the Marine Coccolithophorid, Emiliania huxleyi
Source: PLoS One. 2016 Apr 21;11(4):e0154279. doi: 10.1371/journal.pone.0154279 (PMC4839659; doi:10.1371/journal.pone.0154279)
Supplement: S9 Fig — Two of the four catalytic residues of the RNase III domain identified by [75] are conserved in E. huxleyi and C. reinhardii and are highlighted in red. (PDF) [file pone.0154279.s009.pdf]

```
Chlamy          ERLETLGDAVLKYLATLYVYG---TERDVPVSHEGVMSYKRDQLVANEALYGRALEAG-L 56
Ehux            QSLEFVGDGVLRVLHSLHLLESLPGSTERGVRAAARIAMERNEFLARRISRVVGTDGSWL 60
                : ** :**.**: * :*::      . :  *   . :: :*::::*..     . :.. *

Chlamy          QHHMRALPYDMERVLRGR-HWNTGEEAARAQEVRG-------KRLADCVEALVGCHL--- 105
Ehux            TSKLRLARDEVQRSMAEQDQLQLGDEEAFRLDLAASLPDESTKVLADVLEALVGAVAVQD 120
                  ::*    :::* :  : : : *:* *   :: .       * *** :*****.

Chlamy          -
Ehux            G 121
```

S9 Fig. Alignment of the RNAse III domains of the DICER-like proteins from *E. huxleyi* and *C. reinhardtii* share 29% amino acid identity and 45% homology. Two of the four catalytic residues of the RNase III domain identified by [75] are conserved in *E. huxleyi* and *C. reinhardii* and are highlighted in red.
