# Supplementary material for: Characterization of the Small RNA Transcriptome of the Marine Coccolithophorid, Emiliania huxleyi
Source: PLoS One. 2016 Apr 21;11(4):e0154279. doi: 10.1371/journal.pone.0154279 (PMC4839659; doi:10.1371/journal.pone.0154279)
Supplement: S10 Fig — Highlighted residues, several of which are invariant in the E. huxleyi argonaute (226029), are thought to stabilize the dsRNA-binding region. These include: 1) a subdomain of aromatic residues, 2) a cysteine residue preceded by a proline and a glutamine, and 3) other conserved residues that form a hydrophobic subdomain that interacts with RNA. (PDF) [file pone.0154279.s010.pdf]

```
Emiliania|414846|      -----------------------------------------LQQRHSPGVCKRLHGIDEV
Emiliania|226029|      -------------------------------------------------------DVSDK
Chlamy AGO             --------------------NSKNARRLEEALVGLEVQPS------YTANRYKLSGKLGK
Arabidopsis            PVIQFVCDLL-NRDISSRPLSDADRVKIKKALRGVKVEVTHR---GNMRRKYRISGLTAV
Homo sapiens           ---CEVLDIR-NIDEQPKPLTDSQRVRFTKEIKGLKVEVTHC---GQMKRKYRVCNVTRR
Drosophila AGO-1       ---CEVLDIR-DINEQRKPLTDSQRVKFTKEIKGLKIEITHC---GQMRRKYRVCNVTRR
Drosophila AGO-2       PMIEYLERFSLKAKINNTTNLDYSRRFLEPFLRGINVVYTPPQSFQSAPRVYRVNGLSRA
Chlamy AGO-like        -------------------------------------------------------GLSEQ
Nannochloropsis        --------------------DERQARAVSKAVKGLKIE----VTHRSSGRTYTVMGLSPQ


Emiliania|414846|      ALKDRTFTQD----DGN---ETNVYQYFVDNYPQFKREIDPNKPGI-----KLATEKTAE
Emiliania|226029|      LPREHFFETPQELRQQHPAAEISVQDFFRLRYGIELKH--PELVLVGFGKGG-RNLVPME
Chlamy AGO             SARSLAF--EF---EGR---------------KISVQH--PDLPCVL---DKKGGALPIE
Arabidopsis            ATRELTFPVDE---RNT---QKSVVEYFHETYGFRIQH--TQLPCLQVGNSNRPNYLPME
Homo sapiens           PASHQTFPLQL---ESGQTVECTVAQYFKQKYNLQLKY--PHLPCLQVGQEQKHTYLPLE
Drosophila AGO-1       PAQMQSFPLQL---ENGQTVECTVAKYFLDKYRMKLRY--PHLPCLQVGQEHKHTYLPLE
Drosophila AGO-2       PASSETF--EH---DGK---KVTIASYFHSR-NYPLKF--PQLHCLNVGSSIKSILLPIE
Chlamy AGO-like        GADRTMFMNEK---EGR---EMSVAEYFRST-GRPLRH--PGLPCANVGDRRRAVFIPVE
Nannochloropsis        PADQIRFMNEE---VGA---EQTIAAYFQSKYKKQLRY--PSLHCVRMGSKRKPTFFPIE
                             *                             :

Emiliania|414846|      VSSEMIKHMAEEPGFRFPAIEDIVRAV-----------
Emiliania|226029|      LCDFVGGEAARLATAD-ERSKLVK--------------
Chlamy AGO             LLTVVKYQKRMSRLDARQKADYIRTAALKPADRMRAMR
Arabidopsis            VCKIVEGQRYSKRLNERQITALLKVTC-----------
Homo sapiens           VCNIVAGQRCIKKLTDNQTSTMIKATAR----------
Drosophila AGO-1       VCNIVAGQRCIKKLTDMQTSTMIKATAR----------
Drosophila AGO-2       LCSIEEGQALNRKDGATQVANMIKYAA-----------
Chlamy AGO-like        LCTVVAGQRRMKLDATQ-SAGMI---------------
Nannochloropsis        VCKVVAGQRIPE--------------------------
```

S10 Fig. A comparison of the Paz domain of argonaute from *E. huxleyi*, *E. huxleyi* argonaute paralogs (226029; 46005; 414846), together with argonaute and argonaute-like homologs from *Homo sapiens* (NP_036331.1)*, Drosophila melanogaster* (NP_730054.1; NP_725341.1)*, Arabidopsis thaliana* (*NP*_849784.1)*, Nannochloropsis gaditana* (EWM22342.1) and *Chlamydomonas* (EDO99188.1; EDP01993.1). Highlighted residues, several of which are invariant in the *E. huxleyi* argonaute (226029), are thought to stabilize the dsRNA-binding region. These include: 1) a subdomain of aromatic residues, 2) a cysteine residue preceded by a proline and a glutamine, and 3) other conserved residues that form a hydrophobic subdomain that interacts with RNA.
