# Supplementary material for: Characterization of the Small RNA Transcriptome of the Marine Coccolithophorid, Emiliania huxleyi
Source: PLoS One. 2016 Apr 21;11(4):e0154279. doi: 10.1371/journal.pone.0154279 (PMC4839659; doi:10.1371/journal.pone.0154279)
Supplement: S11 Fig — Invariant residues are indicated by asterisks; highly residues by semicolon; and homologous residues by a period. Catalytic residues of the PIWI domain are highlighted in red. (PDF) [file pone.0154279.s011.pdf]

```
Chlamy AGO           MVMVVLPDRGGVGLYDAIKAAGDGKLGVATQCVNPAKAGLVGPKASSGPSFSYAAQLALS
Drosophila AGO-2     ---IVIIP-QFRISYDTIKQKAELQHGILTQCIKQFTV-------ERKCNNQTIGNILLK
Chlamy AGO-like      -VLVVLPE-KTADEYREVKRVSDIELGIPSQVVVASKA-RVGYRAHKGGGPQYCANVAMK
Nannochloropsis      -LVVCLIE-KEVPIYTEVKRAAETVHGVMTQCVTRDNV--------RKCNFMTNVNISLK
Arabidopsis          -LIVILPD-NNGSLYGDLKRICETELGIVSQCCLTKHV--------FKMSKQYMANVALK
Homo sapiens         -LIIVILP-GKTPVYAEVKRVGDTLLGMATQCVQVKNV--------VKTSPQTLSNLCLK
Drosophila AGO-1     -----------------------TVLGMATQCVQAKNV--------NKTSPQTLSNLCLK
Emiliania|414846|    -----------------------------------------------------------K
Emiliania|226029|    -------------------------------------------------CKGYWSNLLDK
Emiliania|46005|     ------------------------------------------------------------

Chlamy AGO           VNAKLGGATTRPA-----GRPNDWLPLLGGR---RLMVGGRGPGRLEARLGLSGALLSRG
Drosophila AGO-2     INSKLNGINHKIK-----DDPR--LPM-----MKNTMYIGADV--------------THP
Chlamy AGO-like      INNKLGGVNVQLS-----GGLR-NMPVLGGAGAVPFMVLGADV--------------THP
Nannochloropsis      VNEKLGGINSIVA-----KEH---MSY---IQKNRVMILGADV--------------NHA
Arabidopsis          INVKVGGRNTVLV-----DALSRRIPL---VSDRPTIIFGADV--------------THP
Homo sapiens         INVKLGGINNILV-----PHQ---RSA---VFQQPVIFLGADV--------------THP
Drosophilia AGO-1    INVKLGGINSILV-----PSI---RPK---VFNEPVIFLGADV--------------THP
Emiliania|414846|    VNLKLGGANGV------SKAMNQSKSV---VSAKPTMVVGLDV--------------NHA
Emiliania|226029|    LNLKALGTNYYPLCEVPGGGLQPGLQL---LRECPTLLLGCDV--------------HHA
Emiliania|46005|     ------------------------------------------------------------

Chlamy AGO           YRGAAQ-VSAGVEYAAVVGSADSH-AVDYRVQLSAQVAGN--------------------
Drosophila AGO-2     SPD----QREIPSVVGVAASHDPY-GASYNMQYRLQRGALEEIEDMF-------------
Chlamy AGO-like      TGAAARADSRDPSVAAVVASLDAS-LGRWASRVLLQAGRQEVITGMC-------------
Nannochloropsis      RVG----S-MRPSFAAVTGSLDAD-ARRHTAVLQAQSPRLEIIQDLS
Arabidopsis          HPG----EDSSPSIAAVVASQDWPEITKYAGLVCAQAHRQELIQDLFKEWKDPQKGVVTG
Homo sapiens         PAG----DGKKPSITAVVGSMDAH-PSRYCATVRVQRPRQEIIEDLS-------------
Drosophila AGO-1     PAG----DNKKPSIAAVVGSMDAH-PSRYAATVRVQQHRQEIIQELS-------------
Emiliania|414846|    GRG-----STADSHPAICYSLDRL-CSKWATEVTSQKAKEELVP--AEWLK---------
Emiliania|226029|    APG-----SSRPSYGALVGTVDKF-FSSHYTEVRAMTGRQECI--PVDDMQ---------
Emiliania|46005|     ------------------------------------VQRQEIVGSLEESMK---------

Chlamy AGO           ---RDIVV--------SMRESVVLMYRDGLSESQFDRALAEEFTAIVQACADVGGPSYRP
Drosophila AGO-2     SITLEH-L-RVYKEYRNAYPDHIIYYRDGVSDGQFPKIKNEELRCIKQACDKV---GCKP
Chlamy AGO-like      GATKELLL-EFYRANKQVKPQRLVMYRDGVSEGQFEQVLAEEYTALRRACRELE-EGYRP
Nannochloropsis      NMVQKLLL-KFHKTT-GSLPHKLIFYRDGVSEGQFKEVLQYEVPAVKSACSRIN-SKYEP
Arabidopsis          GMIKELLI-AFRRST-GHKPLRIIFYRDGVSEGQFYQVLLYELDAIRKACASLE-AGYQP
Homo sapiens         YMVRELLI-QFYKST-RFKPTRIIFYRDGVPEGQLPQILHYELLAIRDACIKLE-KDYQP
Drosophila AGO-1     SMVRELLI-MFYKSTGGYKPHRIILYRDGVSEGQFPHVLQHELTAIREACIKLE-PEYRP
Emiliania |414846|   ARMV-HAAQEFQKRT-GYTVRRLFFYRDGLAHNMFSI---------------------CP
Emiliania |226029|   SMVREQ-LKAFYRYN-GIPPQRIICYRDGVAHNQFEEVHEVEIHAIERACHAI-EQGYQP
Emiliania |46005|    SLFRDFVSADCWRTG-GRPPKRIIFFRDGVAHNQFEEVTRQEIAQIRRAADAICGEAYKP
                                           :. :***: .  :                        *

Chlamy AGO           RICFVVVQKSHNTRFFPTSAES--------THKSGNVLPGTAVDRGVTDP----HAFDFF
Drosophila AGO-2     KICCVIVVKRHHTRFFPSGDVTT-------SNKFNNVDPGTVVDRTIVHP----NEMQFF
Chlamy AGO-like      AITFVVVQKRHNTRLLPSDRAA--------SDQKGNVVPGTVVDSGITAP----DGFDFY
Nannochloropsis      SLTFIVVQKRHNTRFNGVERAEQ-------DGKSGNLKSGLVVDAGVCHP----FEYDFF
Arabidopsis          PVTFVVVQKRHHTRLFAQNHNDR-----HSVDRSGNILPGTVVDSKICHP----TEFDFY
Homo sapiens         GITYIVVQKRHHTRLFCADKNER-------IGKSGNIPAGTTVDTNITHP----FEFDFY
Drosophila AGO-1     GITFIVVQKRHHTRLFCAEKKEQ-------SGKSGNIPAGTTVDVGITHP----TEFDFY
Emiliania|414846|    ELVYIVVQKRTRARFCMRTPDG---------RRYEKPPYGLVVDREVTDATHHTAAGDFY
Emiliania|226029|    QIVFIVEQKGGGARFFANENGRVPGQQ------MGNQPPLTVVDTVVVDE----STFSFH
Emiliania|46005|     QLIFIVTQMRTKARFAIQGVAPSKGPPAPGVEDWLNVSSGTVVDRDVTGK----DAFDWY
                      :  ::      :*:                    :     .**  :          .:.

Chlamy AGO           LNSHAGIQGT----NRAPRYTVLVDEVGFTAEALQLLTHTLCHTYPACTRALSLPPPVRY
Drosophila AGO-2     MVSHQAIQGT----AKPTRYNVIENTGNLDIDLLQQLTYNLCHMFPRCNRSVSYPAPAYL
Chlamy AGO-like      LNSHAGLQGT----NKPAHYHVLVDEIGFGADGIQLLTYWLCYLYQRTTKSVSYCPPAYY
Nannochloropsis      LLSHAGLKGT----SKPSYYRVLRDESKMGPDELQQLTYFLCHNYARCTRSVSMVPAVYY
```

```
Arabidopsis          LCSHAGIQGT----SRPAHYHVLWDENNFTADGLQSLTNNLCYTYARCTRSVSIVPPAYY
Homo sapiens         LCSHAGIQGT----SRPSHYYVLWDDNRFTADELQILTYQLCHTYVRCTRSVSIPAPAYY
Drosophila AGO-1     LCSHQGIQGT----SRPSHYHVLWDDNHFDSDELQCLTYQLCHTYVRCTRSVSIPAPAYY
Emiliania|414846|    LSPHFALKGRTPRTPRPAHYHVIVDEANLTADQVHDFTFELCHVYQRATKVVSCPAPVYY
Emiliania|226029|    LTPHHGMKGT----SRGNLYSVLRNDPNLSSDDLQRFTSQLCHLFQRCKKIVSKVAPNYN
Emiliania|46005|     MVPHHALQGT----ARASHYHVLSNDAKLSADKLQRFTFDLCHVYGRATKIVSRPAHVYY
                     :  * .::*      :   * *: :   :  : :: :*  **: :   .: :*


Chlamy AGO           ADRAADRART--
Drosophila AGO-2     AHLVAARGRV--
Chlamy AGO-like      ADRAAFRGRTLL
Nannochloropsis      SHLAAKRAQ---
Arabidopsis          AHLAAFRARFYM
Homo sapiens         ARLVAFRAR---
Drosophila AGO-1     AHLVAFRARYHL
Emiliania|414846|    AHLAAFQAQYN-
Emiliania|226029|    AHLAAEM-----
Emiliania|46005|     AHLAAFNGP---
                     :  .*
```

S11 Fig. ClustalW multiple sequence alignment of the PIWI domain of the *E. huxleyi* argonaute paralogs (226029; 46005; 414846), together with argonaute and argonaute-like homologs from *Homo sapiens* (NP_036331.1)*, Drosophila melanogaster* (NP_730054.1; NP_725341.1)*, Arabidopsis thaliana* (*NP*_849784.1)*, Nannochloropsis gaditana* (EWM22342.1) and *Chlamydomonas* (EDO99188.1; EDP01993.1). Invariant residues are indicated by asterisks; highly residues by semicolon; and homologous residues by a period. Catalytic residues of the PIWI domain are highlighted in red.
