# Supplementary material for: Characterization of the Small RNA Transcriptome of the Marine Coccolithophorid, Emiliania huxleyi
Source: PLoS One. 2016 Apr 21;11(4):e0154279. doi: 10.1371/journal.pone.0154279 (PMC4839659; doi:10.1371/journal.pone.0154279)
Supplement: S12 Fig — Alignments of protein sequences were made with Muscle and the tree built with PhyML3.0. The sequences used in this alignment are Thalassiosira pseudonana (Tp) Tp_Ago (JGI ProtID1029); Phaeodactylum tricornutum (Pt) Pt_Ago (JGI ProtID47611); Drosophila melanogaster (Dm) Aubergine (CAA64320), PIWI (NP_476875), Ago1 (NP_725341), Ago 2 (NP_730054), Ago3 (ABO27430); Homo sapiens (Hs) Hili (NP_060538), Hiwi (NP_004755), Hiwi 2 (NP_689644), Hiwi 3 (NP_001008496), Ago1 (NP_036331), Ago2 (NP_036286), Ago3 (NP_079128), Ago4 (NP_060099); Chlamydomonas reinhardtii (Cr) (XP_001694841.1); Micromonas sp. RCC299 (MRCC299) (ACO60725.1); Shizosaccharomyces pombe (Sp) Ago (NP_587782); Ectocarpus siliculosus (Es) Ago1 (CBJ30598.1); Emiliania huxleyi (Eh) (JGI ProtID226029); Caenorhabiditis elegans (Ce) Alg1 (NP_510322), Alg2 (NP_871992); Arabidopsis thaliana (At) Ago1 (NP_849784), Ago2 (NP_174413), Ago3 (NP_174414), Ago4 (NP_565633), Ago5 (At2g27880), Ago6 (At2g32940), Ago7 (NP_177103), Ago8 (NP_197602), Ago9 (CAD66636), Ago10 (NP_199194). Accession numbers are for GenBank and genome portals (Tp, Pt, Eh) at the Joint Genome Institute (JGI), US. (PDF) [file pone.0154279.s012.pdf]

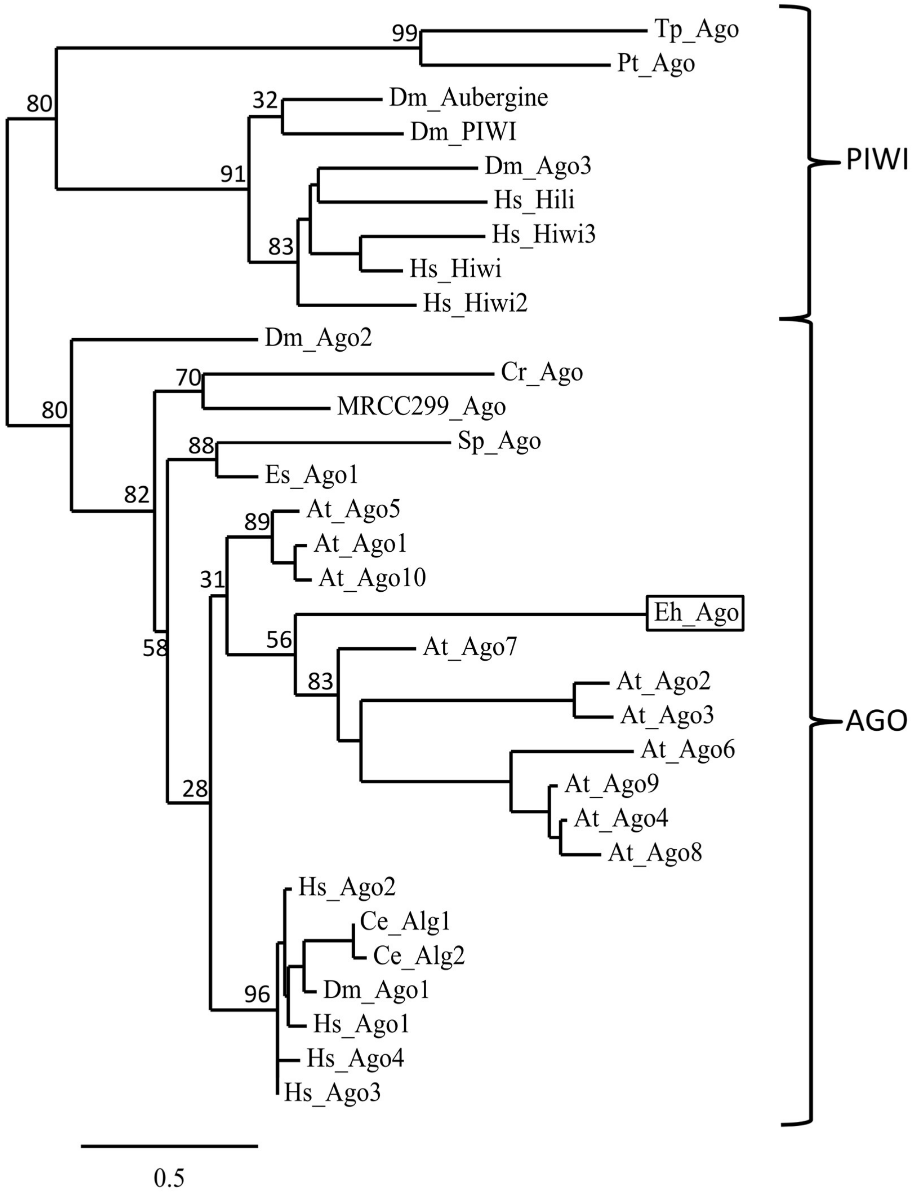

S12 Fig. Maximum Likelihood tree based on eukaryotic AGO proteins from the PIWI and AGO subfamilies. Alignments of protein sequences were made with Muscle and the tree built with PhyML3.0. The sequences used in this alignment are Thalassiosira pseudonana (Tp) Tp_Ago (JGI ProtID1029); Phaeodactylum tricornutum (Pt) Pt_Ago (JGI ProtID47611); Drosophila melanogaster (Dm) Aubergine (CAA64320), PIWI (NP_476875), Ago1 (NP_725341), Ago 2 (NP_730054), Ago3 (ABO27430); Homo sapiens (Hs) Hili (NP_060538), Hiwi (NP_004755), Hiwi 2 (NP_689644), Hiwi 3 (NP_001008496), Ago1 (NP_036331), Ago2 (NP_036286), Ago3 (NP_079128), Ago4 (NP_060099); Chlamydomonas reinhardtii (Cr) (XP_001694841.1); Micromonas sp. RCC299 (MRCC299) (ACO60725.1); Shizosaccharomyces pombe (Sp) Ago (NP_587782); Ectocarpus siliculosus (Es) Ago1 (CBJ30598.1); Emiliania huxleyi (Eh) (JGI ProtID226029); Caenorhabiditis elegans (Ce) Alg1 (NP_510322), Alg2 (NP_871992); Arabidopsis thaliana (At) Ago1 (NP_849784), Ago2 (NP_174413), Ago3 (NP_174414), Ago4 (NP_565633), Ago5 (At2g27880), Ago6 (At2g32940), Ago7 (NP_177103), Ago8 (NP_197602), Ago9 (CAD66636), Ago10 (NP_199194). Accession numbers are for GenBank and genome portals (Tp, Pt, Eh) at the Joint Genome Institute (JGI), US.
