# Supplementary material for: Characterization of the Small RNA Transcriptome of the Marine Coccolithophorid, Emiliania huxleyi
Source: PLoS One. 2016 Apr 21;11(4):e0154279. doi: 10.1371/journal.pone.0154279 (PMC4839659; doi:10.1371/journal.pone.0154279)
Supplement: S13 Fig — Alignments of protein sequences were made with Muscle and the tree built with PhyML3.0. Gene identifiers are given in the tree and are either for GeneBank or the Emiliania huxleyi genome portal at the Joint Genome Institute (JGI), US. (PDF) [file pone.0154279.s013.pdf]

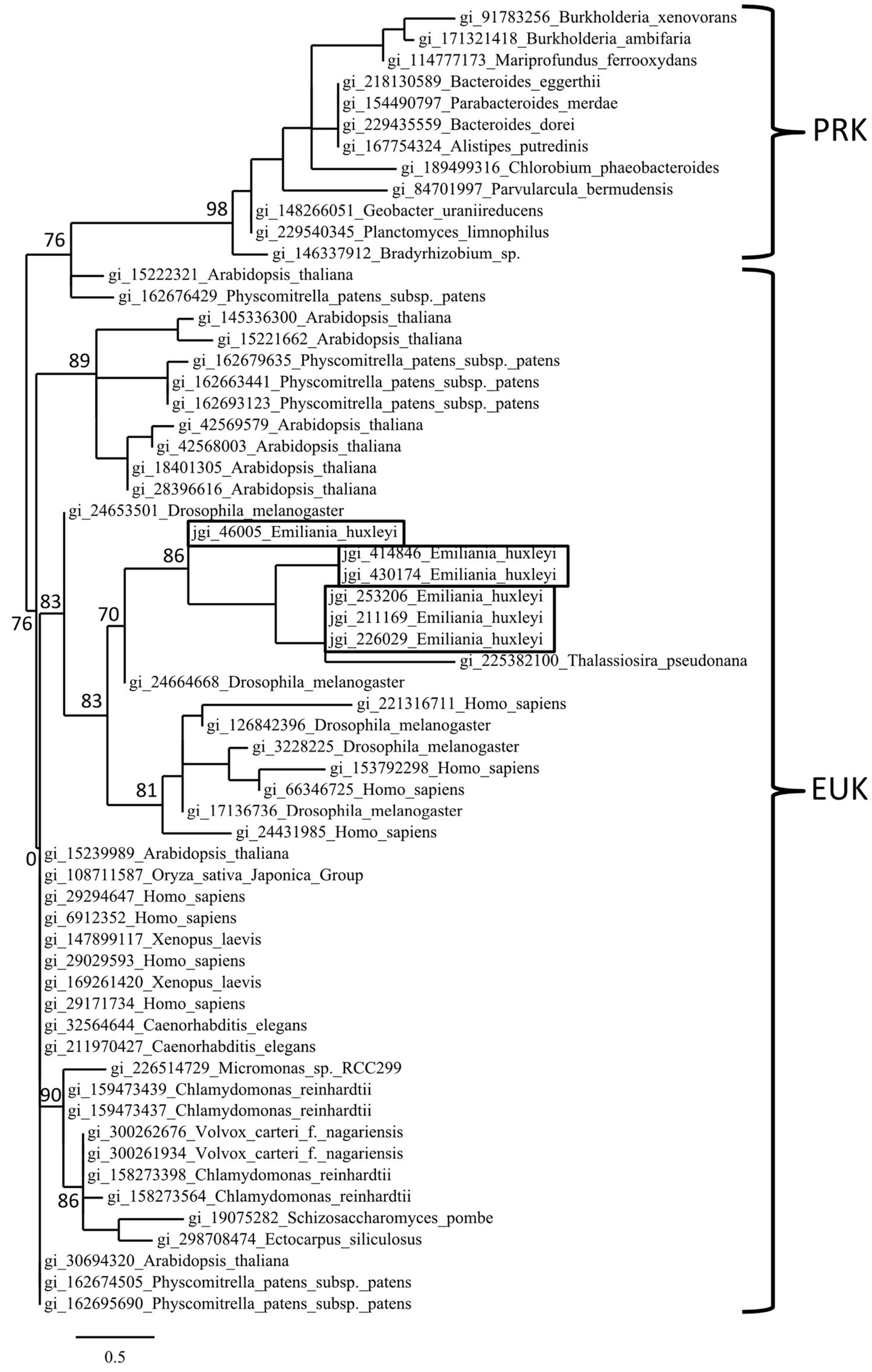

S13 Fig. Maximum Likelihood tree based on eukaryotic (EUK) and prokaryotic (PRK) PIWI domain-containing AGO-like proteins. Alignments of protein sequences were made with Muscle and the tree built with PhyML3.0. Gene identifiers are given in the tree and are either for GeneBank or the Emiliania huxleyi genome portal at the Joint Genome Institute (JGI), US.
