# Supplementary material for: Characterization of the Small RNA Transcriptome of the Marine Coccolithophorid, Emiliania huxleyi
Source: PLoS One. 2016 Apr 21;11(4):e0154279. doi: 10.1371/journal.pone.0154279 (PMC4839659; doi:10.1371/journal.pone.0154279)
Supplement: S14 Fig — elegans RRF-1, S. pombe Rdp1, A thaliana Rdp, N. gruberi RdP, and L. corymbifera RdP. Invariant residues are marked with an asterisk; colon, conserved residues. The aspartic acid residue that is essential for RNA dependent RNA polymerase activity is highlighted in red. (PDF) [file pone.0154279.s014.pdf]

```
L. corymbifera CDH59736.1           DIPSMCSGGDLDGDDYTIFWDQDLIPKVKN-YDPMEY----------KAD 863
N. gruberi XP_002670029.            PITNMITGSDLDGDQFFVYWDPDLIPESD--SEPMDYSVGAVAKPTNKKD 352
Arabidopsis Rdp NP_172932.1         PHPNECSGSDLDGDIYFVCWDQELVPPRTS--EPMDYTP---------EP 826
S. pombe Rdp 1 CAB11093.1           SIPAMCSGGDLDGDEYTVIWDQRLLPKIVNYPPLLESSP--------KKS 931
E. huxleyi RdRP 205162              PLSDQSAGGDLDGDKYLVCWDADIAAARQE----REAGR----------- 254
C. elegans EGO-1AAF80367.1          PHPDEMAGSDLDGDEYSIIWDQQLLLDKNE--DPYDFTS--------EKQ 1004
                                      .   :*.***** : : **  :
```

S14 Fig. Multiple sequence alignment of the catalytic domain of *E. huxleyi* RdP and its homologs from *C. elegans* RRF-1, *S. pombe* Rdp1, *A thaliana* Rdp, *N. gruberi* RdP, and *L. corymbifera* RdP. Invariant residues are marked with an asterisk; colon, conserved residues. The aspartic acid residue that is essential for RNA dependent RNA polymerase activity is highlighted in red.
