# Supplementary material for: Characterization of the Small RNA Transcriptome of the Marine Coccolithophorid, Emiliania huxleyi
Source: PLoS One. 2016 Apr 21;11(4):e0154279. doi: 10.1371/journal.pone.0154279 (PMC4839659; doi:10.1371/journal.pone.0154279)
Supplement: S15 Fig — Invariant residues are indicated by asterisks while highly and moderately homologous amino acid residues are indicated by “:” and “.”, respectively. Eight essential active site residues that contact the methyl donor, metal ions, and the RNA methyl acceptor are highlighted in red. (PDF) [file pone.0154279.s015.pdf]

```
Emihu1|454426|                      MLLDARGCCDYEVAEWVGDAVLRFLSVAHARDKQLSLAAEQILRNQSLYE 50
Arabidopsis                         --------------------------------------------------


Emihu1|454426|                      RAKESRPRALAAANLVESLLGGLAETARQQALKDGQADPLRRALEAASRL 100
Arabidopsis                         -MAGGGKHTPTPKAIIHQKFGAKASYTVEEVHDSSQSGCLGLAIQKGPCL 49
                                        .  :: :.  ::.. :*. *. : ::. ...*:. *  *:: .. *

Emihu1|454426|                      FRAAVVQLNEKMPPPQRRGRQPPPDKQLQQQLQEQAAALAAVSLELVARY 150
Arabidopsis                         YRCHLQLPEFSVVS------------NVFKKKKDSEQSAAELALDKLGIR 87
                                    :*. :   : .: .            :: :: ::.  : * ::*: :.

Emihu1|454426|                      PECDEGELTHLRSCLVKNLHLSRLLTRRFGAALAREFVRDASEAHRRAIE 200
Arabidopsis                         PQNDDLTVDEARDEIVG----------RIKYIFSDEFLS----------- 116
                                    *: *:  : . *. :*           *:   :: **:

Emihu1|454426|                      SFVAGVDTTSGLSMLEAPLLTPRGPTARPLGDQYEAIVGLVLTALHGDVD 250
Arabidopsis                         --------------AEHPLGAHLRAALRRDGERCGSVPVSVIATVDAKIN 152
                                                  * ** :   .: *  *::  ::   *::::...::

Emihu1|454426|                      RTWAEASEVSCMEAEAAAAPQQRAAHGGKRPLEGEAHGGKRPLEGEAHGG 300
Arabidopsis                         SRCKIINPSVESDPFLAISYVMKAAAKLADYIVASPHGLRR---KNAYPS 199
                                          .     :.  * :   :**      : ...** :*    :*: .

Emihu1|454426|                      KRPLEGEAHGGKRPLEGEAHGGKRPLEGEAHGGKRPLEAEAEQPRLCKRC 350
Arabidopsis                         EIVEALATHVSDSLHSREVAAVYIPCIDEEVVELDTLYISSNRHYLDSIA 249
                                    :      :* ..   . *. .   *  .*      .*  .:::  * . .

Emihu1|454426|                      KRDGGIEPSRFLRAEESLASPFWCVVRVDGTGEALGRSGGHSKKKEAKKA 400
Arabidopsis                         ERLG------LKDGNQVMISRMFGKASCG----SECRLYSEIPKKYLDNS 289
                                    :* *      :  .:: : * ::  .  .    :  *  ..  **  .::

Emihu1|454426|                      AYHDALSSTRLRELLQERRAAGLRQFALRLPVCDGGRALGVELGQGGVEA 450
Arabidopsis                         SDASGTSNEDSSHIVKSRNAR-------------ASYICGQDIHGDAILA 326
                                    :  .. *.    .:::.*.*               ..   * ::  ..: *

Emihu1|454426|                      ALRGEGIFEELGEGEGGMQRKVAAVAGTARLGLRSLSLLARKAAELSRRG 500
Arabidopsis                         SVGYRWKSDDLDYDDVTVNSFYRICCGMSPNGIYKISRQAVIAAQLP--- 373
                                    ::  .   ::*. .:  ::      .* :  *: .:*  *  **:*.

Emihu1|454426|                      PEGVGRVGEVVEAARDPASLRAGLRKLNWGALKAEAEADGLAEASAADPK 550
Arabidopsis                         -----------FAFTTKSNWRGPLPREILGLFCHQHRLAEPILSSSTAPV 412
                                                *    :. *. * :   * :  : .      :*:: *

Emihu1|454426|                      DELIRLLVARRSRVGVRLATGARKAVPRCNLQLLCLGHCHLGGMSEPARL 600
Arabidopsis                         KSLSDIFRSHK-KLKVSGVDDANENLSRQKEDTPGLGHGFRCEVKIFTKS 461
                                    ..*  :: ::: :: *  . .*.: :.* : :   *** .   :.  ::

Emihu1|454426|                      LAVWLQMAGGDPCALTRWQSNGVFDPALFRRERAAGEGGEGEGAPPPSLS 650
Arabidopsis                         QDLVLECSPRK----------------FYEKENDAIQN----ASLKALLW 491
                                      : *: :  .                ::.:*. * :.    .:  . *

Emihu1|454426|                      SAMLLPWAAEEEAPRVVALPRRIDYLNVISEALGGGGALGVHARCEGQADL 700
Arabidopsis                         FSKFFADLDVDGEQSCDTDDDQDTKSSSPNVFAAPPILQKEHSSESKNTN 541
                                     : ::.    :. :         *  .  .:.:..   *  .  .*.:

Emihu1|454426|                      GRAQLSLRCGGAALYESFLAQHGAGLLGFVVGCTGAPLLGQWALPRFEAW 750
Arabidopsis                         -----------VLSAEKRVQSITNGSVVSICYSLSLAVDPEYSSDGESPR 580
                                               .   *. : .   * :  :  . . .:  :::    ..

Emihu1|454426|                      PSERPPQTVLQEYLERRGCWCRGAWAGEGGVVELRGPPCRSKREAERSVA 800
Arabidopsis                         EDNESNEEMESEYSAN-------------------CESSVELIESNEEIE 611
                                     .:.. : : .** .                     .. .  *::..:
```

```
Emihu1|454426|                      YALLQAALPQAADSPPPPLLVESVSYTLQLEPDGSGGVGATLEVQRRLCV 850
Arabidopsis                         FEVGTGSMNPHIESEVTQMTVGEYASFRMTPP----------DAAEALIL 651
                                    : :  .::    :*  . : * . :      *          :. . * :

Emihu1|454426|                      LLGGGLLHPEAALRVVYRGKRRRCRLWLQLHSATAAEEFECVAARDGGPS 900
Arabidopsis                         AVG------SDTVRIRSLLSERPCLNYNILLLGVKGPSEERMEAAFFKPP 695
                                     :*      . ::*:    ..* *  :  *  .. . . * : *    *.

Emihu1|454426|                      LGALRINYVCELLRQLSPASLCDVGCGEGRAGTAALVGEGRLICSLLRGG 950
Arabidopsis                         LSKQRVEYALKHIRESSASTLVDFGCGSG-----------SLLDSLLDYP 734
                                    *.  *::*. : :*: *.::* *.***.*            *: ***

jgi|Emihu1|454426|estExtDG_Gen      GAPSLRRVVANDVSARALRRCGEKVRAALAARAGGGAPSAELEFELLCSP 1000
Arabidopsis                         --TSLQTIIGVDISPKGLARAAKMLHVKLNKEACNVKSATLYDGSILEFD 782
                                      .**: ::. *:*.:.* *..: ::. *  .* .  .::  : .:*

Emihu1|454426|                      VSELRLAAGCDVLTLVEVVEHLDPPELQRLGAALLGRCAPRALIVTTPNK 1050
Arabidopsis                         S----RLHDVDIGTCLEVIEHMEEDQACEFGEKVLSLFHPKLLIVSTPNY 828
                                            . *: * :**:**::  :  .:*  :*.   *: ***:***

Emihu1|454426|                      EYNLNLMVTCSEEARGGAACRGRGGAGLTLDEVRARSRRGQLCKGCCTYT 1100
Arabidopsis                         EFNTILQRSTPETQEE---------------------------------- 844
                                    *:*  *  : .*  .

jgi|Emihu1|454426|estExtDG_Gen      SLLSGAPPPPHAHYPLRNKDHRFEWTRAEFRAWAEGLAAEHGYSVRFDGV 1150
Arabidopsis                         --NNSEPQLPK----FRNHDHKFEWTREQFNQWASKLGKRHNYSVEFSGV 888
                                       .. *  *:    :**:**:***** :*. **. *. .*.***.*.**

Emihu1|454426|                      GG------------GAWDEERQPGAPCHGPGPSSQVAIFERPAGLREQM- 1187
Arabidopsis                         GGSGEVEPGFASQIAIFRREASSVENVAESSMQPYKVIWEWKKEDVEKKK 938
                                    **              . : .* ..      .. ..  .*:*      *:

Emihu1|454426|                      ---
Arabidopsis                         TDL 941
```

S15 Fig. Clustalw (2.1) multiple sequence alignment of HEN1 methyltransferase of *Arabidopsis thaliania* (AT4G20910) and *E. Huxleyi* (454426). Invariant residues are indicated by asterisks while highly and moderately homologous amino acid residues are indicated by ":" and ".", respectively. Eight essential active site residues that contact the methyl donor, metal ions, and the RNA methyl acceptor are highlighted in red.
