# Supplementary material for: Characterization of the Small RNA Transcriptome of the Marine Coccolithophorid, Emiliania huxleyi
Source: PLoS One. 2016 Apr 21;11(4):e0154279. doi: 10.1371/journal.pone.0154279 (PMC4839659; doi:10.1371/journal.pone.0154279)
Supplement: S16 Fig — Considerable sequence divergence is noted in the N-terminus. The C-terminus is more highly conserved and contains the ATPase and the MOV-10 helicase domains. While the homologs from E. huxleyi share between 30–40% amino acid identity,they share 20–30% identity with homologs from Arabidopsis and Drosophila. Conserved helicase motifs are highlighted in red. (PDF) [file pone.0154279.s016.pdf]

```
jgi|Emihu1|247007|gm1.54200015      --------------------------------------MKLDTLSADLSP 12
jgi|Emihu1|119956|fgeneshEH_pg      MAFTYCCDVAATSAAVEAIVNSSAALCLDLEWDSHDPNTPLSLIQAATGT 50
jgi|Emihu1|436918|estExtDG_fge      --------------------------------------------------
Arabidopsis                         --------------------------------------------------
Drosophila                          --------------------------------------MFTYVSKFFTNP 12

jgi|Emihu1|247007|gm1.54200015      ARGG-------------------DKVQLTSLGKVPKPHG----------- 32
jgi|Emihu1|119956|fgeneshEH_pg      GTGTGTGQPFLLDVVRVPSAAAVNVSRMSSLGEIIASQHPVAMHAAAQDR 100
jgi|Emihu1|436918|estExtDG_fge      --------------------------------------------------
Arabidopsis                         --------------------------------------------------
Drosophila                          DRNREDILESLDRENSFLDQKLMEEKMDQQLKANPNEINGVLSNKIAELT 62

jgi|Emihu1|247007|gm1.54200015      --LQRK------------------------------------------LK 38
jgi|Emihu1|119956|fgeneshEH_pg      LVLQRHGIALVNLFDTQIAHELLTGARRKSLRDVLQHWLGIDISKGGELM 150
jgi|Emihu1|436918|estExtDG_fge      --------------------------------------------------
Arabidopsis                         --------------------------------------------------
Drosophila                          HGLSEMDVSKESSCTARKGVITSLDGDRGVIDKDVLFETKVAEDIILDLH 112

jgi|Emihu1|247007|gm1.54200015      SVIKQHPAVFT-------------------------LYEGAVSGGAETVS 63
jgi|Emihu1|119956|fgeneshEH_pg      RRFMQTPHAWTSRPLPDYVLDYAAEDVRHLPQLYATMKAEAERRGADVLD 200
jgi|Emihu1|436918|estExtDG_fge      --------------------------------------------------
Arabidopsis                         --------------------------------------------------
Drosophila                          VGCVVEYLTFTTGEAMRVVKVKSILEHSWEDTSQKEIEKAVDNLKNEKPT 162

jgi|Emihu1|247007|gm1.54200015      LVVTPS-------------------------------------------- 69
jgi|Emihu1|119956|fgeneshEH_pg      QILVLSRGDRVSAAAASAAKYTATIKWFDRKKGYGFASPEGGGDDLFVHA 250
jgi|Emihu1|436918|estExtDG_fge      --------------------------------------------------
Arabidopsis                         --------------------------------------------------
Drosophila                          FFNTETR------------------------------------------- 169

jgi|Emihu1|247007|gm1.54200015      --------------------------------------------------
jgi|Emihu1|119956|fgeneshEH_pg      LNFAKEKGNKQPFVDDGDIIYYDLGEYNGRPTAVNVTFPADRPARSRRRR 300
jgi|Emihu1|436918|estExtDG_fge      --------------------------------------------------
Arabidopsis                         --------------------------------------------------
Drosophila                          --------------------------------------------------

jgi|Emihu1|247007|gm1.54200015      ------------PPAAVPPQPASPSSQGPP---------------VAATE 92
jgi|Emihu1|119956|fgeneshEH_pg      FVAREQQQIPQPQQTPVPLQEDSPPALAPVPAVARIAAKGKGKGAAAAVA 350
jgi|Emihu1|436918|estExtDG_fge      --------------------------------------------------
Arabidopsis                         --------------------------------------------------
Drosophila                          -----------SVLGLISQRLASSIDVETEYGQLTVELDNIEMNFIPTNG 208

jgi|Emihu1|247007|gm1.54200015      EHTVIAFGQQLRADDALRARCAKKDAFKAEYAAWKARELTDGR-KWRGAA 141
jgi|Emihu1|119956|fgeneshEH_pg      RETALAFLAFLDANVEVRAECEDKDAFRRRLAAWRAEEAAAGRQRQRHKA 400
jgi|Emihu1|436918|estExtDG_fge      --------------------------------------------------
Arabidopsis                         ------MSVSGYKSDDEYSVIADKGEIGFIDYQNDGSSGCYNPFDEGPVV 44
Drosophila                          DRVRLECNIQLDDGFVDKQGEILEVTKLFPTRIQEGEKCIVERVYVHMVV 258

jgi|Emihu1|247007|gm1.54200015      ATVLFRLWRFSAAQGARLGVVRIKAVVGQRVYFAAIGAMAALAESVVEQR 191
jgi|Emihu1|119956|fgeneshEH_pg      QDVLFQLLRAGKAAVAGS-TIAFLAHGGKR---AKQEALAASAGRVEANR 446
jgi|Emihu1|436918|estExtDG_fge      --------------------------------------------------
Arabidopsis                         VSVPFPFKKE-------------------------------KPQSVTVGE 63
Drosophila                          LGPETYILKTDLPTGTDLHLGDIVLADLIECQYSKFTRRAIKITPLEKNF 308
```

```
jgi|Emihu1|247007|gm1.54200015      DVMEKKDKFGVRVTP---LHVFEGGLIVRRGAAVQRTLVVEN-ASDEMRE 237
jgi|Emihu1|119956|fgeneshEH_pg      STIEA-DKGGVAVSG---VALPET---VRPGETATCQVAVRNHGAGAALV 489
jgi|Emihu1|436918|estExtDG_fge      --------------------------------------------------
Arabidopsis                         TSFDSFTVKNTMDEP---VDLWTKIYASNPEDSFTLSILKPPSKDSDLKE 110
Drosophila                          GATKLTQQSSMGSSGSGKAVTVTGVNRFITAELWQKESVSLKLTNNLNRT 358

jgi|Emihu1|247007|gm1.54200015      LAGASFLQRGPGPFSSSFTGEDGALVLTPGGKVTLTLTCRPILSGMCNNV 287
jgi|Emihu1|119956|fgeneshEH_pg      LRRVEVLRRTTG-FACQPCG-ELDVRLPPGAEVTLVIECSPRHVGMCYET 537
jgi|Emihu1|436918|estExtDG_fge      --------------------------------------------------
Arabidopsis                         RQ------------CFYETFTLEDRMLEPGDTLTIWVSCKPKDIGLHTTV 148
Drosophila                          LRLESITVCNDSESQLSVVSPLESKEISSGSEITVTFEIHTQFLGEAIEK 408

jgi|Emihu1|247007|gm1.54200015      LNLNFG----HFGIARFLEA------------------------PPLGSS 309
jgi|Emihu1|119956|fgeneshEH_pg      LSITFDDEGARFTVGRFLSVSCGDPDVLDVLKATAPYQRPKRRRPPPAKE 587
jgi|Emihu1|436918|estExtDG_fge      --------------------------------------------------
Arabidopsis                         VTVDWGSDRVERVVFLLAEDKISSS------------------LTSNRPY 180
Drosophila                          YVLNFDLLKVRRVFTVIVCKTKEEVAEAEKR----MIAAEALMAPGRNSQ 454

jgi|Emihu1|247007|gm1.54200015      SFGGAAANTRRAQAGKLPLYRIPGSLRAAVG-SGEAGEALEAACEQM--- 355
jgi|Emihu1|119956|fgeneshEH_pg      QVTEIAPPSEPPSSVKLKLPMQAFGIKPEWRRKMESGSAIEELEGLR--- 634
jgi|Emihu1|436918|estExtDG_fge      --------------------------------------------------
Arabidopsis                         SRSRRAPKKDFAVDDYVKGSRPSKVVERSFRNRLPLYEIPKEIREMIEN- 229
Drosophila                          ERSRFYANQVWCNKVDVIPGQQIVTKRRFVALRLGCFEVPKELRQICLTS 504

jgi|Emihu1|247007|gm1.54200015      ----------KRLLGGVSEAAALAAYKKAFHELLWVEEMQLNDDLREFDL 395
jgi|Emihu1|119956|fgeneshEH_pg      ----------ARLMS-EQSAGLLSLYADLFAKLLWLEERQLVADLSNFDL 673
jgi|Emihu1|436918|estExtDG_fge      --------------------------------------------------
Arabidopsis                         ----------KEFPDDLNEGLTARNYANYYKTLLIMEELQLEEDMRAYDM 269
Drosophila                          ERRQEMIKAIEQHYSFLKEPLSVKTYMHRFRLLLHLEEIECFVNFRNYDR 554

jgi|Emihu1|247007|gm1.54200015      RADDATVLTPRGR-LFALEVRGLAENRPSVLKGDVIKAN---FPGDP--G 439
jgi|Emihu1|119956|fgeneshEH_pg      IEEKAVTLDPRGGGLLAVHVHGLAEKRPSVLKGDTLRVN---RVGEP--K 718
jgi|Emihu1|436918|estExtDG_fge      --------------------------------------------------
Arabidopsis                         EN----VSMKRRGIYLSLEVPGLAERRPSLVHGDFIFVR---HAYDDGTD 312
Drosophila                          DR----AHFLRDGEFLTLQIENLAERRPSLVIGDTLRVINPWSDPDSQTT 600

jgi|Emihu1|247007|gm1.54200015      RVFEGRAREIERETVLLEFNNTRFQYVAG-QRIEISFVLSRTPLRRFHQG 488
jgi|Emihu1|119956|fgeneshEH_pg      VVYLGRAALIEREDVLLRLK-PQLGYICG-QKVEVRFVMNRGPLRIFHQG 766
jgi|Emihu1|436918|estExtDG_fge      --------------------------------------------------
Arabidopsis                         HAYQGFVHRVEADEVHMKFASEFHQRHTAGSVYNVRFTYNRINTRRLYQA 362
Drosophila                          KSYEGIIHKVLFDRILLKFHSSFQEKYNG-EDYRLEFYFSRYSFRKQHHA 649

jgi|Emihu1|247007|gm1.54200015      LDGVDRAG--TAGAASRLFPEMAHLATAQLHAARSVAAPLRPFDRRINA- 535
jgi|Emihu1|119956|fgeneshEH_pg      LSMLGKL------APHVLFPPNA-LAASLRKPPRTSTFPLRFSNRSVGAN 809
jgi|Emihu1|436918|estExtDG_fge      --------------------------------------------------
Arabidopsis                         VDAAEMLD--PNFLFPSLHSGKRMIKTKPFVPISPALN------------ 398
Drosophila                          ISKIVGVMGEDFLFPSKVTKRENPQLDVYMKDDDMYLYDSKLEWYNQSLN 699

jgi|Emihu1|247007|gm1.54200015      -QQLAAVTAIVEGRARHVPYIIYGPPGTGKTTTVVECVLQCVKR------ 578
jgi|Emihu1|119956|fgeneshEH_pg      EAQAEAVRRVVEGEARAVPYCIFGPPGTGKTTTVVELVLQCRKLPPSVAC 859
jgi|Emihu1|436918|estExtDG_fge      ------------MRPQLLKYACVSPETNQFGVPPLEQLLQQRLT------ 32
Arabidopsis                         -AEQICSIEMVLGCKGAPPYVIHGPPGTGKTMTLVEAIVQLYTT------ 441
Drosophila                          SIQKRAVFNILRGEAENIPYVLFGPPGSGKTMTLIETLLQLVRN------ 743
                                                       *   .*  .    . :* ::*
```

```
jgi|Emihu1|247007|gm1.54200015      -------VTGAELRVLVSSPTNTAADVLCKRLSEGG--LGSKMDMLRVNA 619
jgi|Emihu1|119956|fgeneshEH_pg      CGYTHTPPPAPAFRILVSAPTNTAADLLCSRLASKG--LRDPLSMLRLNS 907
jgi|Emihu1|436918|estExtDG_fge      ---------------------------LCTCAATHM--LLESGVPCASSL 53
Arabidopsis                         ---------QRNARVLVCAPSNSAADHILEKLLCLEGVRIKDNEIFRLNA 482
Drosophila                          ---------LPGARILVGTPSNSSADLVTKRLIDSK-ALLQGDFIRLVSY 783
                                                               :            .       .

jgi|Emihu1|247007|gm1.54200015      YTRNPNDIDERVLRHSNCAPAERTIEMPPKEELERV-------------- 655
jgi|Emihu1|119956|fgeneshEH_pg      YSRAKTDVPSDVFDLSCWSDGESAFTPPPLLELLSK-------------- 943
jgi|Emihu1|436918|estExtDG_fge      HGAGPHEPPAELLDADGAP------------------------------- 72
Arabidopsis                         ATRSYEEIKPEIIRFCFFD--ELIFKCPPLKALTRY-------------- 516
Drosophila                          NQVEKDLIPPEIMSYCATSDVGAVGSCEDKMMVTESGLKLRCQAKFIGTH 833
                                               ::

jgi|Emihu1|247007|gm1.54200015      RVVVATLTTAAKLCGEGIPRGHFDVIVIDEAGQAQEPESLAAASMLLGQG 705
jgi|Emihu1|119956|fgeneshEH_pg      SVVVATLSMAGKLVNFGVPRGHFDLLVIDEAGQALEPEAVAPIATLLGSD 993
jgi|Emihu1|436918|estExtDG_fge      --------------------LHYTHIIVDEASQALEPEMLLPLS-FAGPR 101
Arabidopsis                         KLVVSTYMSASLLNAEGVNRGHFTHILLDEAGQASEPENMIAVSNLCLTE 566
Drosophila                          RITISTCTTLGNFLQLGFPAGHFTHVLFDEAGQCTEPETMVPIVMLTKKR 883
                                                         *:  ::.***.*. *** : .   :

jgi|Emihu1|247007|gm1.54200015      GQLVLAGDPKQLGPVIHHSLAKEHGLSTSVLERLMERPIYQKHPLPGGFE 755
jgi|Emihu1|119956|fgeneshEH_pg      GQLVLAGDPRQLGPVIHDTRAQELGLATSLLERMMARPLYGRSDSG---- 1039
jgi|Emihu1|436918|estExtDG_fge      CDVLMCGDHRQLGPTVRSTYCREHGLATSMLERLMKLPLYAPPGDGASPP 151
Arabidopsis                         TVVVLAGDPRQLGPVIYSRDAESLGLGKSYLERLFECDYYCEGDEN---- 612
Drosophila                          SQVVLSGDPRQLQSIVTSRIALKMGFSISFLERLLERSPYRKDLQRFPES 933
                                      :::.** :** . :    . . *:. * ***::    *

jgi|Emihu1|247007|gm1.54200015      --EYDPRVLTKLLQNFRAHETLLELPNQLFYEGELLCRGDELLLKCCEGW 803
jgi|Emihu1|119956|fgeneshEH_pg      --DYNPLVLTKLVQNYRSHEVLLRLPNELFYEGELLPCADAELQRHCCGR 1087
jgi|Emihu1|436918|estExtDG_fge      PPSSGPPCVTKLVRNYRSHAALLSLPSRLYYGSELQECADPATSRVMEGW 201
Arabidopsis                         -------YVTKLVKNYRCHPEILDLPSKLFYDGELVASKE-DTDSVLASL 654
Drosophila                          S-GYNPLVLTKLLYNYRALPSIMSIYSRLFYDDELIPVLSEKDSRESRLL 982
                                            :***: *:*.   :: : ..*:* .**    .

jgi|Emihu1|247007|gm1.54200015      EG----LPSQGVP-----LLFDGIVGKDQQEERSPSWFNVDECHRVLEHV 844
jgi|Emihu1|119956|fgeneshEH_pg      EYSEEPLDTKGLP-----LIFDGVVGKDEREGSSPSWFNSHECQQVMRYV 1132
jgi|Emihu1|436918|estExtDG_fge      EG----LRRRGFP-----LLFYGVSSAHTHEVDSPSFFNPVEAEKVAELI 242
Arabidopsis                         NF----LPNKEFP-----MVFYGIQGCDEREGNNPSWFNRIEISKVIETI 695
Drosophila                          SKLRCVFESEKDIPQAHGTFFYGIIGENRQNNDSPSWFNPQEVREVFLMT 1032
                                    .     :  .         .* *: . . ::  .**:**  *  .*

jgi|Emihu1|247007|gm1.54200015      RDLLRAHNGCSVRLTPEDIGVIAPYNKQVQKLKRLFRAEQL----EGIKV 890
jgi|Emihu1|119956|fgeneshEH_pg      QALLRVRG---RPLAPADIGVITPYNRQAQKLSRLLAVAKLPVGKGGIKV 1179
jgi|Emihu1|436918|estExtDG_fge      ERLLAASKADGTGITTNDVAVVTPYRKQVVKVRQLLRSLGLG----AVRV 288
Arabidopsis                         KRLTANDC-----VQEEDIGVITPYRQQVMKIKEVLDRLDMT----EVKV 736
Drosophila                          IALYRANV------TADQIGIITPYQKQVKMLRSMFIGTDVVMP----KI 1072
                                      *              ::.:::**.:*.  :  ::    :       ::

jgi|Emihu1|247007|gm1.54200015      GSTEMFQGQEKRVIIISTVRSSPDWVG--CDVRHNLGFLDNPKRFNVAIT 938
jgi|Emihu1|119956|fgeneshEH_pg      GSTELFQGQERKVIILSTVRSSADQIG--FDVAHNLGFLQNPKRFNVATT 1227
jgi|Emihu1|436918|estExtDG_fge      GSVDDYQGQEETIIIISTVLASYGDRSSLGRLASPHSLMSSPQRFNVAIT 338
Arabidopsis                         GSVEQFQGQEKQVIIISTVRSTIKHNE--FDRAYCLGFLSNPRRFNVAIT 784
Drosophila                          GSVEEFQGQERDIILISTVRSSEEILR--MDARFSLGFVRCSKRLNVAVS 1120
                                    **.: :****. :*::*** ::              .::  .:*:*** :

jgi|Emihu1|247007|gm1.54200015      RAQALLIVVGNPNVLRLDFHWGSLLKHCVDNSAYRGVPLQPMPGAESDTV 988
jgi|Emihu1|119956|fgeneshEH_pg      RACCLMIVVGNPHVLATDPHWGHLLRLCVQLGAYRGVPAPTVT-AQTATT 1276
jgi|Emihu1|436918|estExtDG_fge      RARALLVVVGDPNALIDDPSWRELLRYAVENDSYRGCPHPLMMEGTEDEA 388
Arabidopsis                         RAISLLVIIGNPHIICKDMNWNKLLWRCVDNNAYQGCGLPEQEEFVEEPF 834
Drosophila                          RARAMMIIFGNPHLLAVDECWRQLILFCVKNNAYFGCDLPQMVINQKDEV 1170
                                    ** .::::.*:*: :  *  *  *:  .*. .:* *
```

```
jgi|Emihu1|247007|gm1.54200015      ATSLAG-----------------------DMEQLTLDDETP-----AELE 1010
jgi|Emihu1|119956|fgeneshEH_pg      GSARAGGSGGGGDGALTGDGGDGVEELTRQLEQLMLGADSSERVLQEGMA 1326
jgi|Emihu1|436918|estExtDG_fge      RLRTRWRSSPTTWRTRCWAAATRSPCSPPSRARRRTTARTMSRTTSPGAS 438
Arabidopsis                         KQEGSSNGPQYPPEAEWNNSGELNNGGANENGEWSDGWNNNGGTKEKNEW 884
Drosophila                          P---------------------------VCLETFVPSLNTTDDLN----- 1188
                                                                       .

jgi|Emihu1|247007|gm1.54200015      MPHYE--------------------------------------------- 1015
jgi|Emihu1|119956|fgeneshEH_pg      MPVWE--------------------------------------------- 1331
jgi|Emihu1|436918|estExtDG_fge      CCDPR--------------------------------------------- 443
Arabidopsis                         SDGWNSNGGGTKKKDEWSDGWDNNGGTNGINQEGSSNAPQDPQEAEWNDS 934
Drosophila                          --------------------------------------------------


jgi|Emihu1|247007|gm1.54200015      --------------------------------------------------
jgi|Emihu1|119956|fgeneshEH_pg      --------------------------------------------------
jgi|Emihu1|436918|estExtDG_fge      --------------------------------------------------
Arabidopsis                         GEVKNGGTKEKDVRSDGWNNNGGKNEKEECCDGWKDGGSGEEIKNGGKFE 984
Drosophila                          --------------------------------------------------


jgi|Emihu1|247007|gm1.54200015      ------------------
jgi|Emihu1|119956|fgeneshEH_pg      ------------------
jgi|Emihu1|436918|estExtDG_fge      ------------------
Arabidopsis                         TRGDFVAKEEDEWSDGWK 1002
Drosophila                          ------------------
```

S16 Fig. Multiple sequence analysis of the Armitage SDE3 protein from, *E. huxleyi* (247007;119956;436918), *Arabidopsis thaliana* (AEE27843.1) and *Drosophila melanogaster* (AAT12000.1). Considerable sequence divergence is noted in the N-terminus. The C-terminus is more highly conserved and contains the ATPase and the MOV-10 helicase domains. While the homologs from *E. huxleyi* share between 30-40% amino acid identity,they share 20-30% identity with homologs from *Arabidopsis* and *Drosophila*. Conserved helicase motifs are highlighted in red.
