# Supplementary material for: Characterization of the Small RNA Transcriptome of the Marine Coccolithophorid, Emiliania huxleyi
Source: PLoS One. 2016 Apr 21;11(4):e0154279. doi: 10.1371/journal.pone.0154279 (PMC4839659; doi:10.1371/journal.pone.0154279)
Supplement: S17 Fig — Key residues in the TUDOR domain include highly conserved Arginine and Aspartate residues in green; aromatic cage residues in red, responsible for complexing di-methylated guanine groups; Asparagine in purple, involved in the binding and interaction with PIWI proteins by means of the symmetric di-methylation of distinct arginine (sDMA) residues; and a strongly conserved Glycine in in blue. (PDF) [file pone.0154279.s017.pdf]

```
Arabidopsis 1       -------------MATG--AENQWLKGRVKAVTSGDCLVITALSHNRAGPPPEKTITFSS 45
Arabidopsis 2       -------------MATGAATENQWLKGRVKAVTSGDCLVITALTHNRAGPPPEKTITLSS 47
Populus             -------------MATSTAGATGWYRGKVKAVPSGDSLVIMAMTSSKPGPPPEKTITLSS 47
Emihu1|452958|      ----------MVEANGTSTAAARALPAIVKAVPSGDTVLVMGADSSR-GPPPEKLISLTG 49
Drosophila A        MATAANTATAAGAAKDAPPAPTKSLSGIVKQVLSGDTVVIR---ATKGAPPPEKQITFSH 57
Drosophila B        MATAANTATAAGAAKDAPPAPTKSLSGIVKQVLSGDTVVIR---ATKGAPPPEKQITFSH 57
Apis                --------------MSAPQGQMKPRNGVVKQVISGDTIVIRG--QPMGGPPPEVTITLCN 44
                                              . ** * *** :::        .****  *::

Arabidopsis 1       LMAPKMARRGG-------IDEPFAWESKEFLRKLCIGKEVAFKVDYKVEAIAG------- 91
Arabidopsis 2       LMAPKMARRGG-------IDEPFAWESREFLRKLCIGKEVAFKVDYKVEAIAG------- 93
Populus             LIAPRLARRGG-------VDEPFAWNSREYLRKLCIGKEVTFKVDYAVPSIG-------- 92
Emihu1|452958|      ITAPRLANRST-------DDSPWAWASRDFLRRLVIGKPVTFVIEAPASTVPGGPTGGPP 102
Drosophila A        VLAPKLARRPG-AGGDETKDEPWAWESREFLRKKLIGVEVTFTFDKPANSNREYG----F 112
Drosophila B        VLAPKLARRPG-AGGDETKDEPWAWESREFLRKKLIGVEVTFTFDKPANSNREYG----F 112
Apis                ITAPKLERWKGNDSTDESRDEPYSWEAREFLRKKLIGQDVAFVTEKSVNTNRTYG----T 100
                    : **:: .           *.*::* ::::**:  **  *:*  :  . :

Arabidopsis 1       REFGSVFLGNENLAKLVVKTGWAKVREPGQQNQDKVSPYIKELLQLEELAKQEGYGRWSK 151
Arabidopsis 2       REFGSVYLGNENLAKLVVQNGWAKVRRPGQQNQDKVSPYIAELEQLEEQAQQEGFGRWSK 153
Populus             REFGSVFLGEKNVALLVVSEGWAKVREQGQQ-KGEASPFLAELLRLEEQAKQQGLGRWSK 151
Emihu1|452958|      REFGTVYFEGLPLQQHIVAAGWARLKGVALQDEVLAP--------IEAASKEEGRGVWNE 154
Drosophila A        VWIGKDKETGENVVESIVREGLVSVRREGR-PTAEQQT----LIELEDQARAAGRGKWSP 167
Drosophila B        VWIGKDKETGENVVESIVREGLVSVRREGR-PTAEQQT----LIELEDQARAAGRGKWSP 167
Apis                VWLGKDK-NGENVIETLVSEGLVTVKKDTRNPSPEQTR----LIELENAAKAAKKGKWS- 154
                      :*.       :   :*  * . ::                   :*  ::    * *.

Arabidopsis 1       VPGAAEASIRNLPPSAIGDSAGFDAMGLLAANKGKPMEGIVEQVRDGSTIRVYLLPEFQF 211
Arabidopsis 2       VPGAAEASIRNLPPSAVGDSGNFDAMGLLAASKGKPMEGIVEQVRDGSTIRVYLLPEFQF 213
Populus             APGASEASIRNLPPSAIGDSSNFDAMGLLAANKGTPMECIVEQVRDGSTIRVYLLPDFQF 211
Emihu1|452958|      AS--AASSVR-----SIKWAGSFDAAGLVAAFGRQPQEAIIEHVPSGSFLRALLLPGFEQ 207
Drosophila A        TASAADKVRN-------IKWSHENPAHLVDIYGGNPVKAIIEHVRDGSTVRAFLLPDFHY 220
Drosophila B        TASAADKVRN-------IKWSHENPAHLVDIYGGNPVKAIIEHVRDGSTVRAFLLPDFHY 220
Apis                ESPNSEHIRD-------VKWTVDDPRKLVEKFGKKPVKAIIEFVFDGSTVKALLLPDFYN 207
                     .  :                  :.  *:      * : *:* * .** ::. *** *

Arabidopsis 1       VQVFVAGVQAPSMGRR-TTNGSVVET-VPDEPNGDVSAESRGPLTTAQRLAASAASSVEV 269
Arabidopsis 2       VQVFVAGLQAPSMGRRQSTQEAVVDPDVTATSNGDASAETRGPLTTAQRLAASAASSVEV 273
Populus             VQVFVAGIQAPSMGKR-AAIETVGETVTTSNGTNGDTSETRAPLTSAQRLAASAAP-PEV 269
Emihu1|452958|      VTLSLCGTQCP------------------------------PLKRGEDGVEEAAP---- 232
Drosophila A        ITLMISGIRCPGVKLD----------------------------ADGKPDLSVKV----- 247
Drosophila B        ITLMISGIRCPGVKLD----------------------------ADGKPDLSVKV----- 247
Apis                IVLMISGVRCPGWP-------------------------------NGRRENSGD------ 230
                    : : :.* :.*                                   ..

Arabidopsis 1       SSDPFATEAKYFTEHRVLSRDVRIVLEGVDKFNNLIGSVHYSDGETVKDLGLELVENGLA 329
Arabidopsis 2       SSDPFAMEAKYFTELRVLNRDVRIVLEGVDKFNNLIGSVYYSDGDTVKDLGLELVENGLA 333
Populus             APDPFGMEAKYFTELRTLNRDVRIVLEGVDKFSNLIGSVYYPDGESAKDLALELVENGLA 329
Emihu1|452958|      ----FAREARFFVESRLLHRSVQIALQGVDKNGSLLA----------------------- 265
Drosophila A        ---PFADEARYYVETRLLQRDVEIRLESVNN-SNFIGTILYPKG----NIAESLLREGLA 299
Drosophila B        ---PFADEARYYVETRLLQRDVEIRLESVNN-SNFIGTILYPKG----NIAESLLREGLA 299
Apis                ---PYADEARYFVESRLLHRDVEIVLESVNN-NNFIGSILHPKG----NIAEILLSEGFA 282
                        :. **:::.* * * *.*.* *:.*:: ..::.

Arabidopsis 1       KFVEWSANMMEEEAKKKLKAAELQCKKDKVKMWANYVPPATNSKAIHDQNFTGKVVEVVS 389
Arabidopsis 2       KYVEWSANMLDEEAKKKLKATELQCKKNRVKMWANYVPPASNSKAIHDQNFTGKVVEVVS 393
Populus             KFVEWSANMMEEDAKRQLKTAELQAKKSRLRFWTNYVPPATNSKAIHDQNFTGKVVEVVS 389
Emihu1|452958|      ----------------ELREAERQAKAGRLRLWREYVPPQAAS------ELLGRVVEVVS 303
Drosophila A        KCVDWSMAVMKTG-TDKLRAAERFAKEKRLRQWQDYQ-AKTPAFNSKEKDFSGTVVEVFN 357
Drosophila B        KCVDWSMAVMKTG-TDKLRAAERFAKEKRLRQWQDYQ-AKTPAFNSKEKDFSGTVVEVFN 357
Apis                KCQDWSINNSRAG-AEKLYLAEKAAKEARLRLWKDYK-PSGP-----QIEFTGTIVEIVN 335
                                    :*  :*  .*  ::: * :*  .          :: * :**:..
```

```
Arabidopsis         GDCLIVADDAVPFGSPAAERRVCLSSIRSP------------------KMGNPRREEKPA 431
Arabidopsis2        GDCLVVADDSIPFGSPMAERRVCLSSIRSP------------------KMGNPRREEKPA 435
Populus             GDCVIVADDSVPYGSPLAERRVNLSSIRCP------------------KMGNPRRDEKPA 431
Emihu1|452958|      GDTLVVADAAN------NETRYSLSSIRCP------------------RMGR-----EPE 334
DrosophilaA         GDAINVRLSNG------QVKKVFFSSIRPPRDQRAVVGTDGEEIVKAPPRGKNYRPLYEI 411
DrosophilaB         GDAINVRLSNG------QVKKVFFSSIRPPRDQRAVVGTDGEEIVKAPPRGKNYRPLYEI 411
Apis                ADALIIRTQNG------ENKKVFLSSIRPPSREKKTN-----EESNNTTR-KDFKPLYDI 383
                    .* : :              :  :**** *                     .

Arabidopsis 1       PYAREAREFLRQRLIGKQVIVQMEYSRKVTQGDGPTTSGAADRFMDFGSVFLPSAAKADS 491
Arabidopsis 2       PYAREAKEFLRQKLIGMEVIVQMEYSRKISPGDGVTTSGAGDRVMDFGSVFLPSPTKGDT 495
Populus             PYAREAKEFLRTRLIGRQVNVRMEYSRKMTDGPTAAPVPGDARVMDFGSIFLLSPTKGDE 491
Emihu1|452958|      PYAAEAKEALRRRVLCERVRVTPEYHRSFEG------QGGGVQERLFAAVLFDKADRNAA 388
Drosophila A        PHMFDAREFLRKKLINKKVQCNLDYISPPRE--------------NFPEKYCYTVSIGGQ 457
Drosophila B        PHMFDAREFLRKKLINKKVQCNLDYISPPRE--------------NFPEKYCYTVSIGGQ 457
Apis                PWMLEAREFLREKFIRKNVKVVVDYTQPARD--------------NFPEKLCCTVTCGKT 429
                    *   :*:* ** :.:  .*    :*                      *      .

Arabidopsis 1       DEVTAPPAAAIAGSQPVGVNIAELVLVRGFGNVVRHR-DFEERSNHYDALLAAEAR---- 546
Arabidopsis 2       ---------AVAAAATPGANIAELIISRGLGTVVRHR-DFEERSNHYDALLAAEAR---- 541
Populus             ----ASTAPSTAAGQQPGINVAELVVSRGFGTVIRHR-DFEERSNFYDALLAAESR---- 542
Emihu1|452958|      -------------------------EARRLASVSKHG-GAEERSAHYDALCDAEEHSMPR 422
Drosophila A        -------------------NVAEAMVAKGLATCVRYRQDDDQRSSAYDQLIAAEQQ---- 494
Drosophila B        -------------------NVAEAMVAKGLATCVRYRQDDDQRSSAYDQLIAAEQQ---- 494
Apis                -------------------NIAEALVARGLAKVIKYRQNDDQRSSHYNLLQVAESK---- 466
                                               : :..  ::  . ::**  *: *  ** :

Arabidopsis 1       --------ALAGKKGIHS-AKESPAMHITDLTVSAAK-KAKDFLPSLQRIRRIPAVVEYV 596
Arabidopsis 2       --------AIAGKKNIHS-AKDSPALHIADLTVASAK-KAKDFLPSLQRINQISAVVEYV 591
Populus             --------AIAGKKGIHS-AKDPPVMHITDLTTSSSK-KAKDFLPFLHRSRRISAVVEYV 592
Emihu1|452958|      GGTGCGADAIAAKKGMHSGAPPKKGAAVTDLSLPASKERAKSFLSNFTRGGKLRGVVQYV 482
Drosophila A        --------AIKGLKGLHA-KKDNATLRVNDLTVDHSR-IKVQYLPSWQRALRTEAIVEFV 544
Drosophila B        --------AIKGLKGLHA-KKDNATLRVNDLTVDHSR-IKVQYLPSWQRALRTEAIVEFV 544
Apis                --------AEKSQHGLHA-KKDIPVHRLVDLSNDPSK-AKA-FLTSLKRAQGIKAVVEFV 515
                            *  . :.:*:         : **:   ::     :*.   *     .:*::*

Arabidopsis 1       LSGHRFKLYIPKITCSIAFSFSGVRCPG------------RGEPYSEEAISVMRRRIMQR 644
Arabidopsis 2       LSGHRFKLYIPKESCSIAFAFSGVRCPG------------RGEPYSEEAIALMRRKIMQR 639
Populus             LSGHRFKLLIPKETCSIAFSFSGVRCPG------------RDEPYSEEAIALMRRKIMQR 640
Emihu1|452958|      LSGSRLKVLLLKDHCLVTLALVGVRCPACAR----RDSPSSGEPFGDEALAATRGFCLQR 538
Drosophila A        ASGSRLRIFVPKDSCLVTFLLAGISCPRSSRPALNGVPAQEGEPFGDEALTFTRERVLQR 604
Drosophila B        ASGSRLRIFVPKDSCLVTFLLAGISCPRSSRPALNGVPAQEGEPFGDEALTFTRERVLQR 604
Apis                TSGSRLKLFLPKEDQLITFVLAGIRTPRCQR-SLPGGGIVKADEYGEKALAFTREHCFQR 574
                     ** *::: : *    ::: : *:  *               : :.::*::  *   :**

Arabidopsis 1       DVEIEVETV-DRTGTFLGSMWES------RTNVATVLLEAGLAKMQTSFGADRIAEAHLL 697
Arabidopsis 2       DVEIVVENV-DRTGTFLGSMWEKN----SKTNAGTYLLEAGLAKMQTGFGADRIPEAHIL 694
Populus             DVEIEVETV-DRTGTFLGSLWES------RTNMAVTLLEAGLARFQTSFGTDRIPDAHLL 693
Emihu1|452958|      EVEVEIESVNDKTGNPRGSVFNGTLILPDKRNLSLVLLEAGLAS-RFGAAADRSVHAADL 597
Drosophila A        DVSVHIDTTDKAGSSVIGWLWTDS-----GANLSVALVEEGLAEVHFSAEKS--EYYRQL 657
Drosophila B        DVSVHIDTTDKAGSSVIGWLWTDS-----GANLSVALVEEGLAEVHFSAEKS--EYYRQL 657
Apis                DVEIKIESTETKGSGFIGWLTVN------DINMSVALVEEGLAEVVTFPDFG--ELTRTL 626
                    :*.: ::..    .   * :           * .  *:* ***        .       *

Arabidopsis 1       EQAERSAKNQKLKIWENYVEGEEVSNGNTN---------TVETRQKETLKVVVTEVLGGG 748
Arabidopsis 2       EMAERSAKNQKLKIWENYVEGEEVVNGSS----------KVETRQKETLKVVVTEVLGGG 744
Populus             EQAEQSAKRQKLKIWENYVEGEEINSGPV-----------VESKQKEVLKVVVTEVLDGG 742
Emihu1|452958|      AKSEGVAKAAGLKVWEDYSEEAAAEEAAANAAAAASEMEPIPDAQKQRVELTLTEIADGA 657
Drosophila A         KIAEDRAKAAKKNIWTNYVEEVPKEKTVTEEEKED---KVVAERKVNYENVIVTEITETL 714
Drosophila B        KIAEDRAKAAKKNIWTNYVEEVPKEKTVTEEEKED---KVVAERKVNYENVIVTEITETL 714
Apis                KAAEERAKTKKLNMWKNYVEVQVE----NEKNEND---KEIVERKIDYQEVVLSEVTEDL 679
                      :*  **    ::* :* *                    :   : :  :: ::*:
```

```
Arabidopsis 1       RFYVQSAGD-QKIASIQNQLASLSIKDAPIIGSFNPKRGDIVLAQFSLDNSWNRAMIVTA 807
Arabidopsis 2       RFYVQTVGD-QKVASIQNQLAALSLKDAPIIGSFNPKKGDIVLAQFSLDNSWNRAMIVNG 803
Populus             RFYVQIVED-KKIASIQQQLASLNLQEAPVIGAFNPKKGDIVLAQFSADNSWNRAMIVNA 801
Emihu1|452958|      HFYAHVAGD-DTVARLHAKLASACAGPPPAM-AFEPKVGAVCAARFSQDNEWYRAKVTKR 715
Drosophila A        TFFAQSVESGSKLESLMSKLHADFQSNPPIAGSYTPKRGDLVAAQFTLDNQWYRAKVERV 774
Drosophila B        TFFAQSVESGSKLESLMSKLHADFQSNPPIAGSYTPKRGDLVAAQFTLDNQWYRAKVERV 774
Apis                HFYAQSVDQRSMLENLLLQLRQELASNPPLPGAYKPTRGELAVAKFTGDDQWYRVKVEKV 739
                     *:.: . . . :  :  :*       .*   :: *. * :  *:*: *:.* *. :

Arabidopsis 1       PRAAVQSPDEKFEVFYIDYGNQETVPYSAIRPIDPSVS--AAPGLAQLCRLAYIKVPSLE 865
Arabidopsis 2       PRGAVQSPEEEFEVFYIDYGNQEIVPYSAIRPVDPSVS--SAPGLAQLCRLAYIKVPGKE 861
Populus             PRGGVESPRDKFEVFYIDYGNQEEVPYSHIRPLDPSVS--AAPGLAQLCSLAYIKVPSLE 859
Emihu1|452958|      DKG-------SYTVFFLDYGNCDVVSAERVRPLDPTLAPSVLSAQAVECRLAHLIVDEPS 768
Drosophila A        QGS-------NATVLYIDYGNKETLPTNRLAALPPAFS--SEKPYATEYALALVALPT-D 824
Drosophila B        QGS-------NATVLYIDYGNKETLPTNRLAALPPAFS--SEKPYATEYALALVALPT-D 824
Apis                SGT-------NVSVFYIDYGNREIISVTRVADLPSRFG--NDKPYAHEHILACVALPN-D 789
                              .  *:::**** : :.   :  : . ..       *    ** : :   .

Arabidopsis 1       DDFGPEAGEYLHTVTLGSGKEFKAVIEERDTSGGKVKGQGTGTEFVVTLIAVDDEISVNA 925
Arabidopsis 2       EDFGRDAGEYLHTVTLESGKEFRAVVEERDTSGGKVKGQGTGTELVVTLIAVDDEISVNA 921
Populus             DDCGPEAAQYFSDNTLNSSKELRAKVEERDASGGKVKGQGTGPVVVVTLVAVDSEISLNA 919
Emihu1|452958|      SEDGEEAALLLGDAAWG--KTVVARVEDR-ASG----------VLLVTLFDA-AQSCVNE 814
Drosophila A        NEDKEEALRAFSEDVLNHKVQLNVELKVTGSPN------------LATLRDPTTKVDFGK 872
Drosophila B        NEDKEEALRAFSEDVLNHKVQLNVELKVTGSPN------------LATLRDPTTKVDFGK 872
Apis                NDDKKAAVEIFKEDVMDKILLMNTEYKLNNNVT------------AVTLVDSSSNEDIAK 837
                    .:    *   :   .       . .  :                  .**      :  .

Arabidopsis 1       AMLQEGIARMEKRQKWGHKGKQAALDALEKFQEEARKSRIGIWQYGDIESDDEDTGPARK 985
Arabidopsis 2       AMLQEGIARMEKRRRWEPKDKQAALDALEKFQDEARKSRTGIWEYGDIQSDDEDNVPVRK 981
Populus             ALVQEGLARIEKMRKWDSMERKVALENLEKFQDEARADRRGLWVHGDIESDDEDVLPVKK 979
Emihu1|452958|      KLVAAGLARG----------------------------HVGMWRYGDIEEDDAHEFGFRR 846
Drosophila A        QLVAEGLVLAEQRGE---RKLKELVDQYKAAQEAARVAHLAIWKYGDITQDDAPEFR--- 926
Drosophila B        QLVAEGLVLAEQRGE---RKLKELVDQYKAAQEAARVAHLAIWKYGDITQDDAPEFR--- 926
Apis                GLISDGLLLVQNQRD---RRLIKLIEEYKKAEEDAKHSRRNIWRYGDIRADDEKEFGL-- 892
                     ::   *:                               :   :* :***   **

Arabidopsis 1       PAGGLEIRGSLNHAYKQKKSRD 1007
Arabidopsis 2       PGRG------------------ 985
Populus             TGGRR----------------- 984
Emihu1|452958|      PAPAPAPKGNPWKK-------- 860
Drosophila A        ----------------------
Drosophila B        ----------------------
Apis                ----------------------
```

S17 Fig. CLUSTAL (2.1) Multiple sequence alignment for the Tudor staphylococcal nuclease (TUDOR-SN) from *E. huxleyi, Populus* trichocarpa (EEF06439.1), *Apis mellifera* (XP_624638.3), *Arabidopsis thaliana* isozymes 1(NP_001154697.2) and 2 (NP_200986.1, and *Drosophila melangastor* isozymes A (NP_612021.1) and B (NP_001261195.1). Key residues in the TUDOR domain include highly conserved Arginine and Aspartate residues in green; aromatic cage residues in red, responsible for complexing di-methylated guanine groups; Asparagine in purple, involved in the binding and interaction with PIWI proteins by means of the symmetric di-methylation of distinct arginine (sDMA) residues; and a strongly conserved Glycine in in blue.
