# Supplementary material for: Characterization of the Small RNA Transcriptome of the Marine Coccolithophorid, Emiliania huxleyi
Source: PLoS One. 2016 Apr 21;11(4):e0154279. doi: 10.1371/journal.pone.0154279 (PMC4839659; doi:10.1371/journal.pone.0154279)
Supplement: S1 Table — (DOC) [file pone.0154279.s020.doc]

S1 Table. Homology search results for known miRNAs.

| **MirBase ID** | **Sequence** | **miRNA length** | **Precursor length** | **Scaffold** | **Strand** | **Mismatch** |
| --- | --- | --- | --- | --- | --- | --- |
| cre-miR1171 | TGGAGTGGAGTGGAGTGGAGTGG | 23 | 82 | scaffold_8 | + | 0 |
| mmu-miR-324-3p | CCACTGCCCCAGGTGCTGCT | 20 | 95 | scaffold_279 | + | 1 |
| hsa-miR-920 | GGGGAGCTGTGGAAGCAGTA | 20 | 110 | scafflold_5 | + | 0 |
| oan-miR-1331 | TGGGGTGGGAGGTTGGTGTGTG | 22 | 69 | scaffold_4819 | + | 0 |
| oan-miR-1412 | TGGGTGAGGAGGGCGAGG | 18 | 90 | scaffold_173 | + | 1 |
| gga-miR-1557 | CCCGTCGGCTGAGCGGCTGC | 20 | 71 | scaffold_17 | + | 1 |
| mmu-miR-1893 | GGCGCGGGCGCTGGACGCCTCG | 22 | 100 | scaffold_22 | + | 1 |
| ppt-miR2078 | GGTTGGCTTGCCTGTGCCTGT | 21 | 66 | scaffold_880 | - | 1 |
| gga-miR-1648 | CGGCTCGGCTCGGCTCCGCTC | 21 | 130 | scaffold_5 | + | 1 |
| hsa-miR-1268 | CGGGCGTGGTGGTGGGGG | 18 | 110 | scaffold_48 | - | 1 |
| mml-miR-297 | ATGTATGTGTGCATGTGCAT | 20 | 160 | scaffold_164 | - | 1 |
| gma-miR1533 | ATAATAAAAATAATAATGA | 19 | 114 | scaffold_232 | + | 1 |
| hsa-miR-1281 | TCGCCTCCTCCTCTCCC | 17 | 219 | scaffold_24 | + | 1 |
| oan-miR-1346 | GTGGGTTGGGGGCGGGGG | 18 | 180 | scaffold_152 | - | 1 |
| hsa-miR-297 | ATGTATGTGTGCATGTGCATG | 21 | 194 | scaffold_164 | - | 1 |
| hsa-miR-1322 | GATGATGCTGCTGATGCTG | 19 | 130 | scaffold_87 | + | 1 |
| oan-miR-1408 | CGGGGAGGGAGGGTGGTG | 18 | 111 | scaffold_41 | - | 1 |
| mmu-miR-466f | ACGTGTGTGTGCATGTGCATGT | 22 | 95 | scaffold_381 | - | 1 |
