# Supplementary material for: Characterization of the Small RNA Transcriptome of the Marine Coccolithophorid, Emiliania huxleyi
Source: PLoS One. 2016 Apr 21;11(4):e0154279. doi: 10.1371/journal.pone.0154279 (PMC4839659; doi:10.1371/journal.pone.0154279)
Supplement: S2 Table — (DOC) [file pone.0154279.s021.doc]

S2 Table. Sequences and Loci of miRNA candidate precursors

| **ID** | **Length** | **Precursor Sequences** | **Loci** |
| --- | --- | --- | --- |
| mir01 | 82 | GTGGAGTGGAGTGGAGTGGAGTGGGAGGGGGAGAGTGGCACGGCGCCTCCTCGAAGCTTCCAGCGGCGCGCTTCCGCTTCGG | scaffold_8(+) (38755, 38836) |
| mir02 | 170 | CAAGCTCAACAGGACTGAGCGCGCCCCGCGTCCCCGCCCCTCCGCCCCGCCCCCCCGCCCCGCCCCCCCGGAGCGCACCCTATTGGCACGCTGGCGCAGCGGCACTCCCGAGGCTGCCCCGGTTACTAAGGGTGCGCGTCAGCGATGCGCGTCCTAATCCTTGGCCTGGC | scaffold_166(-) (146651, 146820) |
| mir03 | 193 | CTCGTGCTCTCCCTTGCTCTCCCTCGTCGCAGATTTACTTTACTGAGTCTATTGAGGCGAGATCTCCCGCTGTTTCTCCGCATGCGGCCATATCACCGTGGGTTACCGCTTCCCATCTGAACAGCGAAGTCAAGCACGGTCGAGCTCAGGTAGTACTTCCATCCGGGAGGGGGAGGGAAGGCTGAGTGCTGCA | scaffold_56(-) (40353, 40545) |
| mir04 | 252 | CAGCGTGCGCTGCACCAGCTGCTCAAGCTGCTCCTCGTGCCGCCGCTCGACGCGCTGCTTGCCCAGTTTGTTCCACAAGTCATTTTCCCAGATACAGTGCGTACATGGTGTTCCGGCCTCAACAAAGTTGGTACATGCGGCCATATCACCGTGGGTTACCGCTTCCCATCTGAACAGCGAAGTCAAGCACGGTCGAGCTCAGGTAGTACTTCCATCCGGGAGGGGGAGGGAAGGCTGAGTGCTGCATGTTTT | scaffold_26(+) (199949, 200200) |
| mir05 | 79 | AGGCTCGAGGCCGGCTGGCTGCGCGGGGACTACGGCCTCACGGCTGCGGATCCCGCGCGGCCGTCCGGCGGACGGCCTA | scaffold_1642(+) (3944, 4022)  scaffold_1054(+) (2804, 2882)  scaffold_341(+) (72796, 72874)  and 16 more |
| mir06 | 76 | GTTGTTTGATGAGATGTGCAGAAACTCACGGACCTCGACCTCATCGAGTTGTTGTTTTTGTGACCTCATCCTCAAC | scaffold_4(+) (228434, 228509) |
| mir07 | 82 | GGGTCGGTGGTAGTGGATAGCGTGTTGGGCGAACAAAGGGCGGAGTCGCGACGCTCCGCCGCTGCCACGTTCGGCCGGACCC | scaffold_12(-) (920068, 920149) |
| mir08 | 114 | GAGTGGTGCAACCATGTGGCGTCGGCACAGGCGGCGTGGGCGATGCCTCCAGTGCTGGAGCCCTCCGCCGCGGCCGCCGCGGACCCGGCCGACCTCCACGTGGTCTTTTCCGCC | scaffold_22(+) (918300, 918413) |
| mir09 | 80 | GCGGCCCTCGAACGGACACCGGGCCGCCACCTTGGCCCGGGCGAGGCGGTTTAGTCGCCGCCCGTCCTTTGCACGGCCGC | scaffold_24(+) (576679, 576758) |
| mir10 | 144 | CCGCGGGCGGTGCAGAGATCGCGGGGGAGGGAGACAGCTCGTGTCGCTCTCTTAGACTTAGTTGAGCTAAGACAAAACAAAGTGTTTTGGAGGTGCGGAGGAGATGTGCCGTTCCGAGGGGATTCTGTAAATTGTATTCCGCGG | scaffold_34(+) (494448, 494591) |
| mir11 | 119 | GCTTGCTCCCCAACGATGCATGTAGGTCGCGAACGGGTTTTGGCTCCCGTGTGTACCTGTGTGTAGTGCCAGTGCGCGCCAGTCCCGTGCGTGCCCAGTGTCGTGCGAGGGAGTTTTGT | scaffold_55(+) (487472, 487590) |
| mir12 | 94 | GCTCCGCCGGTCAGTGAGGACGCTGTATCAAGGGTTGTGATAGACCCGTCAAGCCAGCCACTCCCTGGCGATGCCGCGCCACCTGCCGGGGGGC | scaffold_58(-) (440458, 440551) |
| mir13 | 93 | GGGCCCTGACACGTCAATTAGCGCCAGCCGCGCCGGGCGCTCACACCGAGTGCTCCTTCTGCCGGCTGGCGTCAGTCGACCGTGGCACATCCC | scaffold_5051(-) (243, 335)  scaffold_167(-) (33309, 33401) |
| mir14 | 89 | CGAGGTGTCCGAAGATGTATGTGCGGTGTCCTTGCAGCCCATGTTTTGTTGTTTTGTTGTTTGAGACACACACATATCTAGATATCTCG | scaffold_241(-) (132396, 132484) |
| mir15 | 127 | AGACGGGATGGTGTCCGAAGCAGCACCGTCGAGACCCGGGTTCAATTCCCGGAGGGGAAGTCTACGGGGCAGCATGCCCCTGAGGTTCTCCTTAGTATAGTGGCTAGTATCCGACGCTGTCAATTCT | scaffold_2199(-) (2739, 2865) |
| mir16 | 92 | GTGAAGAGGTTATCGCGGTTACGTCATACGTGCCCTTCTTTCGCCTTTTACTAAAGAAGGCCGTGGCGCAACTGCCTAACGACCTCTTCCAC | scaffold_1118(+) (3811, 3902) |
| mir17 | 85 | GTGTAGGTGCGGTGCGGGGCTGCCGATTCATCGGTGGTGCTCAGTCAAGATATTGTCGGTCTCCTCGCTAGACTTGTACTTATAC | scaffold_414(-) (42052, 42136) |
| mir18 | 95 | GTGTGCGTTGATGAGGCCGATCTGGTCTAGGAGCACGGTTTCCAAGTGCTCCAAGTGCACCTAGAGAGGTGATCCGAGGCGGCTTCACAGCACAC | scaffold_414(-) (42827, 42921) |
