# Supplementary material for: Characterization of the Small RNA Transcriptome of the Marine Coccolithophorid, Emiliania huxleyi
Source: PLoS One. 2016 Apr 21;11(4):e0154279. doi: 10.1371/journal.pone.0154279 (PMC4839659; doi:10.1371/journal.pone.0154279)
Supplement: S3 Table — (DOC) [file pone.0154279.s022.doc]

**S3 Table. Blastn homology search results comparing** miRNA precursors to the non-coding sequences in the European Nucleotide Archive (ENA) and NCBI nucleotide collection (NR/NT).

| **miRNA Precursor ID** | **ENA non-code** | **Alignment length** | **Identity (%)** | **NR Database**  **Top hit** | **coverage** |
| --- | --- | --- | --- | --- | --- |
| mir01 | NO HIT |  |  | NO HIT |  |
| mir02 | NO HIT |  |  | NO HIT |  |
| mir03 | NO HIT |  |  | NO HIT |  |
| mir04 | NO HIT |  |  | Emiliania huxleyi CCMP1516 hypothetical protein (EMIHUDRAFT_116406) | 30% |
| mir05 | NO HIT |  |  | NO HIT |  |
| mir06 | NO HIT |  |  | NO HIT |  |
| mir07 | NO HIT |  |  | NO HIT |  |
| mir08 | NO HIT |  |  | Emiliania huxleyi CCMP1516 hypothetical protein (EMIHUDRAFT_233789) | 63% |
| mir09 | NO HIT |  |  | NO HIT |  |
| mir10 | NO HIT |  |  | NO HIT |  |
| mir11 | NO HIT |  |  | Emiliania huxleyi CCMP1516 hypothetical protein (EMIHUDRAFT_204650) | 38% |
| mir12 | NO HIT |  |  | NO HIT |  |
| mir13 | NO HIT |  |  | NO HIT |  |
| mir14 | NO HIT |  |  | NO HIT |  |
| mir15 | Sordaria macrospora k-hell tRNA-Asp | 30 | 96% | NO HIT |  |
| mir16 | NO HIT |  |  | NO HIT |  |
| mir17 | NO HIT |  |  | NO HIT |  |
| mir18 | NO HIT |  |  | NO HIT |  |
