# Supplementary material for: Characterization of the Small RNA Transcriptome of the Marine Coccolithophorid, Emiliania huxleyi
Source: PLoS One. 2016 Apr 21;11(4):e0154279. doi: 10.1371/journal.pone.0154279 (PMC4839659; doi:10.1371/journal.pone.0154279)
Supplement: S4 Table — The table shows the identity of alignment of the precursors in the other strains (all alignments have e-value < 1e-10). (DOC) [file pone.0154279.s023.doc]

S4 Table. MiRNA precursors in three other strains of *E. huxleyi*. The table shows the alignments and e-values of the precursors in the other three strains.

| **miRNA Precursor ID** | **92A** | **EH2** | **Van556** |
| --- | --- | --- | --- |
| mir01 | 3.E-35 | 4.E-20 | 1.E-20 |
| mir02 | - | - | - |
| mir03 | 1.E-53 | 1.E-53 | 1.E-53 |
| mir04 | 2.E-77 | 2.E-57 | 2.E-57 |
| mir05 | 3.E-35 | 4.E-35 | 3.E-35 |
| mir06 | - | - | - |
| mir07 | 6.E-32 | 8.E-27 | 2.E-33 |
| mir08 | 9.E-37 | 5.E-31 | 5.E-35 |
| mir09 | 7.E-36 | 2.E-22 | 5.E-24 |
| mir10 | - | - | - |
| mir11 | 3.E-22 | 6.E-15 | 1.E-17 |
| mir12 | - | - | 2.E-24 |
| mir13 | 5.E-43 | 8.E-43 | 7.E-43 |
| mir14 | - | - | - |
| mir15 | 5.E-60 | 2.E-59 | 3.E-48 |
| mir16 | 4.E-39 | 6.E-39 | 5.E-39 |
| mir17 | 2.E-12 | 3.E-12 | 2.E-12 |
| mir18 | - | 1.E-09 | 3.E-11 |
