# Supplementary material for: Characterization of the Small RNA Transcriptome of the Marine Coccolithophorid, Emiliania huxleyi
Source: PLoS One. 2016 Apr 21;11(4):e0154279. doi: 10.1371/journal.pone.0154279 (PMC4839659; doi:10.1371/journal.pone.0154279)
Supplement: S5 Table — The table shows the percentage of matched bases of the miRNA candidates to the strain genomes. (DOC) [file pone.0154279.s024.doc]

S5 Table. Mature miRNA in three other strains of *E. huxleyi*. The table shows the percentage of matched bases of the miRNA candidates to the strain genomes.

| **miRNA ID** | **92A** | **EH2** | **Van556** |
| --- | --- | --- | --- |
| **mir01** | 95.7% | 95.7% | 100.0% |
| **mir02** | 68.4% | 68.4% | 94.7% |
| **mir03** | 100.0% | 100.0% | 100.0% |
| **mir04** | 100.0% | 100.0% | 100.0% |
| **mir05** | 100.0% | 100.0% | 100.0% |
| **mir06** | 71.4% | 71.4% | 71.4% |
| **mir07** | 94.4% | 94.4% | 94.4% |
| **mir08** | 66.7% | 66.7% | 76.2% |
| **mir09** | 100.0% | 100.0% | 100.0% |
| **mir10** | 76.2% | 71.4% | 71.4% |
| **mir11** | 66.7% | 66.7% | 66.7% |
| **mir12** | 70.8% | 58.3% | 58.3% |
| **mir13** | 100.0% | 100.0% | 100.0% |
| **mir14** | 71.4% | 66.7% | 61.9% |
| **mir15** | 100.0% | 100.0% | 100.0% |
| **mir16** | 100.0% | 100.0% | 100.0% |
| **mir17** | 81.0% | 81.0% | 81.0% |
| **mir18** | 81.0% | 81.0% | 81.0% |
