# Supplementary material for: Characterization of the Small RNA Transcriptome of the Marine Coccolithophorid, Emiliania huxleyi
Source: PLoS One. 2016 Apr 21;11(4):e0154279. doi: 10.1371/journal.pone.0154279 (PMC4839659; doi:10.1371/journal.pone.0154279)
Supplement: S6 Table — (DOC) [file pone.0154279.s025.doc]

S6 Table. Predicted miRNA target genes amongst the genes differentially expressed between life-cycles, up-regulated in haploid (1N) and up-regulated in dipoild (2N) respectively [37].

| **miRNA ID** | **Targets up-regulated in 1N** | **Targets up-regulated in 2N** |
| --- | --- | --- |
| mir01 |  | 434999  446970 |
| mir02 | 221997 |  |
| mir03 | 413787  98528  97320  61076 | 95349  465079  442233  435993  470658  462433  360563  363940  357728  457256  454547 |
| mir04 | 234887 |  |
| mir05 | 437912  449865  206752  434188 |  |
| mir07 | 373550 | 438119 |
| mir08 |  | 441887 |
| mir09 | 195122 | 43239  198567 |
| mir10 | 122556  199879  224944  350739  439552 | 470970 |
| mir11 | 235936 | 463240  203652 |
| mir12 |  | 434204 |
| mir13 |  | 435830 |
| mir14 | 208664 | 61253  116540  239538  251683 |
| mir16 | 224407 |  |
| mir17 | 248510  208412 | 221128  196106 |
| mir18 | 104019  457149  63754  237172  198292 |  |
