# Supplementary material for: Characterization of the Small RNA Transcriptome of the Marine Coccolithophorid, Emiliania huxleyi
Source: PLoS One. 2016 Apr 21;11(4):e0154279. doi: 10.1371/journal.pone.0154279 (PMC4839659; doi:10.1371/journal.pone.0154279)
Supplement: S7 Table — (DOC) [file pone.0154279.s026.doc]

**S7 Table. Characteristics of stem-loop quantitative real time PCR mi**RNA primers and their corresponding amplification products.

| **gene** | **primer (5' to 3')** | **Product Length** | **Estimated Tm** | **Actual Tm** | **Efficiency** |
| --- | --- | --- | --- | --- | --- |
| mir01 | TGGAGTGGAGTGGAGTGGA | 70 | 76.4 | 79.0 | 96.4 |
| mir02 | CAGGCCAAGGATTAGGACG | 66 | 75.6 | 80.5 | 99.2 |
| mir03 | AGCCTTCCCTCCCCCTCCCG | 67 | 78.6 | 80.5 | 98.6 |
| mir04 | ACATGCAGCACTCAGCCTTCC | 68 | 75.3 | 80.5 | 98.4 |
| mir05 | CGTAGTCCCCGCGCAGCCAGCCG | 70 | 79.2 | 82.0 | 99.1 |
| mir06 | GTCGAGGTCCGTGAGTTTCTG | 68 | 75.3 | 80.0 | 98.4 |
| mir07 | CCCAACACGCTATCCACT | 65 | 75.3 | 78.5 | 98.3 |
| mir08 | TGTGCCGACGCCACATGGTTG | 68 | 76.2 | 81.5 | 95.8 |
| Mir09 | CCGGTGTCCGTTCGAGGGCCGCGG | 71 | 79.4 | 83.0 | 98.7 |
| mir10 | CGGAACGGCACATCTCCTCCG | 68 | 77.0 | 81.0 | 97.2 |
| mir11 | AACCCGTTCGCGACCTACATG | 68 | 75.3 | 80.5 | 93.7 |
| mir12 | CCTTGATACAGCGTCCTCACTGAC | 71 | 74.5 | 77.5, 79.0 | 96.2 |
| mir13 | GGATGTGCCACGGTCGACTGACGC | 71 | 76.9 | 82.0 | 98.1 |
| mir14 | CATACATCTTCGGACACCTCG | 68 | 74.1 | 79.5 | 97.8 |
| mir15 | GACAGCGTCGGATACTAGCCAC | 68 | 76.2 | 81.5 | 96.7 |
| mir16 | CAGTTGCGCCACGGCCTTCTT | 70 | 75.0 | 79.0 | 97.0 |
| mir17 | ACCGATGAATCGGCAGCCCCG | 68 | 77.0 | 81.5 | 98.2 |
| mir18 | ACCAGATCGGCCTCATCAACG | 68 | 75.3 | 80.5 | 97.5 |
| Universal Rev | TGTCAGGCAACCGTATTCACC | NA | NA | NA |  |
| Stem-Loop | TGTCAGGCAACCGTATTCACCGTGAGTGG(T)18 | NA | NA | NA |  |
| U6 For | GACTTCTTCTCGAGTC | 94 | 73.3 | NA |  |
| U6 Rev | AACGGATGCTTGCAGAGCAA | NA | NA | NA |  |
