# Supplementary material for: Characterization of the Small RNA Transcriptome of the Marine Coccolithophorid, Emiliania huxleyi
Source: PLoS One. 2016 Apr 21;11(4):e0154279. doi: 10.1371/journal.pone.0154279 (PMC4839659; doi:10.1371/journal.pone.0154279)
Supplement: S8 Table — (DOC) [file pone.0154279.s027.doc]

**S8 Table. Real-Time PCR primer sets and features of the amplification products for a select set of mir target genes.**

| **PID** | **Left Primer** | **Right Primer** | **Product Size (bp)/% G+C** | **Estimated T_m_** | **Actual T_m_** | **Efficiency** |
| --- | --- | --- | --- | --- | --- | --- |
| **231239** | GTGTCTCCCGCAGCCTTC | GTCTCTAGGCCAACCGACTG | 98/69.4 | 89.8 | 85.5 | 0.96 |
| **434327** | CGACTTTCAGCAAAAGAAGGAG | CTCAACAGCCTTGGTTGATCC | 85/56.5 | 84.4 | 85.5 | 0.99 |
| **228827** | CAGTGGATTGCAGACTACCTTG | AGTTCATGATCATCGCCTTGTA | 85/54.1 | 86.1 | 85.5 | 1.07 |
| **234052** | GAATTTAACTCCACGCCCTACA | AAAATCGTACCACTCGAGGAAA | 96/56.3 | 84.7 | 84.5 | 0.99 |
| **351247** | CTGACAAGGTAAACGCACTTTG | GGCTGAGAGTAGCCAACACC | 85/56.4 | 87.5 | 87.0 | 0.97 |
| **363229** | CTGACAAGGTAAACGCACTTTG | GGTGACGGGGCTAGAGAGTAG | 94/57.4 | 85.6 | 88.5 | 1.06 |
| **370380** | TGTGTGAGAGTGTGCTGTCTGT | TATGAGAGGAGACACAGCCTGA | 82/57.3 | 88.4 | 86.5 | 1.02 |
| **373560** | TGTGTGAGAGTGTGCTGTCTGT | TATGAGAGGAGACACAGCCTGA | 82/51.2 | 85.3 | 86.0 | 0.99 |
| **373620** | TGTGTGAGAGTGTGCTGTCTGT | TATGAGAGGAGACACAGCCTGA | 82/57.3 | 80.8 | 86.0 | 0.98 |
| **421687** | ATGATCGTCGTCTCCAACG | CCTGTCGCAGGATATCGTCT | 97/63.9 | 87.7 | 88.5 | 0.98 |
| **45241** | GACCTCGTCAACGTGCTCTC | GGGTGTGCCTTCCTCAGC | 98/72.4 | 91.6 | 91.5 | 0.96 |
| **101420** | CGACGACCAGTCCATCAATA | TTTGAGGAAGGTGAGCCACT | 97/62.9 | 87.3 | 89.5 | 1.01 |
| **101602** | ATTTACCGAAAGGGCGTGTT | CCCACCAGACGATCTTGTTC | 100/56 | 83.8 | 82.0 | 0.99 |
| **101738** | AACCACGTCGTCTTCGAGTC | ACCGTCACATCCTTCTCCTG | 98/64.3 | 87.6 | 85.0 | 0.97 |
| **111298** | ATTTACCGAAAGGGCGTGTT | CCCACCAGACGATCTTGTTC | 100/57 | 83.8 | 86.5 | 0.98 |
| **204818** | GGCCCTCTTCTACGTCTCCT | TCCTCCTCCACCATCTTGTC | 93/58 | 86.1 | 88.0 | 0.99 |
| **421687** | ATGATCGTCGTCTCCAACG | CCTGTCGCAGGATATCGTCT | 97/63.9 | 89.9 | 88.5 | 0.98 |
| **427008** | CACTTCACCCTCATCCTGCT | CACGTGGTTGATTGTCTCGT | 87/62.1 | 89.3 | 88.0 | 0.96 |
| **433725** | CCAAGTACGAGGGGTTCTGA | CACAGCAACGAACCTCTCAA | 76/48.7 | 85.5 | 82.0 | 1.00 |
| **445511** | TCGACCTTCCTGTGCTATGA | ATCGCTGTCCCTTCTCCTCT | 91/63.8 | 89.1 | 88.5 | 0.97 |
| **453665** | CGAAGGGCGATAACAAGTTC | GCTGTCCTTGACGCTCTTCT | 77/58.4 | 90.4 | 88.5 | 0.96 |
| **454604** | CAATGAATGGCTCGTTAGGC | CGCTCATGAACCAGCAGTC | 75/66.7 | 94.6 | 90.0 | 0.99 |
| **462160** | TTTGTCCTCTGGCTCTCGAT | ACTCGTACTCGCGCATCTTT | 85/67 | 92.3 | 90.5 | 1.07 |
| **464593** | CGCAGGTAGAGATCGAGGTG | CCCCAGTCGTGGTATGAGAG | 85/63.5 | 90.6 | 86.5 | 0.98 |
| **436024** | TAAAGATCGACTGCCCGAAC | GCGACGTGATCTCCTCAAAC | 78/52.6 | 87.1 | 85.0 | 0.97 |
| **465929** | CCTTCCAGGAGTACGTCGAG | GAGAGAAAATGGCCATGAGG | 84/64.3 | 91.3 | 88.5 | 0.98 |
| **56457** | TTGGGCACGATAAGGACTTC | GAGGGAGGAGGAGCAGGA | 98/67.3 | 88.9 | 87.0 | 0.97 |
| **96880** | CTGTCCAACTCGATCAAGCA | CACCTTGAAGACCTCGTCCT | 87/63.2 | 87.7 | 89.0 | 0.99 |
