# Supplementary material for: Characterization of the Small RNA Transcriptome of the Marine Coccolithophorid, Emiliania huxleyi
Source: PLoS One. 2016 Apr 21;11(4):e0154279. doi: 10.1371/journal.pone.0154279 (PMC4839659; doi:10.1371/journal.pone.0154279)
Supplement: S9 Table — (DOC) [file pone.0154279.s028.doc]

S9 Table. The predicted groups of Trans-acting siRNAs (ta-siRNAs) candidates.

| **Loc ID** | **Candidate ta-siRNA Reads** | **# of Loci** | **Region** | **P-value** | **Loci** |
| --- | --- | --- | --- | --- | --- |
| TAS01 | CGAACCTCGACAGCGCTTTCG (sir15)  GTGGCATGCAGCTCTTTGCGA (sir34)  GTTGACGGAAATCGGGGGACG (sir35) | 2 | intergenic | 8.20E-05 | scaffold_1473 (1160, 1411)  scaffold_92 (432795, 433046) |
| TAS02 | CAACGACTGGGACACCAACGC (sir10)  GCAGGATTTTGGAAGGACGAT (sir26)  GTTGGTGTCCCAGTCGTTGGT (sir36) | 1 | gene boundary | 3.18E-04 | scaffold_192 (6229, 6480) |
| TAS03 | CGGTACGAGTAGTGCAGGGTG (sir19)  CGTGCGGCTTCAGCAACGAGT (sir20)  GCTACGTCGACCCGACCGCGT (sir29) | 1 | intergenic | 7.72E-04 | scaffold_23 (601336, 601587) |
| TAS04 | ACAAGGTCTCGGCGCAGTTCG (sir02)  ACTACGACGTCGACGGCAAGG (sir06)  AGGTCGGCGCGCTCGAGGTCG (sir09) | 1 | exon | 3.18E-04 | scaffold_4 (1007142, 1007393) |
| TAS05 | CACGGGTGAGCGCAGCGTCGA (sir12)  CGACGCGAGCGTGGGACACCG (sir17)  CGGAGCAGGAGTAGTAGATGG (sir18) | 9 | exon | 7.72E-04 | scaffold_104 (134526, 134777)  scaffold_12 (927158, 927409)  … |
| TAS06 | AGAGAGGTCGCTTGTGCACGC (sir08)  CGACAACGACGCCTGGAGCAA (sir16)  GCTCAAGCGGCAGCTCTGCGA (sir30) | 3 | intergenic | 3.18E-04 | scaffold_1184 (4490, 4741)  scaffold_12 (1036956, 1037207)  scaffold_12 (1045605, 1045856) |
| TAS07 | GACGACTCGACGCCGCTGAGA (sir24)  GCAGATCACATCAGGACACGG (sir25)  GGGTGAGTCACGTCGGCGTCA (sir32) | 3 | intergenic | 3.18E-04 | scaffold_10 (732664, 732915)  scaffold_361 (56827, 57078)  scaffold_56 (57742, 57993) |
| TAS08 | AAGGAGCTCAAGGCAAAGTCG (sir01)  CTGATGAAGGAGATCCTCGGC (sir22)  TCGCCCTGCGTGCTGGTGACG (sir37) | 1 | exon | 7.72E-04 | scaffold_130 (214606, 214857) |
| TAS09 | ACCCAGGAGTGGTCGGGCGGT (sir04)  CACGCTGTCGACAGGACGTCG (sir11)  CATGGTCTGTGTCGCTGTCGG (sir13) | 119 | intergenic | 3.18E-04 | scaffold_1 (1599286, 1599537)  scaffold_10 (885014, 885265)  … |
| TAS10 | ACACACTCTGCACAGTTGACA (sir03)  ACTTGTGCTGCGGCTTGTGTG (sir07)  CTCACAGTGTGCCGGCAGTCG (sir21) | 2 | intergenic | 8.20E-05 | scaffold_4 (1606489, 1606740)  scaffold_59 (652928, 653179) |
| TAS11 | CGAACCTCGACAGCGCTTTCG (sir15)  GTGGCATGCAGCTCTTTGCGA (sir34)  TGACGGAAATCGGAGGACGCG (sir38) | 1 | intergenic | 7.72E-04 | scaffold_691 (7649, 7900) |
| TAS12 | CAACGACTGGGACACCAACGC (sir10)  CCAAGGGAGGGATGGGAGGAC (sir14)  GTTGGTGTCCCAGTCGTTGGT (sir36) | 1 | intergenic | 8.20E-05 | scaffold_16 (167357, 167608) |
| TAS13 | CAACGACTGGGACACCAACGC (sir10)  GCTGCTGAGGCGAGAGTCGGA (sir31)  GTTGGTGTCCCAGTCGTTGGT (sir36) | 1 | gene bounary | 3.18E-04 | scaffold_26 (414461, 414712) |
| TAS14 | CAACGACTGGGACACCAACGC (sir10)  CTGGACAGATGATGACGACAC (sir23)  GTTGGTGTCCCAGTCGTTGGT (sir36) | 1 | intergenic | 3.18E-04 | scaffold_1 (357505, 357756) |
| TAS15 | ACGGGTGAGTCACGTCGGCGT (sir05)  GACGACTCGACGCCGCTGAGA (sir24)  GCAGATCACATCAGGACACGG (sir25) | 1 | intergenic | 3.18E-04 | scaffold_330 (13180, 13431) |
| TAS16 | GCGACTGGAAGCACGCGCGCG (sir27)  GCGAGGCGGACCGGAAGGAGT (sir28)  GGTTCAACTACTCGATGGACA (sir33) | 1 | exon/intron boundary | 7.72E-04 | scaffold_313 (43895, 44146) |
