# Supplementary material for: Characterization of the Small RNA Transcriptome of the Marine Coccolithophorid, Emiliania huxleyi
Source: PLoS One. 2016 Apr 21;11(4):e0154279. doi: 10.1371/journal.pone.0154279 (PMC4839659; doi:10.1371/journal.pone.0154279)
Supplement: S10 Table — (DOC) [file pone.0154279.s029.doc]

S10 Table. Possible candidates for *E. huxleyi* RNAi components.

| **Gene Description** | **Gene Name** | **Protein ID** | **Domains** | **% Identity** | **E-value** | **RefSeq Homolog** |
| --- | --- | --- | --- | --- | --- | --- |
| **Dicer-like** | DCL | 111240 | Helicase_C, DEAD, dsrm | 27 | 2.8e-37 | Dicer homolog 2a, [*Oryza sativa Japonica* Group] |
|  | DCL2 | 243234 | Helicase_C, DEAD | 55 | 5.8e-113 | TPA: Fanconi anemia, complementation group M [*Bos taurus]* |
|  | DCL3 | 223527 | DEAD | 42 | 6.5e-13 | dicer [*Schizosaccharomyces japonicas*] |
|  |  | 110711 (465455^1^) | dsRDB (Dsrm), RNase III | 28 | 0.002 | Ribonuclease 3 [Microgenomates (Collierbacteria) bacterium] |
|  |  | 121827 | dsRDB (Dsrm), RNase III | 36 | 3e-15 | Ribonuclease 3 [Parcubacteria bacterium] |
| **Argonaute** | AGO | 226029 (253206[[1]](#footnote-2), 211169^1^) | Paz, Piwi | 29 | 6.4e-68 | argonaute 1 [*Nematostella vectensis*] |
|  |  | 46005 | Piwi | 42 | 3.6e-36 | protein argonaute-2 [*Clonorchis sinensis*] |
|  |  | 414846 (430174^1^) | Piwi | 36 | 6.3e-44 | argonaute 1 [*Nematostella vectensis*] |
| **Argonaute-like** |  | 207816 | PAZ |  |  |  |
| **RNA dependent RNA** | RDR | 205162 | RdRP | 31 | 7.3e-54 | Rna-dependent RNA polymerase [*Medicago truncatula*] |
| **polymerase** |  | 216785 | RdRP | 31 | 2.2e-19 | rna-dependent RNA polymerase [Populus trichocarpa] |
|  |  | 99932 | Zf-CCHC;RdRP | 27 | 5.1e-32 | probable RNA-dependent RNA polymerase 5-like [Vitis vinifera] |
| **Methyltransferase** | HEN1 | 454426 | Ribonuclease_3, Methyltransf_11, HEN1 | 34 | 1e-25 | methyltransferase type 12 [Deinococcus phoenicis] |
| **Armitage, SDE3** |  | 247007 | AAA_11;AAA_12 | 42 | 1.9e-145 | RNA helicase [*Physcomitrella patens*] |
|  |  | 436918 | AAA_11;AAA_12 | 64 | 4.1e-84 | putative helicase Mov10l1 isoform X13 [*Macaca fascicularis*] |
|  |  | 119956 | CSD; DNA_pol_A exo-1, AAA_11;AAA_12 | 56 | 2.8e-145 | putative helicase mov-10-B.1-like [*Ciona intestinalis*] |
| **TUDOR-SN** | TSN | 452958 | TUDOR; Snase | 32 | 3.6e-133 | 110 kDa 4SNc-Tudor domain family protein [*Populus trichocarpa*] |

1. Paralog genes [↑](#footnote-ref-2)
