# Supplementary material for: Characterization of the Small RNA Transcriptome of the Marine Coccolithophorid, Emiliania huxleyi
Source: PLoS One. 2016 Apr 21;11(4):e0154279. doi: 10.1371/journal.pone.0154279 (PMC4839659; doi:10.1371/journal.pone.0154279)
Supplement: S11 Table — The table shows the e-value of blastn search of candidate RNAi genes against the genomes of three other E. huxleyi strains. (DOC) [file pone.0154279.s030.doc]

S11 Table. Analysis of candidate *E. huxleyi* RNAi components in three other strains. The table shows the e-value of blastn search of candidate RNAi genes against the genomes of three other *E. huxlyei* strains.

| **Gene Description** | **Protein ID** | **EH2** | **92A** | **Van556** |
| --- | --- | --- | --- | --- |
| **Dicer-like** | 111240 | 0 | 0 | 0 |
|  | 243234 | 0 | 1e-173 | 0 |
|  | 223527 | 3e-104 | 3e-100 | 2e-94 |
|  | 110711 | 5e-145 | 1e-125 | 0 |
|  | 121827 | 2e-101 | 2e-65 | 2e-32 |
| **Argonaute** | 226029 | 0 | 0 | 0 |
|  | 46005 | 6e-19 | 2e-155 | 9e-93 |
|  | 414846 | 2e-122 | 3e-132 | 1e-149 |
| **Argonaute-like** | 207816 | 3e-47 | 4e-95 | 4e-75 |
| **RNA dependent RNA**  **polymerase** | 205162 | 3e-93 | 6e-84 | 2e-93 |
|  | 216785 | NA | 3e-114 | 0 |
|  | 99932 | 0 | 0 | 0 |
| **Methyltransferase** | 454426 | 6e-53 | 2e-80 | 4e-91 |
| **Armitage, SDE3** | 247007 | 0 | 0 | 3e-45 |
|  | 436918 | 2e-65 | 6e-68 | 1e-95 |
|  | 119956 | 0 | 0 | 2e-50 |
| **TUDOR-SN** | 452958 | 6e-125 | 5e-101 | 8e-117 |
